# Determining Risk Factors Associated with Cardiovascular Complications in Patients with Acute Leukemia: A Systematic Review

**DOI:** 10.3390/cancers17172777

**Published:** 2025-08-26

**Authors:** Arezoo Abasi, Haleh Ayatollahi, Soroush Rad, Marjan Hajahmadipoor Rafsanjani

**Affiliations:** 1 Student Research Committee, Iran University of Medical Sciences, Tehran 14496-14535, Iran; abbasi.ar@iums.ac.ir; 2 Health Management and Economics Research Center, Health Management Research Institute, Iran University of Medical Sciences, Tehran 19967-13883, Iran; 3 Hematology, Oncology and Stem Cell Transplantation Research Center, Research Institute for Oncology, Hematology, and Cell Therapy, Tehran University of Medical Sciences, Tehran 14117-13135, Iran; srad@sina.tums.ac.ir; 4 Cardiovascular Department, Rasoul Akram General Hospital Iran University of Medical Sciences, Tehran 14456-13131, Iran; hajahmadipour.m@iums.ac.ir

**Keywords:** acute leukemia, cardiovascular complications, cardiotoxicity, cardio-oncology

## Abstract

Background: Patients with acute leukemia (AL) are at heightened risk of cardiovascular complications due to both disease-related and treatment-related factors. These complications include heart failure, arrhythmias, myocardial infarction, and thromboembolic events which may significantly impact morbidity and mortality. Objective: To identify the risk factors contributing to cardiovascular complications in patients with acute leukemia. Methods: This systematic review was conducted according to the PRISMA reporting guideline. Multiple databases including PubMed, Scopus, IEEE Xplore, the Cochrane Library, Web of Science, ProQuest, and Google Scholar were searched for studies published between 2020 and 2024. Eligible studies included those analyzing cardiovascular risk factors in AL patients across various subtypes and treatment stages. A total of 75 studies were included following rigorous screening and critical appraisal using tools appropriate for different study designs. Results: The results showed that cardiovascular complications in AL patients are multifactorial including demographic factors (e.g., age, sex, BMI), comorbidities (e.g., hypertension, diabetes, dyslipidemia), treatment exposures (e.g., anthracyclines, tyrosine kinase inhibitors, hematopoietic stem cell transplantation, radiation), and genetic predispositions (e.g., somatic and germline variants). Cardiac biomarkers (e.g., troponins, BNP), imaging (strain echocardiography), and electrocardiogram (ECG) abnormalities were key factors in detecting early or subclinical damage. Complications occurred both during and years after treatment, especially in childhood and long-term survivors. Conclusions: Cardiovascular complications are prevalent, and serious consequences in AL patients necessitate a personalized, multidisciplinary approach to risk stratification and monitoring. Considering clinical, genetic, and biomarker data can improve early detection and preventive strategies, ultimately enhancing patient outcomes.

## Simple Summary

Patients with acute leukemia are at significant risk for cardiovascular complications such as heart failure, arrhythmias, myocardial infarction, and thromboembolic events, which adversely affected morbidity, quality of life, and survival. These complications arise from a complex interplay of factors, including the malignancy itself, existing cardiovascular conditions, and the cardiotoxic effects of intensive therapies like chemotherapy, radiotherapy, and stem cell transplantation. This study identified key clinical and treatment-related risk factors for cardiac adverse events in this population. Understanding these risks enabled earlier detection of cardiac injuries, safer selection of treatment modalities, and personalized monitoring for vulnerable patients. The findings supported the development of targeted prevention strategies and individualized cardio-oncology care, aiming to reduce cardiovascular morbidity related to leukemia treatment and improve both short- and long-term outcomes for patients.

## 1. Introduction

Acute leukemia (AL) is a heterogeneous group of hematologic malignancies characterized by the rapid proliferation of abnormal blood cells, leading to bone marrow failure and systemic complications [1]. While advancements in leukemia treatment, including chemotherapy, targeted therapies, and hematopoietic stem cell transplantation (HSCT) have significantly improved patient survival, the risk of cardiovascular complications remains a major concern. These complications impact survival and reduce quality of life, as patients face the dual challenge of managing their disease and treatment effects [2,3].

Patients with AL are particularly vulnerable to cardiovascular complications like acute thromboembolism (ATE), myocardial infarction, ischemic stroke, heart failure, and arrhythmias [4,5]. Cardiovascular complications are common and potentially fatal among leukemia patients with incidence rates reaching ~19.5% at 3 years and ~56.9% at 9 years in AML and 28% at 5 years without prior cardiovascular disease (CVD) history [4,6,7]. Several factors that contribute to this heightened risk include pre-existing cardiovascular risk factors, direct leukemic infiltration of cardiac tissues, endothelial dysfunction, and prothrombotic states induced by malignancy [2,8]. Chemotherapeutic agents, particularly anthracyclines, tyrosine kinase inhibitors (TKIs), and corticosteroids are associated with cardiotoxic effects that can manifest as acute or late-onset cardiovascular events [9,10]. Radiation therapy, especially in the chest area further compounds the risk by causing myocardial fibrosis and vascular damage [11].

The identification and evaluation of risk factors for cardiovascular complications in AL patients are critical for optimizing treatment strategies, improving long-term outcomes, and minimizing treatment-related adverse events while maintaining therapeutic efficacy [2]. Established risk factors include age, genetic predisposition, pre-existing CVD, diabetes, hypertension, dyslipidemia, obesity, and metabolic syndrome [2,12,13]. Existing guidelines often base monitoring of cardiotoxicity on general cancer populations and do not fully address the individual needs of leukemia patients, who frequently undergo intensive and prolonged treatment regimens. The considerable variability in individual treatment responses further accentuates the need for personalized approaches to cardiovascular risk stratification [14,15].

This review aims to determine the risk factors associated with cardiovascular complications in patients with AL. The findings can help to provide insights into risk stratification, early detection, and preventive strategies implementation. Understanding these risk factors will ultimately aid in developing tailored cardioprotective interventions to enhance patient outcomes while maintaining the efficacy of acute leukemia treatment.

## 2. Materials and Methods

### 2.1. Study Design, Information Sources and Search Strategy

This systematic review was performed in accordance with the PRISMA (Preferred Reporting Items for Systematic Reviews and Meta-Analyses) guidelines and has not been registered. The searched databases included PubMed, IEEE Xplore, Scopus, the Cochrane Library, Web of Knowledge, ProQuest, and Google Scholar. The period for searching articles was from the beginning of 2020 to the end of 2024. To search databases, a combination of keywords, their synonyms, and MeSH terms related to AL (“Acute Myeloid Leukemia” and “Acute Lymphoid Leukemia”) and heart disease risk factors (“Heart Disease Risk Factors”) was used to create search strings using the logical operators ‘AND’ and ‘OR’. Detailed database search strategies are provided in Supplementary S1.

To enhance the richness of search, references and citations of relevant articles were examined, focusing on those frequently cited or published in high-impact journals, as well as reviewing the scientific contributions of authors with multiple publications in the field to uncover additional studies.

### 2.2. Eligibility Criteria

The inclusion and exclusion criteria were as follows:

#### 2.2.1. Inclusion Criteria

1. Studies published from the beginning of 2020 to the end of 2024.
2. Theses, original studies, review articles, and conference papers with full text available.
3. Studies that investigated risk factors associated with the development of cardiovascular complications in patients with AL.
4. Studies that included different AL types, such as acute lymphoblastic leukemia (ALL) with B and T subtypes, acute myeloid leukemia (AML) with genetic variations, AML with dysplastic changes, treatment-related AML, and unspecified AML (such as minimally differentiated AML, AML without mutations, or with mutations), as well as specific types (acute myelomonocytic leukemia, monoblastic/monocytic leukemia, erythroleukemia, megakaryoblastic leukemia, and other rare cases); mixed-phenotype acute leukemia (MPAL) and acute leukemias with undefined lineage.
5. Studies that included quantitative or qualitative analyses regarding the risk complications leading to cardiovascular issues in patients with AL.
6. Studies that addressed cardiovascular complications related to bone marrow transplantation and treatments for AL, including complications arising from bone marrow transplants such as side effects of immunosuppressive drugs (e.g., cyclosporine, tacrolimus), direct complications related to the transplant process (e.g., cardiotoxicity from AL treatments or high doses prior to transplant), and late complications after transplantation (e.g., heart failure, arrhythmias, or coronary artery disease (CAD)). Additionally, complications from AL treatments such as chemotherapy with drugs like anthracyclines (e.g., doxorubicin, daunorubicin, idarubicin, mitoxantrone), alkylating agents (e.g., cyclophosphamide, busulfan, melphalan), antimetabolites (e.g., cytarabine, fludarabine, cladribine, clofarabine, azacitidine, decitabine), TKIs (e.g., sorafenib, quizartinib, imatinib, dasatinib, nilotinib, ponatinib), FLT3 inhibitors (e.g., midostaurin, gilteritinib), and isocitrate dehydrogenase (IDH) inhibitors (e.g., ivosidenib, enasidenib), monoclonal antibodies (e.g., rituximab, blinatumomab, gemtuzumab, ozogamicin, tisagenlecleucel), and drugs regulating apoptosis and cellular metabolism (e.g., venetoclax, etoposide, asparaginase) were considered. Other related treatments like radiation therapy and supportive treatments (e.g., colony-stimulating factors, blood transfusions, or infection prevention medications) were also included in this study.
7. The cardiac complications included direct heart problems such as heart failure, arrhythmias, CAD, myocarditis, pericarditis, cardiomyopathy, valvular heart disease, hypertension, cardiotoxicity, endocarditis, cardiogenic shock, and Takotsubo cardiomyopathy.
8. The vascular complications included issues with both large and small vessels such as deep vein thrombosis (DVT), pulmonary embolism (PE), stroke, peripheral artery disease (PAD), renal artery stenosis, aneurysm, vasculitis, pulmonary hypertension, electrolyte imbalances, and coagulopathies.

#### 2.2.2. Exclusion Criteria

1. Studies such as letters to the editor, protocols, preclinical studies, case reports, editorials, or opinions were excluded.
2. Articles that only had an abstract available and did not provide full-text access were excluded.
3. Articles that focused on other cancers such as solid cancers (e.g., breast cancer, lung cancer, prostate cancer, or colorectal cancer) or hematologic cancers unrelated to AL (e.g., lymphoma or multiple myeloma) were excluded.
4. Articles that focused on complications and diseases caused by AL other than cardiovascular complications (e.g., neurological, renal, hepatic complications, or treatment-related infections) were excluded.
5. Articles that discussed general chemotherapy, radiotherapy, or bone marrow transplant complications without a specific focus on AL or its related cardiovascular complications were excluded.
6. Studies that investigated complications or diseases related to AL, such as gastrointestinal, pulmonary, or ocular complications, but with no focus on cardiovascular complications were excluded.
7. Studies that explored general risk factors for cardiovascular complications but did not clearly analyze the impact of these factors in patients with AL were excluded.
8. Articles focused on topics unrelated to the risk factors for cardiovascular complications (e.g., alternative treatments or prevention) were excluded.
9. Studies that focused on clonal hematopoiesis and myeloproliferative neoplasms (MPNs), including chronic myeloid leukemia (CML), polycythemia vera (PV), essential thrombocythemia (ET), primary myelofibrosis (PMF), chronic neutrophilic leukemia (CNL), chronic eosinophilic leukemia/hypereosinophilic syndrome (CEL/HES), mast cell disease (MCD), myelodysplastic syndromes (MDS), myelodysplastic/myeloproliferative neoplasms (MDS/MPN), chronic myelomonocytic leukemia (CMML), juvenile myelomonocytic leukemia (JMML), and neoplasms related to gene rearrangements (PDGFRA, PDGFRB, FGFR1, and PCM1-JAK2), without addressing AL and its associated complications were excluded.

### 2.3. Selection Process

The screening process followed the guidelines outlined in the Preferred Reporting Items for Systematic Reviews and Meta-Analysis (PRISMA) checklist [16]. After retrieval of relevant articles, reference management was performed using EndNote software (Clarivate, version X21, Toronto, ON, Canada) to remove duplicates. Subsequently, titles, abstracts, and full texts of retrieved studies were comprehensively screened.

### 2.4. Data Collection Process and Data Items

A data extraction form was completed for all selected studies to collect the required data in a structured manner. This included author(s), year of publication, country of study, research methodology, research objective, type of AL, risk factors, type of cardiovascular complications, time of cardiovascular complications onset (before treatment, during treatment, early-onset, late-onset), and key findings.

Conducting the meta-analysis was not feasible in this study due to the heterogeneity observed across the included studies. The variability in outcome measures was significant, with some studies focusing on cardiovascular complications, severity, and timing of onset, while others emphasized risk factors or treatment conditions. The studies included different types of AL and diverse patient populations, which further contributed to the heterogeneity.

### 2.5. Study Risk of Bias Assessment

In this systematic review, the methodological quality and risk of bias of included studies were critically appraised using validated and widely accepted tools appropriate to the study design. Randomized controlled trials were assessed using the Joanna Briggs Institute (JBI) Critical Appraisal Checklist, (quality items: poor ≤ 0.40, moderate 0.41 to 0.59, good 0.60 to 0.79, excellent ≥ 0.80) [17] and the version 2 of the Cochrane risk-of-bias tool for randomized trials (RoB 2) tool [18]. Systematic reviews underwent evaluation with the Measurement Tool to Assess systematic Reviews (AMSTAR 2) [19] and Risk Of Bias In Systematic reviews (ROBIS) tools to determine methodological quality and risk of bias [20]. Narrative reviews were appraised using the Scale for the Assessment of Narrative Review Articles (SANRA) scale, (quality items: low < 7, moderate 7 to 9, high 10 to 12) [21]. Qualitative studies were evaluated with a triangulation checklist to ensure methodological rigor [22]. Genetic association studies were assessed with the Q-Genie checklist to identify common biases, (Poor quality 0–35, Moderate quality 36–45, Good quality 45–77) [23]. Observational studies, including cohort, (quality items: low < 0.6, moderate 0.6 to 0.8, high > 0.8) [24], case–control, (quality items: low < 0.6, moderate 0.6 to 0.8, high > 0.8) [25], case series, (quality items: low < 0.6, moderate 0.6 to 0.8, high > 0.8) [26], and cross-sectional designs, (quality items: low < 0.6, moderate 0.6 to 0.8, high > 0.8) [25], were critically appraised using respective JBI checklists and the Risk Of Bias In Non-randomized Studies-of Interventions (ROBINS-I) tool for non-randomized interventions [27]. Pre-post studies without control groups were evaluated using the NIH Quality Assessment Tool, (quality items: low < 0.6, moderate 0.6 to 0.8, high > 0.8) [28]. The details of all quality assessments were provided in Supplementary S2.

### 2.6. Synthesis Methods

Given substantial heterogeneity in study designs, populations, and outcome measures, a narrative synthesis was conducted. Eligible studies were summarized in tables, and the extracted data included the name of the country, research methodology, research objective, type of AL, risk factors, type of cardiovascular complications, time of cardiovascular complications onset (before treatment, during treatment, early-onset, late-onset), and key findings. Patterns, similarities, and differences across studies were analyzed to delineate consistent findings and knowledge gaps. Quality and risk of bias were assessed for each study using appropriate critical appraisal tools. Interpretation prioritized high-quality, low-risk studies, and findings from studies with high risk of bias were treated cautiously, noting their potential influence. Quantitative meta-analysis was precluded by variability in outcome definitions, follow-up periods, and measurement methods; thus, results were summarized descriptively.

## 3. Results

### 3.1. Study Selection

**Figure 1:**
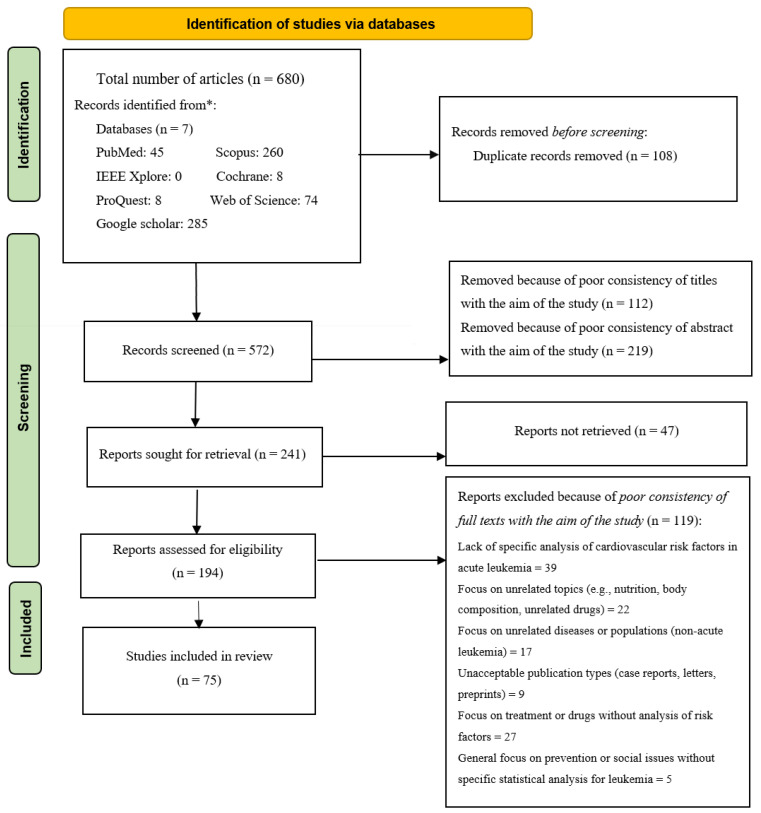
PRISMA-based article selection process.

This PRISMA 2020 flow diagram, which is shown in Figure 1, illustrates the systematic review process for 680 identified articles. After removing 108 duplicates, 572 records were screened, and 331 were excluded based on title or abstract relevance. Out of 241 reports sought, 47 were not retrieved, and 119 full texts were excluded due to poor alignment with the study aim. The main exclusion reasons included a lack of analysis on cardiovascular risk in AL and unrelated topics or diseases. Ultimately, 75 studies met the eligibility criteria and were included in the final review.

### 3.2. Study Characteristics

**Figure 2:**
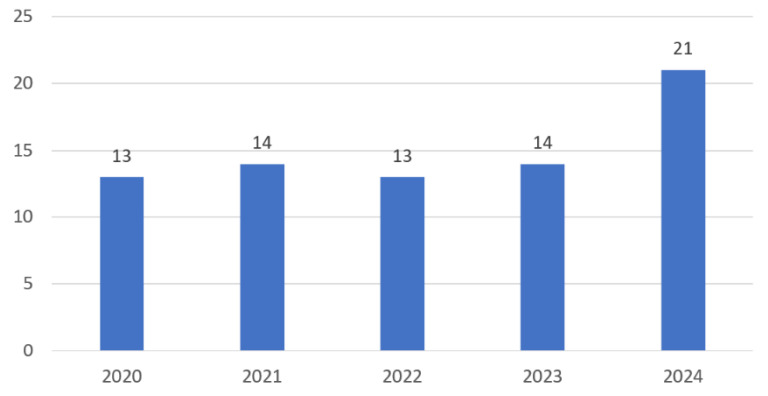
Frequency distribution of the studies by year of publication.

As noted previously, the included studies were published between 2020 and 2024. The frequency distribution of these publications across the years is shown in Figure 2.

**Figure 3:**
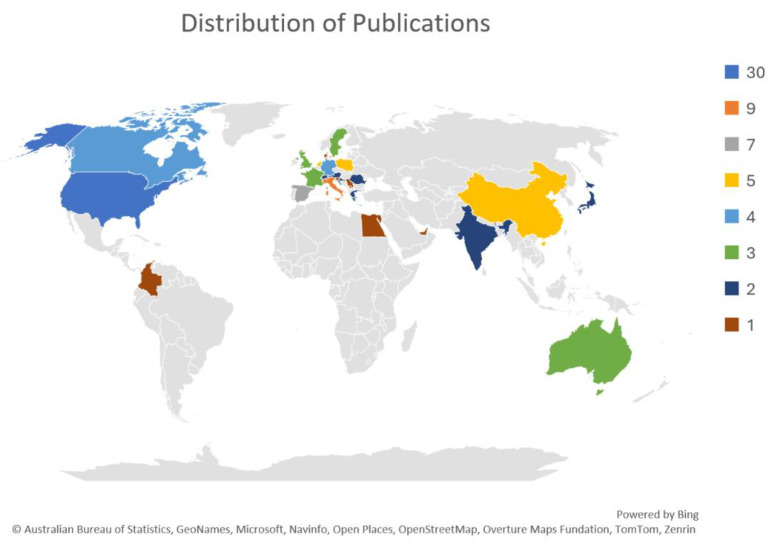
Geographical distribution of the studies.

In terms of geographical distribution, the majority of the studies originated from the United States of America (*n* = 30), followed by Italy (*n* = 9) and Spain (*n* = 7). Other countries contributed to publish relevant papers included the Netherlands, Poland, and China (each with 5 studies); Germany and Canada (each with four studies); and Australia, the United Kingdom, France, and Sweden (each with three studies). Additional contributions came from Austria, India, Greece, Croatia, Japan, and Switzerland (each with two studies), and single studies were from Romania, the United Arab Emirates, Slovenia, Colombia, Egypt, Serbia, Denmark, and Kosovo. Figure 3 illustrates the geographical distribution of the relevant papers.

### 3.3. Risk of Bias in Studies

The quality of the included studies was assessed using specific tools. Overall, the quality scores varied across studies but demonstrated an acceptable standard supporting the reliability of the synthesized evidence. Randomized controlled trials (RCTs) showed a broad quality score ranged from poor to excellent (0.30–0.84). Cohort studies generally presented moderate to high quality, with typical scores between 0.63 and 0.90. Case–control studies exhibited moderate to high quality, and their score was between 0.70 and 1.0. Cross-sectional studies were consistently rated as high quality, with scores between 0.87 and 1.0, reflecting a robust methodology. Case series and genetic association studies showed moderate to good quality, with the best scores of 0.7 and 0.9, respectively. Narrative reviews also demonstrated high quality, scoring between 10 and 12 on the SANRA scale. Systematic reviews mostly fell within moderate quality (0.31–0.75), indicating some methodological limitations, but still contributing valuable insights. Pre-post studies without control groups were rated as low quality (0.58), reflecting inherent design constraints (Supplementary S2).

In addition to the quality assessment, the risk of bias was examined using validated tools. Among the five randomized controlled trials assessed using the RoB 2.0 tool, three had low risk of bias and two showed some concerns but were not at high-risk. Two systematic reviews were evaluated by the ROBIS tool revealed some concerns regarding bias, suggesting that their conclusions should be interpreted carefully due to potential methodological or reporting limitations. The single genetic association study was rated as low risk of bias, reflecting a robust design and analysis. Twenty-eight qualitative studies showed a split between low risk in 13 studies and moderate risk in 15 studies, primarily due to the limitations in methodological transparency and data triangulation, with no studies deemed high risk. Among the 48 non-randomized intervention studies assessed using ROBINS-I, 15 exhibited low and 33 showed moderate risk of bias. The moderate ratings highlight the inherent challenges of non-randomized designs, such as confounding and selection bias, despite generally sound methodology (Supplementary S2).

### 3.4. Results of Individual Studies

Table 1 provides a summary of the studies included in this systematic review.

#### 3.4.1. Research Methodology

Quantitative studies constituted the majority of the reviewed articles [2,4,7,13,29,30,33,34,36,37,38,40,41,42,44,45,47,48,50,51,52,53,54,55,59,60,61,63,64,66,68,69,73,74,75,77,80,81,83,86,87,89,90,96,99], and included clinical trials, experimental designs, observational studies, and other quantitative approaches. These research designs enable statistical analysis, hypothesis testing, and the identification of correlations or causal relationships through controlled variables and measurable outcomes. Review studies formed a moderate proportion of the included studies [32,35,46,49,56,58,62,65,67,70,71,72,76,78,79,82,84,85,88,91,93,94,95,97,98], and offered critical synthesis and evaluation of the existing evidence. Qualitative studies [31,39,43,57,92] including retrospective case series, represented a small share, which contributed to provide an in-depth insight into complex phenomena through non-statistical analysis.

#### 3.4.2. Research Objectives

The main objective of several papers was related to cardiotoxicity and its incidence, risk factors, and prevention. These studies primarily investigated the incidence, risk factors, and prevention of chemotherapy-induced cardiotoxicity, with a particular emphasis on anthracycline-related effects. Research in this group also explored the use of biomarkers and management strategies to mitigate cardiac injury in patients with hematologic malignancies [4,7,29,30,32,33,34,36,37,38,39,48,49,52,58,61,62,63,64,65,66,69,70,71,73,76,77,78,86,91,94,95,98].

CVDs and its risk factors in survivors were related to the long-term cardiovascular risks faced by survivors of childhood and adult hematologic cancers. Key topics included the development of metabolic syndrome, late cardiac effects, and the need for ongoing surveillance and preventive care in these populations [2,31,35,42,43,44,45,50,51,55,59,74,75,80,82,83,87,90,92]. Some studies focused on the incidence, risk factors, and management of arterial and venous thromboembolic events as well as other vascular complications in patients with hematologic malignancies. The studies investigated both the clinical burden and strategies for prevention and intervention [13,40,41,53,56,57,60,89,96].

Several studies investigated the genetic and molecular underpinnings of cardiotoxicity, including the identification of risk-modifying genetic variants, biomarkers, and mechanistic pathways that predispose patients to cardiovascular complications during and after cancer therapy [7,51,52,63,70,73,98]. Studies with a focus on cardiac imaging, function, and monitoring utilized advanced imaging modalities, such as echocardiography and strain imaging to assess cardiac function before, during, and after treatment and showed the importance of early detection and monitoring of cardiac dysfunction in at-risk patients [36,44,45,55,58,69,74].

Another important objective of the studies was cardiotoxicity and cardiovascular complications associated with newer cancer therapies, including tyrosine kinase inhibitors, CAR-T cell therapy, and other novel agents. These studies examined both the mechanisms of toxicity and clinical management strategies [30,54,62,72,84,85,88,93,95,97]. Cardiac complications and outcomes related to HSCT were also addressed in some studies and included the impact of conditioning regimens and the importance of long-term cardiac follow-up in transplant recipients [34,36,46,50,60,79,81,82,96]. Studies also provided recommendations and guidelines for the prevention, surveillance, and management of cardiovascular complications in patients with hematologic malignancies. Structured follow-up and evidence-based strategies to reduce cardiac risk were also emphasized [35,49,56,59,71,78,92,94].

Some studies focused on research examining specific risk factors for CVD, such as sex differences, age, frailty, and metabolic syndrome in patients with hematologic cancers. These studies aimed to identify vulnerable subgroups and tailor interventions accordingly [43,47,50,68]. Studies with unique or cross-cutting objectives that were not easily categorized within the other groups were also included. These focused on atypical presentations and broader management considerations of the risk factors associated with cardiovascular complications in patients with AL [67,99].

#### 3.4.3. Type of Acute Leukemia

The included studies addressed various subtypes of AL, including ALL [31,33,43,44,45,55,58,61,62,63,66,67,68,69,73,76,77,79,82,84,85,89,90,93,94,97], which primarily affects children and young adults, and AML [4,7,30,34,37,38,53,70,71,72,78,87,91,95], which is more common in adults and associated with a poorer prognosis. A specific subtype of AML, i.e., Acute Promyelocytic Leukemia (APL) [13,56], was also investigated due to its unique treatment responses and coagulopathy risks. Some studies focused on Philadelphia Chromosome Positive ALL (Ph+ ALL) [54,57,62,88], a genetically distinct and high-risk subtype. Others examined mixed AL cases which included overlap between ALL and AML or combinations that included APL [2,13,29,32,35,36,39,40,41,42,46,47,48,49,50,51,52,59,60,64,65,74,75,80,81,86,96,98,99]. Additionally, several articles referred to general or unspecified AL. These studies discussed broader patterns and complications without limiting their focus to a specific subtype [83,92].

#### 3.4.4. Risk Factors

Demographic variables and anthropometric measures were key risk factors for cardiovascular complications in patients with AL. Age was mentioned in various contexts (at diagnosis, first anthracycline dose prescription, beginning of treatment, etc.) and was frequently studied due to its influence on treatment responses and long-term cardiovascular risk [2,4,7,13,32,34,35,37,38,40,41,42,44,45,47,48,50,51,52,53,54,55,56,57,58,59,60,61,63,64,66,67,68,69,71,73,74,75,76,77,79,80,81,83,85,86,87,89,90,91,92,95,96,98,99]. Sex was another reported variable, with studies showing that female patients were more susceptible to cardiotoxicity, possibly due to hormonal and physiological differences [4,7,13,32,37,38,42,44,45,47,48,50,51,52,53,55,58,59,60,61,64,67,68,69,71,73,74,75,77,80,81,86,87,89,90,91,92,98,99]. Race/ethnicity helped to identify disparities in health risks [4,42,56,60,68,75,80,99]. Among anthropometrics, BMI was the most cited, linking obesity to cardiovascular issues; other measures like weight, height, waist circumference, and body composition offered additional insight into metabolic health and treatment effects [7,13,29,33,37,41,44,45,47,50,52,53,55,59,60,66,68,69,75,83,86,89,90,96].

Medical history and comorbidities played a major role in identifying cardiovascular risk in patients with AL. The most frequently reported factors included comorbidities (e.g., diabetes, hyperlipidemia, obesity, dyslipidemia, chronic kidney disease, pulmonary risks, endocrine complications, hypothyroidism, insulin resistance, frailty, metabolic disorders, abnormal renal or liver function, trisomy 21, celiac disease, asthma, periodic fever) [2,4,7,13,30,35,37,38,40,41,43,44,46,51,53,54,55,56,60,61,63,64,68,69,71,74,75,78,80,81,83,85,86,88,89,91,92,95,97,99], Hypertension [2,7,13,30,37,41,43,44,46,51,52,53,54,55,56,60,62,69,71,74,75,78,80,81,82,83,88,95,97], pre-existing cardiovascular conditions [4,7,32,34,35,36,37,49,53,56,57,60,62,64,76,78,80,81,83,85,88,91,93,95,98], and existing CVDs [7,30,31,46,56,60,61,62,63,64,65,75,78,79,84,85,88,90,91,93,94], all of them were strongly linked to increased cardiotoxicity. Smoking history was a key modifiable risk factor [2,7,13,29,35,38,41,44,52,53,55,60,68,69,71,74,75,80,81,83,88,89,96,97,99], while blood pressure levels helped to detect early cardiovascular strain [29,44,50,55,59,66,67,68,69,83,88,90,96]. Prior exposure to cardiotoxic cancer treatments further raised risk [30,31,41,44,49,62,63,64,75,96]. Though mentioned less often, psychological and mental health factors [75,82] and lifestyle factors, such as physical activity, sedentarism, and alcohol use also contributed to overall cardiovascular health [13,35,41,44,48,55,68,69,71,72,75,83,91,99].

Cancer and treatment-related factors were central to understanding cardiovascular complications in patients with AL. The type and intensity of cancer therapy, especially cancer treatment exposures, were frequently investigated due to their known long-term cardiotoxic effects [2,4,7,13,29,32,34,35,36,40,41,42,43,47,48,50,51,52,53,55,57,58,61,62,63,64,65,67,68,71,74,75,76,78,79,80,81,86,87,88,89,91,92,94,96,98,99]. Pharmacological cancer treatments, necessary medications, and cumulative doses were other most frequently cited factors, given their strong association with both early and late cardiac events [4,7,13,37,41,46,47,48,49,51,53,55,56,57,58,61,62,63,64,65,67,68,69,70,71,72,73,74,76,78,79,80,81,85,86,87,88,89,91,92,94,95,97,98]. Although less frequently mentioned, time-related variables (e.g., time since diagnosis, duration of therapy, and time from treatment initiation to echocardiography) helped to monitor for delayed cardiac events [34,37,44,48,55,57,58,59,63,64,83,86,90]. Long-term complications of AL treatment could affect multiple organs, lead to long-term chronic sequelae and Complications [82,87,94]. Cytogenetic abnormalities, cytokine release syndrome (CRS)-related inflammation, treatment response parameters, and genetic variants collectively contributed to cardiovascular complications in AL by influencing inflammatory burden, therapy response, and drug metabolism [4,38,49,53,54,84,85,88,91,93,97].

Laboratory and biomarker parameters were crucial in assessing cardiovascular risk in patients with AL. Among the most frequently investigated factors, cardiac biomarkers, such as troponins and NT-proBNP were highlighted due to their strong association with treatment-related cardiotoxicity [2,29,30,38,39,52,69,70,72,74,77,84,85,86,94]. Blood count parameters [2,4,13,38,41,45,52,53,54,73,74,89] and genetic factors [7,31,35,38,43,51,54,62,63,64,70,71,73,76,95] were also commonly evaluated, reflecting disease burden and treatment decisions that can impact cardiac outcomes. Lipid profiles [50,52,59,66,68,69,83,88,90,96], metabolic parameters [2,4,50,52,53,66,68,69,83,88,96] and coagulation profile [2,4,52,53,89] were frequently included because they link metabolic and vascular health. Other notable markers included renal function [2,4,45,52,74,88], cell biomarkers [4,29], biochemical parameters [45,88], hepatic function [2,4,52], endothelial dysfunction [33,66], serum proteins [31], molecular factors [4,31,36,72], inflammatory markers [33,36,70,94], cancer biomarkers [70], cellular biomarkers [96] and other biomarkers [70] which contributed to the complex interplay between leukemia, its treatment, and cardiovascular complications.

Cardiac and vascular assessments were essential approaches for monitoring cardiovascular complications in patients with AL, with echocardiographic parameters being the most frequently used due to their ability to detect both structural and functional cardiac abnormalities [2,7,29,36,37,38,39,44,45,48,52,55,61,66,69,71,72,73,74,77,86]. Heart rate was also commonly assessed, reflecting its utility as a simple yet informative marker of autonomic imbalance and cardiovascular stress [33,44,55,57,67,69,74,96]. Electrocardiographic parameters were investigated for their relevance in detecting early electrical disturbances related to leukemia therapies [4,39,58,67,88]. Less frequently, tests such as cardiovascular risk factor screening and assessing [47,57,59,82], autonomic function testing [34], myocardial morphology [29], transthoracic echocardiogram [72], vascular ultrasound parameters [66], and CTCAE grading [34,97] were used to assess overall cardiac health and the severity of adverse events.

Other clinical and treatment-related factors in leukemia and hematopoietic cell transplantation encompassed a wide range of variables influencing patient outcomes, disease progression, and complications included prophylaxis type (post-transplant cyclophosphamide-based versus other graft-versus-host disease (GVHD) prophylaxis), risk stratification groups, AML subtypes such as therapy-related AML (t-AML) with its poorer prognosis, bone marrow blast ratio, disseminated intravascular coagulation, thromboembolism, antiplatelet therapy, central nervous system (CNS) involvement, vaccination management, transplant procedure parameters (donor type, stem cell source), disease status at transplantation, pharmacokinetic interactions affecting drug safety and efficacy, as well as socioeconomic status, health insurance, and technology access impacting treatment adherence and outcomes [2,7,13,34,35,38,40,41,42,45,46,47,49,50,52,53,54,55,58,59,60,61,63,64,67,69,71,72,73,75,76,77,78,79,80,81,82,83,84,85,87,88,89,90,94,95,96,97,99].

#### 3.4.5. Cardiovascular Complications

Coronary artery and ischemic heart diseases were among the most critical cardiovascular complications reviewed in the articles. Other topics included CAD, myocardial infarction, ischemic heart disease, acute coronary syndrome (ACS), myocardial ischemia, dysfunction and injury, and coronary dissection along with atherosclerotic cardiovascular disease (ASCVD), acute onset events (AOEs), unstable angina, transient ischemic attack (TIA), coronary heart disease and stroke [2,4,7,13,29,32,34,35,36,37,39,41,42,43,45,49,53,54,60,62,63,64,65,66,67,68,70,71,75,77,79,80,81,82,85,87,88,91,92,93,94,95,99].

Heart failure and ventricular dysfunction were other significant cardiac complications encompassing a range of conditions. These included various types of heart failure (HFrEF, HFmrEF, HFpEF), left and right ventricular dysfunctions, dilated cardiomyopathy, and cardiomyopathies related to anthracyclines or subclinical presentations. Additionally, the articles reviewed electromechanical dysfunction, cardiac injury, cardiac enzyme abnormalities, systolic and diastolic dysfunction, decreased and subclinical decline in left ventricular ejection fraction (LVEF), ventricular dysfunction, right ventricular systolic dysfunction, tricuspid annular plane systolic excursion (TAPSE) reduction, echo changes, diastolic dysfunction, LA stiffness, decompensated cardiac failure, cardiogenic shock, depressed LV function, decreased right ventricular four-chamber strain, and subclinical systolic and diastolic dysfunction [2,4,7,29,31,32,34,35,36,37,38,39,42,43,44,45,46,47,48,49,51,52,53,55,60,61,62,63,64,65,66,69,70,71,72,73,74,75,76,77,78,79,80,81,82,84,85,86,87,91,92,93,94,95,98,99].

Arrhythmias and conduction disorders were key cardiac complications investigated by studies, including atrial fibrillation (AF), various ventricular arrhythmias (VAs) (e.g., tachycardia, fibrillation, premature contractions), and bradycardia. The main issues included QT/QTc prolongation, heart blocks, heart rate abnormalities, cardiac repolarization and depolarization disturbances [4,7,29,32,34,35,36,37,39,42,43,49,52,53,56,57,58,60,61,63,64,65,67,70,72,75,76,78,80,81,82,84,85,86,91,92,93,94,95,97].

Valvular heart diseases referred to abnormalities in valve structure or function, including moderate valvulopathy, valvular insufficiency, and structural valve defects, which can significantly affect cardiac performance, hemodynamics and tricuspid regurgitation [29,34,35,43,63,71,86,92]. Pericardial diseases consisted of inflammatory and fluid-related conditions of the pericardium, including pericarditis, pericardial effusion, cardiac tamponade, and hemopericardium, which can impair cardiac function and require prompt diagnosis and management [4,7,29,34,35,43,49,60,61,64,65,67,76,78,94,99].

Hypertension and related vascular disorders included systemic and pulmonary arterial hypertension, hypotension, severe atherosclerosis, and endothelial dysfunction [4,7,13,29,33,36,39,42,43,46,49,50,53,54,59,61,62,65,66,68,69,70,71,72,75,78,79,80,82,83,84,85,86,87,88,89,90,91,92,93,94,95,96,97]. Associated conditions such as dyslipidemia, cardiometabolic risk factors (e.g., obesity, metabolic syndrome), vascular toxicity or occlusive events, arterial diseases, PAD and higher arterial stiffness contributed to increased cardiovascular risk [4,7,13,29,33,36,39,42,43,46,49,50,53,54,59,61,62,65,66,68,69,70,71,72,75,78,79,80,82,83,84,85,86,87,88,89,90,91,92,93,94,95,96,97].

Thromboembolic and vascular events involved both venous and arterial complications. Venous thromboembolism (VTE) included DVT, PE, renal vein thrombosis, and cerebral venous thrombosis; Arterial thromboembolism (ATE) covered ischemic stroke, acute lower extremity arterial thrombosis, thromboembolic stroke, PAD, and intracardiac thrombosis [7,32,40,41,42,49,53,54,61,62,63,65,66,67,71,78,79,80,81,88,89,94,95,96].

Inflammatory and infectious cardiac conditions included myocarditis, cardiotoxicity often induced by chemotherapy or cytokine release syndrome, and myocardial injury caused by infections or toxic exposures, leading to significant cardiac dysfunction [2,32,38,46,49,52,53,73,76,77,78,91,93,94,99]. Cardiac arrest and sudden cardiac death were also critical, life-threatening events that occur abruptly and often without warning, typically resulting from severe arrhythmias or underlying heart conditions [30,36,63,84,85,94].

**Figure 4:**
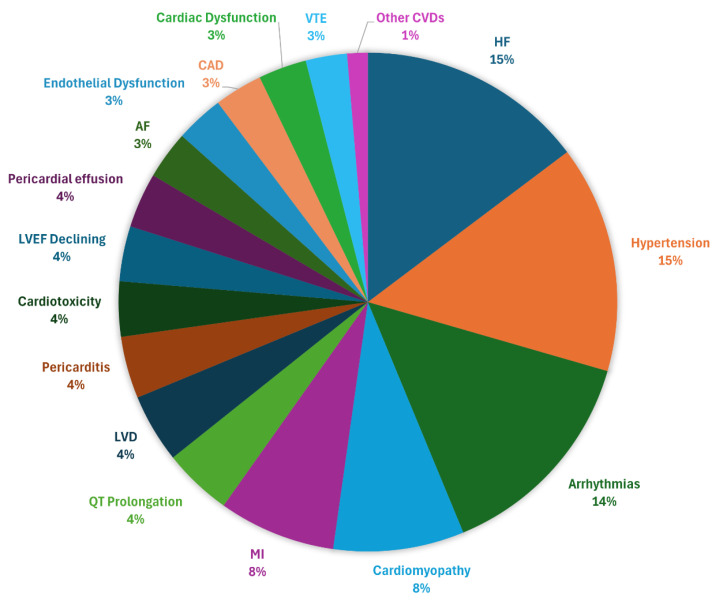
Distribution of the most frequent cardiovascular complications.

Other cardiac conditions encompassed a diverse range of abnormalities and complications, including cardiovascular autonomic dysfunction, structural defects, cardiac-related lesions, and other unclassified alterations. Findings such as increased left atrial stiffness, reduced LAS values, decreased right ventricular strain, cardiomyocyte damage, systolic and diastolic impairment, and cardiac masses (including leukemic infiltration) were also mentioned [33,38,42,44,51,58,64,66,67]. Additional concerns included pulmonary edema, impulse generation disturbances, platelet dysfunction, superior mesenteric artery occlusion, capillary leak syndrome, elevated Hs-cTnI, and increased cardiovascular risk in adulthood, even in the absence of traditional risk factors [63,69,85,86,90,93,96,97]. Figure 4 represents the distribution of the most frequent cardiovascular complications reported across 75 studies.

#### 3.4.6. Time of Cardiovascular Complication Onset

The time of cardiovascular complication onset was examined at various stages throughout the continuum of cancer care. Some articles reported complications before treatment [2,38], while others highlighted them as early-onset events [30,33,34,45,47,74,77,81,84,86], typically within days to weeks of therapy initiation. Several studies tracked cardiovascular issues from “during treatment” to “early-onset” [4,53,63,72,85,88,89,93] or from “during treatment” to “late-onset” [7,32,40,42,49,54,56,61,62,64,65,67,70,76,78,82,91,94,95,97], capturing progression over time. A subset explored complications which were developed from early to late-onset [29,46,57,58,68,71,79,82,98,99], reflecting long-term risk. Late-onset complications [31,35,43,44,48,50,51,55,59,60,66,69,73,75,83,87,90,92,96] were also analyzed, often years after therapy. Additionally, some studies focused on events which occurred strictly “during treatment” [52], or “during treatment and early-onset periods” [36,37], while others spanned phases, such as “before treatment to early-onset” [13,41], “before treatment to late-onset” [39], highlighting the dynamic timeline of cardiovascular risk in patients with hematologic malignancies.

#### 3.4.7. Summary of Key Findings

The findings indicated that substantial cardiac damage may occur in leukemia patients, which can be characterized by increased levels of biomarkers, such as BNP and troponin. Leukemia treatments can also worsen cardiac injury, with genetic and metabolic factors contributing to the severity of the condition [2,7,29,38,45,48,49,51,52,61,64,67,69,70,74,77,85,86,91,92,93,94]. Besides, the findings showed the prevalence of cardiovascular events and associated risk factors in patients with leukemia. These individuals are at heightened risk for thrombosis, stroke, and acute coronary events, which are further aggravated by chemotherapy, obesity, and a history of CVD. Additional factors such as older age, hypertension, and intensive treatment regimens significantly increase the likelihood of complications and mortality [4,13,37,40,41,42,47,50,53,55,59,60,63,68,75,78,80,82,83,84,85,87,88,89,99].

Several studies demonstrated drug-induced cardiotoxicity, revealing that chemotherapies, such as anthracyclines, TKIs, and arsenic trioxide, can lead to QT prolongation, arrhythmia, and heart failure. The risk depends on the specific drug, dosage, and underlying health conditions, underscoring the need for careful ECG and electrolyte monitoring. In some cases, cardiotoxic effects might be reversible upon discontinuation of the treatment. Cardioprotective strategies mitigate anthracycline-induced toxicity. Approaches such as dose adjustments and early intervention for cardiotoxicity and cytokine release syndrome enhance patient safety without compromising treatment efficacy [4,30,31,32,37,39,45,47,48,49,51,52,54,56,57,59,62,63,64,65,66,69,71,72,73,76,77,78,81,84,85,86,88,91,92,94,95,97,98].

Some findings focused on the importance of diagnostic tools and monitoring strategies. Advanced imaging techniques, such as echocardiography and global longitudinal strain (GLS), along with cardiac biomarkers, can detect subclinical cardiac dysfunction earlier than conventional methods. Strain imaging, in particular, is more sensitive than LVEF in identifying anthracycline-induced cardiac damage, allowing for earlier and more effective intervention [31,36,44,45,48,52,55,58,61,69,71,72,74,86,90,94,97].

Some studies investigated cardiovascular complications in pediatric and long-term survivors and showed that childhood leukemia survivors exhibit late cardiovascular effects, including hypertension, endothelial dysfunction, and heart failure. Anthracyclines and radiation were major contributors to these long-term risks, necessitating lifelong monitoring and preventive care [33,35,43,44,46,49,50,55,58,59,60,66,68,69,71,75,77,78,79,80,82,83,85,87,90,92,96].

The cardiovascular impact of HSCT and its conditioning regimens has also been extensively studied. HSCT survivors may face increased cardiovascular risks, largely attributed to treatments such as total body irradiation (TBI) and high-dose cyclophosphamide. Emerging alternatives like total marrow irradiation (TMI) may reduce some toxicities; however, late-onset cardiac complications remain a significant concern [34,36,46,47,50,54,55,59,60,65,69,74,78,79,80,81,82,87,96].

### 3.5. Synthesis of the Results

Based on the findings, cardiovascular complications in AL arise from a dynamic toxicity which might be due to the treatment-related factors (anthracyclines, TKIs, HSCT/TBI), patient-specific vulnerabilities (age, female sex, APL subtype, cardiometabolic comorbidities), and acute cytokine-release cardiotoxicity during and after treatment. In addition, sensitive biomarkers (hs-TnT, NT-proBNP) combined with advanced imaging provide an unparalleled window for detecting subclinical myocardial injury prior to ejection fraction decline. Therefore, the stratification of risk factors remains essential for early intervention and tailoring treatment protocols. This can help to implement life-saving strategies, such as tiered cardiac protection, time-sensitive monitoring, and risk-adapted long-term surveillance monitoring protocols.

## 4. Discussion

Cardiovascular complications present a substantial clinical challenge in managing patients with AL, where both disease-related factors and treatment modalities contribute to elevating the risk of cardiovascular diseases. This systematic review presents the current evidence regarding the risk factors associated with cardiovascular complications in patients with AL. The multifactorial etiology of cardiovascular morbidity in this type of patient included patient-related, disease-related, and treatment-related factors [2,4,7,13,29,30,31,32,33,34,35,36,37,38,39,40,41,42,43,44,45,46,47,48,49,50,51,52,53,54,55,56,57,58,59,60,61,62,63,64,65,66,67,68,69,70,71,72,73,74,75,76,77,78,79,80,81,82,83,84,85,86,87,88,89,90,91,92,93,94,95,96,97,98,99]. Moreover, cardiovascular complications in AL patients result from a complex interplay between pre-existing comorbidities, the unique pathophysiology of leukemia, and the cardiotoxic effects of intensive therapies [2,4,7,13,29,30,31,32,33,34,35,36,37,38,39,40,41,42,43,44,45,46,47,48,49,50,51,52,53,54,55,56,57,58,59,60,61,62,63,64,65,66,67,68,69,70,71,72,73,74,75,76,77,78,79,80,81,82,83,84,85,86,87,88,89,90,91,92,93,94,95,96,97,98,99].

According to the literature, the most important risk factors included age, sex, comorbidities, pharmacological cancer treatments, cancer treatment exposures, pre-existing hypertension, history of smoking, pre-existing cardiovascular conditions, BMI, echocardiographic parameters abnormalities, and cardiac biomarkers. In addition, the most frequently reported cardiovascular complications in patients with AL included HF and hypertension, followed closely by arrhythmia.

The results of the current study are consistent with prior oncology-related literature, in which traditional cardiovascular risk factors such as older age, history of smoking, BMI, female sex, hypertension, dyslipidemia, diabetes mellitus, and obesity emerged as significant predictors of adverse cardiovascular outcomes [100,101]. Some studies reported a higher risk of cardiotoxicity in females, which may be related to differences in medication metabolism, body composition, and hormonal influences, although other findings are not entirely consistent and require further study [102,103,104].

The results showed that treatment intensity and modality, particularly pharmacological cancer treatments and specific therapeutic exposures were significant determinants of cardiovascular complications in acute leukemia patients. Anthracyclines remain a cornerstone in the treatment of many acute leukemias due to their potent antineoplastic effect. However, their cardiotoxicity is well-documented and dose dependent [105]. Biologically, anthracyclines induce oxidative stress, mitochondrial dysfunction, and direct cardiomyocyte injury, leading to progressive myocardial cell death and fibrosis [106]. The resulting cardiomyopathy often manifests as dilated cardiomyopathy and heart failure which was mentioned by other researchers. [105,107,108]. Clinically, this damage is frequently irreversible because of limited myocardial regenerative capacity, making cumulative dose monitoring essential. HSCT regimens add further cardiovascular insult by causing endothelial injury, microvascular damage, and promoting inflammatory and fibrotic remodeling in cardiac tissue, thereby exacerbating the risk of both acute and chronic cardiac dysfunction in transplant recipients [109]. TKIs have revolutionized targeted therapy in leukemia, especially in Ph+ ALL and some AML subtypes [110]. However, many TKIs, particularly Ponatinib, have off-target vascular toxicities. Ponatinib notably increases the risk of arterial thromboembolic events and induces hypertension, likely due to endothelial dysfunction, proinflammatory cytokine activation, and direct vascular toxicity [111,112].

Similar to the results of the current study, other studies showed that radiation therapy to the mediastinum [113], HSCT [109], and high-dose cyclophosphamide-based conditioning regimens [114] are major contributors to long-term cardiovascular risk in cancer survivors, particularly those treated in childhood or young adulthood. HSCT can lead to late-onset cardiac complications, including heart failure and arrhythmias, caused by total body irradiation, intensive conditioning, and GVHD, with post-transplant GVHD and prolonged immunosuppression further worsening myocardial injury and chronic cardiovascular dysfunction [109,113,115,116,117]. Despite advances like total marrow irradiation and pharmacologic prophylaxis, late cardiovascular effects persist. Treatment-induced cardiac and vascular damage, leading to fibrosis, accelerated atherosclerosis, and dysfunction is especially pronounced in children and young adults, increasing long-term risks of heart failure, CAD, and other complications. The synergistic effects of multimodal therapy further amplify cardiovascular risk and toxicity, necessitating vigilant monitoring and early intervention [118,119].

Pre-existing and existing cardiovascular disease conditions were also identified as significant risk factors in patients with AL. These included arrhythmia, CAD, HF, valvular disease, myocardial ischemia, ischemic heart disease, VTE, acute cardiac events before HSCT, and structural heart abnormalities. Collectively, these conditions predispose patients to higher rates of acute and long-term cardiovascular complications during and after leukemia treatment [12,120].

Different types of AL exhibit distinct cardiovascular profiles that differ in terms of onset, underlying mechanisms, and long-term outcomes [121]. ALL typically presents cardiovascular complications that emerge during intensive chemotherapy phases, particularly from anthracycline-induced cardiotoxicity, which can manifest as acute heart failure or subclinical left ventricular dysfunction that may not become apparent until years or decades later [122]. By contrast, AML often presents cardiovascular complications at diagnosis due to pre-existing comorbidities and the acute effects of leukostasis, tumor lysis syndrome, and coagulopathy, leading to immediate risks of arrhythmia, thromboembolism, and bleeding complications [92,123]. ALL survivors face prolonged risks of cardiomyopathy, accelerated atherosclerosis, and secondary cardiovascular malignancies from extended survival and cumulative treatment, while AML patients, who are typically older and undergoing intensive therapy, experience acute cardiovascular morbidity. These distinct profiles necessitate tailored strategies, lifelong surveillance for ALL and intensive peritreatment cardiovascular support with risk stratification for AML [38].

Complementing imaging, biomarkers including troponins and natriuretic peptides (e.g., NT-proBNP) have demonstrated significant utility in detecting early cardiotoxicity. These are consistent with the results of other studies [124,125]. Despite their potential, these tools are underutilized in clinical practice due to inconsistent sensitivity and specificity, and the lack of standard diagnostic thresholds [126,127,128]. Integrating genetic, biomarker, and imaging data can enable personalized risk prediction for cardiotoxicity; Skitch et al. demonstrated that combining genetic variants, circulating biomarkers, and cardiac imaging can improve early detection of anthracycline-induced cardiotoxicity [129].

Genetic and molecular factors significantly modulate cardiovascular risk in AL patients. Mutations, such as DNMT3A, TP53, ASXL1, and IDH1/2 frequently are observed in AML, and have been linked to increased incidences of heart failure, coronary artery disease, and thromboembolic events [130,131,132]. These mutations may heighten myocardial vulnerability to chemotherapy-induced injury through mechanisms involving altered cellular metabolism, inflammatory signaling, and impaired repair pathways. Clonal hematopoiesis of indeterminate potential (CHIP) links hematologic mutations directly to cardiovascular morbidity, highlighting a shared pathophysiology between leukemogenesis and CVD [7,133,134]. Beyond somatic mutations, germline polymorphisms in genes involved in drug metabolism and oxidative stress (e.g., carbonyl reductase, NAD(P)H oxidase, TOP2B) increase susceptibility to anthracycline-induced cardiotoxicity [135,136,137,138,139].Though still evolving, pharmacogenomics represents a promising avenue for risk stratification and the personalization of therapy in AL patients.

Echocardiographic parameters are also essential for early detection of cardiovascular complications in AL. Measures such as LVEF, left ventricular dimensions, diastolic function, and strain imaging, including GLS, can reveal subclinical myocardial injury before overt heart failure. These changes may result from treatment-related cardiotoxicity or disease-related cardiac stress [3,69,140,141]. Clinical experience shows that the mentioned risk factors, significantly reduce cardiac reserve, thereby increasing the vulnerability of patients to cardiotoxic effects of cancer therapies [3,12]. The diminished cardiac reserve means that the heart has limited capacity to compensate for additional stress or injury caused by these treatments, which can lead to early onset or exaggerated cardiac dysfunction [142]. These comorbidities impair vascular function, reduce myocardial reserve, and heighten oxidative stress, all of which can exacerbate the cardiac burden imposed by chemotherapeutic agents [143,144].

According to the results, most notable CVD complications were cardiomyopathy, MI, cardiotoxicity, LVD, pericarditis, QT prolongation, and declining LVEF. Another finding of this review was the critical role of timing in the onset of cardiovascular complications, which was in line with the findings reported by Siaravas et al. [3]. Early-onset complications frequently include hypertension, arrhythmias (including QT prolongation from direct drug toxicity and inflammation), pericarditis/pericardial effusion (reflecting inflammatory processes), and subclinical myocardial injury indicated by declining LVEF and LVD. By contrast, late-onset complications primarily stem from cumulative damage, notably cardiomyopathy and HF due to anthracycline cardiotoxicity and chronic ischemic/microvascular injury. MI can occur with variable timing but often reflects progressive endothelial dysfunction and cumulative ischemic damage. Primarily, HF and hypertension are due to cardiotoxic effects of treatments like anthracyclines and TKIs, as well as secondary factors such as endothelial dysfunction, renal impairment, and corticosteroid use. Arrhythmias arise from myocardial structural and electrical remodeling caused by drug toxicity and leukemia-related inflammation. Cardiomyopathy and myocardial infarction may reflect cumulative ischemic and microvascular damage. QT prolongation, pericarditis, and pericardial effusion highlight the range of electrical and inflammatory cardiac issues, while declining LVEF and LVD indicate early subclinical myocardial injury. These findings are also supported by previous studies [3,145,146,147].

### Research Implications

Early detection of subclinical cardiac injury for patients with AL enables timely intervention through dose adjustments, cardioprotective therapies, or modification of treatment regimens. To achieve this, longitudinal follow-up is of paramount importance and must be stratified by risk, started before treatment, and extended for years post-therapy, particularly for pediatric and adult survivors. This includes baseline and serial advanced imaging using comprehensive echocardiography at baseline regularly during and after treatment, and incorporating sensitive measures such as GLS to detect subclinical systolic dysfunction before declines in LVEF. Routine biomarker monitoring including cardiac troponins and natriuretic peptides (e.g., NT-proBNP) is also essential to identify early myocardial injury or stress, especially around high-risk treatments like anthracycline cycles. Treatment-specific vigilance is also critical. For example, for anthracyclines, careful cumulative dose tracking and lifelong monitoring for delayed cardiomyopathy and heart failure through imaging are needed [105]; for TKIs especially Ponatinib, aggressive blood pressure management and assessment for arterial thrombotic events are necessary [148]; and for HSCT recipients, long-term screening for heart failure, arrhythmias, and accelerated atherosclerosis should consider the effects of conditioning regimens and GVHD [109].

In high-risk CVD patients, anthracycline-sparing regimens or liposomal formulations can be considered to minimize cardiotoxicity. Those with CHIP-associated mutations warrant intensified cardiac surveillance and, where feasible, selection of less cardiotoxic therapeutic agents. Pre-existing hypertension necessitates cautious over TKI selection, particularly with agents like Ponatinib, and aggressive blood pressure optimization prior to initiation. Identifying cardiovascular risk factors at diagnosis should prompt structured planning, including baseline CVD risk assessment; treatment adjustment based on risk profile, and tailored surveillance throughout therapy and survivorship. In addition, comprehensive management of modifiable risk factors such as hypertension, dyslipidemia, and diabetes, along with lifestyle counseling throughout the care continuum is essential for reducing cardiovascular complications. In the future, the integration of novel data, including genetic markers like CHIP-associated mutations and pharmacogenomic variants such as CBR3 and RAC2, combined with biomarker trends will enhance risk prediction models. This will allow more personalized surveillance schedules and preemptive interventions tailored to individual patient risk profiles, improving long-term cardiovascular outcomes in patients treated for acute leukemia and related conditions.

## 5. Limitations and Future Works

One of the limitations of the current study was related to the heterogeneity of the study populations, outcome measures, and definitions of cardiovascular events. This precluded meta-analysis and limits the generalizability of the findings. Additionally, most studies were observational, with intrinsic risks of bias and confounding variables. Another limitation was related to the certain number of databases and English papers which were searched in this study. In fact, papers which might not be in English, their full text were not available; or were indexed in other databases, were not included in the current study. Therefore, this study can be expanded in the future to include more databases and papers in other languages. In addition, future research should prioritize prospective, multicenter studies that utilize standard definitions and outcome measures to ensure data comparability and generalizability. There is a critical need for the development and validation of cardiovascular risk prediction models specifically tailored to AL patients, accounting for disease-specific pathophysiology and treatment exposures. Finally, long-term follow-up studies to elucidate the trajectory of cardiovascular risk in AL survivors, especially pediatric and young adult cohorts are recommended.

## 6. Conclusions

This systematic review comprehensively investigated the risk factors associated with cardiovascular complications in AL patients. Cardiovascular complications arise from a complex interaction of treatment-derived toxicities, patient-specific vulnerabilities, and underlying genetic predispositions, manifesting across a spectrum from acute events to late-onset sequelae. Integrating sensitive biomarkers, genetic data, and advanced cardiac imaging enables early detection of subclinical injury and supports risk-stratified monitoring. Validating risk prediction models that combine genetic, biomarkers, and clinical data, along with adopting personalized medicine is essential to implement cardioprotective interventions and improve long-term cardiovascular outcomes in this group of patients.

## Figures and Tables

**Table 1:** Summary of the included studies.

No.	Authors/Year	Research Methodology	Research Objective	Type of Acute Leukemia	Risk Factors	Type of Cardiovascular Complications	Time of Cardiovascular Complication Onset	Key Findings
1	Udin et al. [29]/2024	Quantitative study (retrospective, observational post-mortem analysis)	To examine histopathological features alongside cardiovascular biomarkers in patients with Hematological Malignancies and Solid Tumors who underwent post-mortem evaluation	AML, ALL	BMI Cardiac biomarkers (e.g., B-type natriuretic peptide, cardiac troponin I) Echocardiographic parameters (e.g., ejection fraction) Systolic and diastolic blood pressure History of smoking Myocardial morphology Cancer treatment exposures Cell biomarkers (e.g., blast, lysozyme)	MI, CAD, arrhythmias, pericarditis, valvular heart disease, AF, cardiomyopathy, myocardial fibrosis, cardiac dysfunction, hypertension, electromechanical dysfunction, severe atherosclerosis	Early-Onset to Late-Onset	Patients with hematological malignancies, including acute leukemia, exhibited significant myocardial damage, marked by reduced cardiomyocyte nuclear density and disorganized collagen fibers, more so than in solid tumor cases. They also showed elevated BNP and low hemoglobin, indicating worsened cardiac dysfunction. Multivariate analysis identified increased right ventricular thickness, low diastolic blood pressure, and elevated cardiac troponin I as key predictors of cardiac death.
2	Desai et al. [30]/2024	Quantitative study (Phase 1 clinical trial)	To assess the safety, tolerability, pharmacokinetics, and antitumor activity of AZD5991 (investigational MCL-1 inhibitor), both as a monotherapy and in combination with venetoclax, in patients with relapsed or refractory hematologic malignancies	AML	Hypertension Existing cardiovascular disease conditions (e.g., cardiac ischemia, cardiac arrhythmia) Cardiac biomarkers History of cardiotoxic cancer treatments (e.g., anthracyclines) Comorbidities (e.g., diabetes, hyperlipidemia)	Cardiac arrest	Early-Onset	AZD5991 treatment, a human Myeloid Cell Leukemia 1 type inhibitor, was associated with cardiovascular complications, including elevated troponin in 10.3% of patients and increased troponin T in 54 of 65 cases. Importantly, no correlation was found between troponin elevation and pre-existing cardiovascular risk factors.
3	Poudel et al. [31]/2024	Quantitative study (population-based case–control cohort study)	To determine if serum proteins and/or metabolites in asymptomatic childhood cancer survivors can discriminate symptomatic cardiomyopathy	ALL	27 serum proteins Genetic factors (e.g., A-Kinase Anchoring Protein 4) Molecular factors (e.g., Tubulin Alpha 4a) Existing cardiovascular disease conditions (e.g., subclinical cardiac dysfunction) History of cardiotoxic cancer treatments (e.g., anthracyclines)	Subclinical cardiomyopathy, severe cardiomyopathy	Late-Onset	The study identified 27 dysregulated serum proteins predicting severe cardiomyopathy with 83% accuracy. Key proteins (A-Kinase Anchoring Protein 4, splicing factor 3b subunit 1, Tubulin Alpha 4a) revealed mechanisms of anthracycline-induced damage, and the model outperformed traditional tools, supporting early detection and prevention in cancer survivors.
4	Spannbauer and Bergler-Klein [32]/2024	Qualitative study (narrative review)	To evaluate the risk of ATE, MI, and ischemic stroke in cancer patients, particularly those with acute leukemia, explores the relationship between cancer treatments and cardiovascular toxicity, alongside the role of anticoagulation and antiplatelet therapies in preventing ATE	ALL, AML	Age Sex Type of leukemia Pre-existing cardiovascular conditions (e.g., AF, VTE, inflammatory myocardial damage)	ATE, MI, ischemic stroke, cardiomyopathy, AF, HF, cardiotoxicity	During Treatment to Late-Onset	Acute leukemia patients were at high risk for cardiovascular events, particularly in the first 6 months of diagnosis and treatment. Anthracyclines and radiation heighten cardiotoxicity, while AF increases ATE risk. The stroke risk score in AF underestimates this risk, and the efficacy of anticoagulation (Low Molecular Weight Heparin, Direct Oral Anticoagulants, warfarin) in prevention remained unclear.
5	Sameer et al. [33]/2024	Quantitative study (longitudinal cohort study)	To assess cardiac autonomic function and levels of endothelial and inflammatory biomarkers in adult patients with ALL immediately after chemotherapy and at a three-month follow-up	ALL	Height Weight BMI Waist circumference Hip circumference Waist-to-hip ratio Heart rate Cardiovascular autonomic function tests (e.g., heart rate variability, time-domain heart rate variability, frequency-domain heart rate variability) Endothelial dysfunction (e.g., high-sensitivity C-reactive protein) Inflammatory markers (e.g., Soluble Vascular Cell Adhesion Molecule-1, Soluble Intercellular Adhesion Molecule-1)	Cardiovascular autonomic dysfunction, endothelial dysfunction	Early-Oonset	ALL patients showed heightened sympathetic activity, reduced parasympathetic modulation, and autonomic imbalance, indicating elevated cardiovascular risk. While autonomic function improved over time, persistent levels of Soluble Vascular Cell Adhesion Molecule-1, Soluble Intercellular Adhesion Molecule-1, and High-sensitivity C-Reactive Protein suggested ongoing endothelial dysfunction and inflammation.
6	Salas et al. [34]/2024	Quantitative study (retrospective, multicenter, registry-based cohort study)	To investigate the incidence and predictors of early (first 100 days) and late cardiac events after allo-HCT in patients with AML treated with anthracyclines and explore the impact of post-transplant cyclophosphamide on cardiac complications and the effect of cardiac events on overall survival and nonrelapse mortality	AML	Age at diagnosis Time to diagnosis Time to diagnosis for late cardiac events (late cardiovascular events) Prophylaxis type (posttransplant cyclophosphamide-based vs. other GVHD prophylaxis) Transplant procedure parameters (donor type, conditioning regimen intensity) Pre-existing cardiovascular conditions (e.g., arrhythmia, HF, myocardial ischemia) Severity of cardiovascular events (CTCAE Grade)	HF, MI, ischemia, arrhythmias, pericardiac effusion or pericarditis, moderate valvulopathy disease	Early-Onset	Early cardiac events (5.5% incidence) were linked to posttransplant cyclophosphamide use and preexisting cardiac risks before allo-HCT and were associated with higher nonrelapse mortality and lower overall survival. Late cardiac events (2.8% incidence) had no significant impact on nonrelapse mortality or overall survival.
7	Roganovic et al. [35]/2024	Qualitative study (narrative review)	To raise awareness about the long-term adverse effects experienced by survivors of childhood acute leukemia, emphasizing the need for structured long-term surveillance and standardized follow-up care to manage these effects effectively	ALL, AML	Type of leukemia Age Sex Genetic factors Health behavior Risk stratification group Physical activity Alcohol consumption History of smoking Pre-existing cardiovascular conditions Comorbidities (e.g., diabetes, hyperlipidemia, obesity)	Arrhythmias, cardiomyopathy, CAD, pericardial disease, valvular heart disease	Late-Onset	Leukemia treatments, especially in childhood cancers, caused serious long-term complications, including second malignancies, cardiovascular issues, and chronic health conditions. Cardiovascular complications were a significant concern after leukemia treatment, especially in patients who received high doses of anthracyclines or radiation. These complications occurred during treatment, early-onset or late-onset, with long-term risks of HF, hypertension, and CAD.
8	Puła et al. [36]/2024	Quantitative study (pilot before–after study)	To determine the clinical utility of the new ST2 marker and to routinely assess cardiac parameters in patients undergoing HSCT	ALL, AML	Pre-existing cardiovascular conditions Type of leukemia Echocardiographic parameters Cardiac biomarkers (e.g., troponin T, NT-proBNP, ST2) Inflammatory markers (e.g., Soluble CD40 Ligand, TNF-α, IL-6, IL-18, IL-10, Monocyte Chemoattractant Protein-1) Molecular factors (e.g., tyrosine kinase with immunoglobulin-like and EGF-like domains)	Subclinical myocardial damage, Vascular disorders, Arrhythmias, HF, Cardiovascular death	During Treatment and Early-Onset	Echocardiographic GLS and biomarkers effectively predicted cardiovascular complications, detecting subclinical myocardial damage before symptoms appeared. Leukemia treatment history influences dysfunction severity, and patients with pre-existing cardiovascular risks were less likely to receive HSCT, indicating the need for early risk assessment.
9	Onoue et al. [37]/2024	Quantitative study (retrospective cohort study)	To compare the incidence of MACE in patients with AML treated with venetoclax versus anthracyclines	AML	Age Sex BMI Hypertension Comorbidities (e.g., hyperlipidemia, diabetes mellitus, chronic kidney disease) Pre-existing cardiovascular conditions (e.g., CAD, HF, AF) Pharmacological treatments (ACE inhibitors, angiotensin receptor blockers, beta-blockers, statins, aspirin) Echocardiographic parameters (e.g., LVEF, echo screening) Time to event Cumulative incidence rates (e.g., 1-year risk of cardiotoxicity)	HF, MACE, AF, MI	During Treatment and Early-Onset	Venetoclax-treated patients showed a higher incidence of cardiovascular complications, including earlier-onset MACE and new AF, compared to those on anthracyclines. While HF rates were similar, venetoclax was linked to significantly higher mortality (78% vs. 41%). Echocardiogram use was more frequent in the anthracycline group, despite slightly lower LVEF.
10	Ma et al. [38]/2024	Quantitative study (observational cohort study)	To develop and validate a personalized predictive nomogram and risk score to assess the risk of cardiac injury before chemotherapy in patients with newly diagnosed AML	AML	Age Sex History of smoking Comorbidities (e.g., hyperlipidemia, diabetes) AML subtypes Blood count parameters (WBC, RBC, HGB, PLT) BM blast ratio Genetic mutation types (oncogenic, tumor suppressor, epigenetic, chromosomal translocations) Cytogenetics (e.g., karyotype complexity, monosomy karyotype, Philadelphia chromosome) Cardiac biomarkers (NT-proBNP) Echocardiographic parameters (e.g., left atrium, LV, inter-ventricular septum, EDV, ESV, EF)	Cardiac injury, Cardiac enzyme abnormalities, Cardiac-related lesions, Cardiotoxicity, Other cardiac alterations	Before Treatment	The study identified four independent risk factors, including abnormal NT-proBNP, NPM1 mutations, and elevated WBC and RBC counts for cardiac injury in newly diagnosed AML patients. A predictive nomogram showed high accuracy (AUCs: 0.742, 0.750, 0.706), and higher risk scores were linked to worse overall survival in AML patients.
11	Liu et al. [39]/2024	Qualitative study (narrative review)	To investigate cardiotoxicity prevention strategies in patients receiving anthracyclines, particularly in children with acute leukemia	ALL, AML	Cardiac biomarkers (NT-proBNP, cTnT, hs-cTnT) Electrocardiographic parameters (e.g., QTc, LV dysfunction, abnormal electrophysiological changes, dynamic loading ECG, early adverse events, coronary diffuse graft ischemia) Echocardiographic parameters (e.g., LVEF, LVFS, GLS, RV function and size, pulmonary artery systolic pressure, radionuclide angiography, CMRI)	LVD, HF, Myocardial fibrosis, Hypertension, Arrhythmias, Endothelial dysfunction	Before Treatment to Late-Onset	Anthracyclines posed a dose-dependent cardiotoxicity risk. Dexrazoxane reduced early toxicity but raised concerns about secondary malignancies. Liposomal anthracyclines were less cardiotoxic, and long-term cardiac monitoring was essential. Combination therapies (beta-blockers, ACE inhibitors, statins) showed potential for cardio protection but needed further validation.
12	Kępski et al. [40]/2024	Quantitative study (observational retrospective analysis cohort study)	To analyze the time-dependent relationship between the occurrence of venous thromboembolic (VTE) and arterial thromboembolic (ATE) events and the diagnosis of hematological malignancies, as well as the initiation of onco-hematological treatment	ALL, AML	Type of leukemia Comorbidities Age Chemotherapy initiation Disseminated intravascular coagulation in AML Thromboembolism (VTE or ATE)	VTE, ATE	During Treatment to Late-onset	AML and ALL raised VTE risk during treatment, with chemotherapy and disseminated intravascular coagulation contributing to AML, and higher VTE rates were seen in ALL. ATEs, often preceded cancer diagnosis in older patients, were linked to CAD but not general cardiovascular comorbidities. VTE and ATE impacted prognosis and treatment strategies in hematologic malignancies.
13	Hellman and Chaireti [41]/2024	Quantitative study (retrospective cohort, single-center study)	To evaluate the incidence and risk factors of arterial thromboembolic events in a cohort of patients with acute leukemia and lymphoid malignancies during a 15-year period	ALL, AML	Age at diagnosis Sex BMI Cancer type and aggressiveness ATE at diagnosis Type of ATE History of ATE prior to cancer diagnosis Age at ATE after diagnosis Cancer treatment at ATE Blood count parameters (PLT) History of smoking Comorbidities (e.g., hyperlipidemia, diabetes) Pharmacological treatments (lipid-lowering or antihypertensive medication) Antiplatelet therapy Hypertension Infection	Thromboembolic Stroke, AMI, Arterial Thrombosis	Before Treatment to Early-Onset	The 15-year incidence of ATE was 1.4%, similar between AL and lymphoid malignancies. 68.8% of ATE cases were in male patients, which aligned with known higher cardiovascular risk in men. Many ATE cases in AL patients occurred at the time of diagnosis, before treatment initiation. A low platelet count was not protective against ATE, and some patients developed ATE despite severe thrombocytopenia. Almost 50% of patients did not restart antiplatelet agents after platelet recovery (>50 × 10^9^/L), which may have increased ATE risk.
14	Hammoud et al. [42]/2024	Quantitative study (prospective, longitudinal cohort study)	To characterize the prevalence of MACE and its association with the cumulative burden of non-MACE in survivors of childhood cancer	ALL, AML	Sex Race/ethnicity Age at diagnosis Length of follow-up Treatment era Primary cancer diagnosis Cancer treatment exposures	MACE includes Cardiomyopathy, HF, LVSD, MI, and Stroke Non-MACE includes Arrhythmias, prolonged QT, Hypertension, Dyslipidemia, Structural defects, Ventricular dysfunction, vascular disease.	During Treatment to Late-Onset	The cumulative burden of non-MACE conditions significantly raised the risk of MACE, with one non-MACE condition increasing the risk 4.3-fold and four or more increasing it 11.1-fold. Major contributors included chest radiation ≥30 Gy and high-dose anthracyclines ≥250 mg/m^2^. Subclinical cardiovascular issues strongly predicted future MACE. Survivors of AML had the highest MACE burden by age 50. Black survivors and males carried a higher cumulative burden than their counterparts.
15	Hammoud et al. [43]/2024	Qualitative study (state-of-the-art review)	To provide a comprehensive summary of the health consequences associated with cardiometabolic risk factors and frailty in survivors of childhood cancer and outline current recommendations for detection, prevention, and treatment while identifying knowledge gaps	ALL	Hypertension Comorbidities (e.g., diabetes, obesity, dyslipidemia, frailty) Cancer treatment exposures (radiation, cranial radiation) Pharmacological cancer Treatment (alkylating agents, high-dose glucocorticoids) Genetic predisposition	Cardiomyopathy, CAD, Valvular heart disease, Arrhythmias, Hypertension, Pericardial disease	Late-Onset	Childhood cancer survivors were at a significantly higher risk of developing CVDs compared to the general population. Cardiometabolic risk factors (obesity, hypertension, diabetes, and dyslipidemia) contributed to increased morbidity and mortality. Survivors remained underdiagnosed and undertreated for these conditions.
16	Fernández-Avilés et al. [44]/2024	Quantitative study (cross-sectional study)	To assess diastolic function in long-term survivors of childhood ALL using left atrial strain and conventional echocardiographic parameters	ALL	Age at diagnosis Age at examination Time since diagnosis Sex Weight Height BMI Body surface area History of smoking Systolic and diastolic blood pressure Heart rate Hypertension Hypercholesterolemia Comorbidities (e.g., diabetes mellitus, obesity, hypercholesterolemia) Sedentarism History of cardiotoxic cancer treatments Echocardiographic parameters (left ventricular measurements, left atrial strain, conventional diastolic parameters)	DD, Subclinical LVSD, Increased left atrial stiffness and reduced LAS values	Late-Onset	DD was present but subtle and was better detected with LAS than with conventional echocardiography. Patients exposed to higher doses of anthracyclines had more pronounced DD. Conventional echocardiographic parameters were not sensitive enough to detect early DD. LAS served as an early marker of cardiovascular dysfunction in childhood leukemia survivors.
17	Kundavaram et al. [45]/2024	Quantitative study (cross-sectional study)	To evaluate the acute cardiotoxic effects of doxorubicin-based induction therapy in pediatric ALL patients using echocardiography	ALL including B-cell ALL, T-cell ALL	Age Sex Weight Height Body surface area Type of leukemia (ALL) Blood count parameters (HGB, PLT) Total leucocyte count Absolute neutrophil Absolute lymphocyte Biochemical parameters (potassium, calcium, phosphate) Renal parameters (uric acid) Echocardiographic parameters (right ventricular functions, left ventricular structure and function, LV tissue Doppler imaging)	Systolic and diastolic dysfunction of both ventricles, Decreased LVEF and fractional shortening, right ventricular dysfunction, with a decrease in TAPSE and an increase in myocardial performance index, DD by reduced mitral and tricuspid E/A ratios and prolonged Isovolumic Relaxation Time	Early-Onset	Doxorubicin-induced cardiotoxicity can occur acutely, within 72 hours of administration. Both left and right ventricular dysfunctions were detected using echocardiography. The diastolic function was affected earlier than systolic function. Subclinical cardiac dysfunction was detected, emphasizing the need for early echocardiographic monitoring. The observed changes were not severe, but long-term follow-up was necessary to determine if these effects progressed.
18	Dogliotti et al. [46]/2024	Qualitative study (narrative review)	To maintain efficacy while reducing toxicity in radiation-based conditioning regimens for HSCT, which includes evaluating total marrow and lymphoid irradiation as an alternative to TBI to reduce side effects while maintaining treatment effectiveness	ALL, AML	Existing cardiovascular disease conditions (e.g., dyslipidemia and cardiotoxicity from treatments) Hypertension Comorbidities (e.g., pulmonary risks, endocrine complications, hypothyroidism, insulin resistance, dysregulation) Risk of secondary malignancies TBI-associated late toxicities	Hypertension, Cardiotoxicity	Early-Onset to Late-Onset	Total marrow irradiation and total marrow and lymphoid irradiation regimens may reduce cardiovascular toxicity compared to traditional TBI. There was a high prevalence of metabolic and cardiovascular risks in leukemia survivors who received HSCT with TBI. Alternative conditioning regimens with lower radiation exposure mitigate toxicity while preserving efficacy.
19	Diaz et al. [47]/2024	Quantitative study (retrospective cohort study)	To assess the effect of female sex on the development of incident HF in adult patients treated with anthracyclines	ALL, AML	Age at first anthracycline dose Sex Race/ethnicity BMI Type of leukemia Cardiovascular risk factors Cancer treatment exposures (chest radiation, HSCT) Pharmacological cancer treatment (anthracycline agents) Anthracycline dosage in doxorubicin equivalents Cumulative doxorubicin-equivalent dose Received more than 1 agent	Cardiomyopathy, HF, Heart Failure with Reduced Ejection Fraction, Heart Failure with Mildly Reduced Ejection Fraction, Heart Failure with Preserved Ejection Fraction	Early-Onset	Female sex was not a risk factor for anthracycline-associated HF in adults, contrary to findings in pediatric populations. Older age, CAD, and HSCT were significant risk factors for developing HF. Leukemia patients had a higher HF risk due to a high rate of hematopoietic stem cell transplants, mostly presenting heart failure with preserved ejection fraction. Five-year HF incidence was 4.78%, consistent with prior data.
20	Rique et al. [48]/2024	Quantitative study (monocentric longitudinal cross-sectional study)	To analyze the profile of left ventricular alterations in children treated with anthracyclines and to examine risk and protective factors, including physical activity	ALL, AML	Age Age at leukemia diagnosis Age at start of treatment Sex Cancer treatment exposures (TBI 12 Gray) Pharmacological cancer treatment (anthracycline agents) Time from treatment to transesophageal echocardiograms in years Cumulative anthracyclines dose Physical activity before and after the treatment Echocardiographic parameters (e.g., interventricular septum thinning, decreased LV shortening fraction, decreased LVEF, abnormal LV GLS, increased left atrial volume, LVDD, altered TAPSE, valvular disease, pericardial abnormality, left ventricular mass indexed, LV shortening fraction, LVEF, LV GLS, RV GLS)	LVD, Dilated cardiomyopathy, LVDD, right ventricular dysfunction	Late-Onset	28.9% of patients had LV GLS abnormalities despite normal ejection fraction. Radiotherapy and high anthracycline doses (>240 mg/m^2^) increased cardiac dysfunction risk, while regular physical activity (>14 MET.h/week) was protective. LV GLS was more sensitive than LVEF for early cardiotoxicity detection. Dilated cardiomyopathy occurred in 3 patients.
21	Barachini et al. [49]/2024	Qualitative study (narrative review)	To describe advancements in understanding the molecular physiopathology of treatment-related adverse events and to emphasize strategies for predicting, detecting, and managing chemotherapy-induced cardiotoxicity	ALL and mentions Blinatumomab, a Cluster of Differentiation 19/Cluster of Differentiation 3 bispecific antibody used for its treatment	Pre-existing cardiovascular conditions Pharmacological cancer treatment (anthracycline agents, TKIs) High cumulative doses of anthracyclines Bispecific antibodies and their cytokine release syndrome effects Hypoxia and mitochondrial dysfunction leading to oxidative stress Pro-inflammatory cytokine activation (IL-6, reactive oxygen species, vascular endothelial growth factor) History of cardiotoxic cancer treatments (e.g., CAR-T cell therapy)	HF, Myocarditis, Arrhythmias, AF, QT prolongation, hypertension, ischemic events, VTE, pulmonary hypertension, Pericardial disease	During Treatment to Late-Onset	Cardiotoxicity was a major concern in leukemia treatment, requiring ongoing monitoring. Early detection strategies (biomarkers, imaging) were crucial in preventing severe complications. Mitochondrial dysfunction played a central role in chemotherapy-induced cardiac damage. Newer targeted therapies (BTK inhibitors, CAR-T cells) had specific cardiac risks that needed tailored management.
22	Ketterl et al. [50]/2023	Quantitative study (comparative cohort study)	To assess the insulin sensitivity and CVD risk factors in survivors of HCT and compare these factors with healthy sibling controls	ALL, AML	Age at study Age at diagnosis Years since diagnosis Age at most recent HCT Years since most recent HCT Sex Height Weight Waist circumference BMI Lean body mass Percent fat mass Total fat mass Type of leukemia Visceral adipose tissue Percent fat mass percentiles Cancer treatment exposures (HST, HSCT, radiation treatment) HSCT type Systolic and diastolic blood pressure Lipid profile (LDL-C, HDL-C, triglycerides, total cholesterol) Blood glucose Insulin resistance and homeostatic model assessment of insulin resistance	Dyslipidemia, Cardiometabolic risk	Late-Onset	HCT survivors had a higher prevalence of metabolic syndrome and cardiovascular risk factors than the general population and sibling controls. They showed insulin resistance, dyslipidemia (high total cholesterol, LDL, triglycerides, low HDL), and altered body composition with increased visceral fat and reduced lean body mass, even with normal BMI. TBI was strongly linked to these changes and elevated CVD risk. These factors contributed to early cardiovascular mortality, highlighting the need for early monitoring and intervention.
23	Wang et al. [51]/2023	Quantitative study (genome-wide association study (GWAS))	To identify genetic variants that modify the risk of anthracycline-related cardiomyopathy among childhood cancer survivors	ALL, AML	Age at diagnosis Sex Hypertension Pharmacological cancer treatment (anthracycline agents) Anthracycline dose Comorbidities (e.g., diabetes, dyslipidemia) Cancer treatment exposures (chest radiation) Genetic factors (e.g., genetic variants, rs17736312 in the Roundabout 2 gene)	Cardiomyopathy, anthracycline-related cardiomyopathy, CHF, HF, heart transplantation	Late-Onset	The rs17736312 SNP in roundabout 2 gene was linked to increased risk of anthracycline-related cardiomyopathy, especially at high doses. AA genotype carriers showed significantly higher heart failure risk. The Slit-Robo pathway promoted fibrosis via Transforming Growth Factor β1/Smad, with key gene–dose interactions influencing late-onset cardiotoxicity.
24	Zhou et al. [52]/2023	Quantitative study (observational prospective cohort study)	To explore biomarkers as early predictors of anthracycline-induced subclinical cardiotoxicity in acute leukemia patients	ALL, AML	Age Sex Type of leukemia Marrow blast percentage Sepsis during chemotherapy Grade IV myelosuppression BMI History of smoking Hypertension Lipid profile (LDL-C, HDL-C, triglycerides, total cholesterol) Renal parameters (urea nitrogen, creatinine, uric acid) Cardiac biomarkers (e.g., cTnT, NT-proBNP) Hepatic function (albumin, ALT AST, total bilirubin, direct bilirubin) Coagulation profile (D-Dimer, prothrombin time, APTT, fibrinogen, fibrinogen degradation products) Blood count parameters (WBC, RBC, HGB, PLT) Blood glucose Lactate dehydrogenase Echocardiographic parameters (mitral inflow E velocity, mitral e’ velocity, E/e’ ratio, LVDd, left ventricular mass indexed, FS, LVEF)	Anthracycline-Induced Subclinical Cardiotoxicity, Cardiac Dysfunction, Potential Risk of Progression to CHF, LVD, and Arrhythmias	During Treatment	17 of 51 patients developed anthracycline-induced subclinical cardiotoxicity after 3 chemotherapy cycles. Higher platelet count and blood glucose were linked to increased anthracycline-induced subclinical cardiotoxicity risk, while total and direct bilirubin might have been protective. Combining PLT and NT-proBNP had the best predictive value. Dynamic PLT changes after chemotherapy differed significantly between anthracycline-induced subclinical cardiotoxicity and non-anthracycline-induced subclinical cardiotoxicity groups.
25	Mitrovic et al. [53]/2023	Quantitative study (retrospective cohort study)	To determine the incidence of ATEs in non-promyelocytic AML patients and identify potential risk factors for ATE development	AML excluding promyelocytic AML	Age Sex History of smoking BMI Pre-existing cardiovascular conditions (thrombotic events) Eastern Cooperative Oncology Group performance status HCT comorbidity index Comorbidities Pharmacological treatments (antiplatelet, anticoagulant, beta-blockers, statins) Blood count parameters (WBC, HGB, PLT) Lactate dehydrogenase Coagulation profile (e.g., D-Dimer, APTT, fibrinogen, international normalized ratio) Blast peripheral blood CNS involvement Cytogenetic risk group Inserted central venous line Type of therapy (intensive, non-intensive/supportive)	Hypertension, HF, AF, Previous MI, Peripheral arterial disease, Stroke history ATEs which included Ischemic Stroke, Acute Lower Extremity Arterial Thrombosis,	During Treatment to Early-Onset	The incidence of ATE in AML patients was 2.9%, aligning with other cancer populations. Key risk factors included obesity (BMI > 30, 20× risk), prior thrombosis (4×), cardiovascular comorbidities (8×), and adverse cytogenetics (2×). ATE was associated with a high mortality rate (50%). The study indicated the need for primary thromboprophylaxis in high-risk AML patients, though bleeding risks from AML treatment posed challenges to implementation.
26	Kantarjian et al. [54]/2023	Quantitative study (phase 2 clinical trial)	To assess the long-term efficacy and safety of the frontline combination of ponatinib (a third-generation BCR::ABL1 tyrosine kinase inhibitor) and Hyper fractionated Cyclophosphamide, Vincristine, Adriamycin, Dexamethasone chemotherapy in treating Philadelphia chromosome-positive ALL (Ph+ ALL)	Ph+ ALL	Age Blood count parameters (WBC, HGB, PLT) CNS involvement Hypertension CD20 positivity Genetic factors (e.g., BCR:ABL1 transcript) Cytogenetics (diploid/intermediate metaphase, Ph-positive) Comorbidities (e.g., diabetes, dyslipidemia, CAD, peripheral arterial disease) Response parameters (e.g., complete remission, complete cytogenetic response, major molecular response, complete molecular response, minimal residual disease flow negativity) Treatment-related toxicities (e.g., bleeding, nausea, diarrhea, headache, infections during induction)	Hypertension, AOEs, MI, Unstable angina; Venous thromboembolic events including Pulmonary embolism, Renal vein thrombosis, DVT	During Treatment to Late-Onset	The combination of ponatinib and hyper fractionated Cyclophosphamide, Vincristine, Adriamycin, Dexamethasone achieved 100% complete response and 86% complete molecular remission, with strong long-term outcomes: 6-year event-free survival (65%) and overall survival (75%), largely avoiding the need for allo-SCT. However, cardiovascular toxicity was significant, with 52% developing hypertension (17% Grade 3), especially at 45 mg/day doses. Dose reductions (to 30 mg or 15 mg) improved safety without reducing efficacy. No fatal cardiovascular events occurred post-adjustment. Ponatinib also suppressed ABL1 T315I mutations effectively, with no relapses in these patients.
27	Heredia et al. [55]/2023	Quantitative study (cross-sectional study)	To assess right ventricular function in long-term survivors of childhood ALL using echocardiographic conventional measurements and automated RV strain	ALL	Age at diagnosis Age at exam Time since diagnosis Sex Weight Height BMI Body surface area Systolic and diastolic blood pressure Heart rate History of smoking Hypertension Comorbidities (e.g., diabetes, obesity, dyslipidemia, hypercholesterolemia) Sedentarism Cancer treatment exposures (radiotherapy, HSCT, cranial radiation) Pharmacological cancer treatment (anthracycline agents) Anthracycline dose ESC Guidelines Risk Category Echocardiographic parameters (right ventricle parameters, left ventricle parameters)	Right Ventricular Systolic Dysfunction, Subclinical Right Ventricular Dysfunction, Reduced TAPSE, Decreased Right Ventricular Four-Chamber Strain, LVD	Late-Onset	Significant reduction in RV function happened among childhood leukemia survivors compared to healthy siblings, despite normal conventional echocardiographic parameters. Right ventricular free-wall strain identified subclinical dysfunction in 16.7% of survivors, a much higher prevalence than conventional measures. HSCT was linked to lower TAPSE values, suggesting it contributed to long-term RV impairment. Modifiable risk factors (obesity and smoking) were strongly associated with worsening RV function.
28	Gawlik et al. [56]/2023	Qualitative study (narrative review)	To provide an update on epidemiology, risk factors, and management of cardiac arrhythmias in oncological patients within the context of the new European Society of Cardiology 2022 guidelines on cardio-oncology	APL	Age Race/ethnicity Comorbidities (e.g., endocrine complications, metabolic disorders, obesity, diabetes, abnormal renal or liver function) Hypertension Pharmacological cancer Treatment (immunotherapies, immune checkpoint inhibitors, CAR-T) Pre-existing cardiovascular conditions (e.g., arrhythmogenic substrate, congenital long QT syndrome) Existing cardiovascular disease conditions (e.g., post-surgery arrhythmia, electrolyte abnormalities, primary cancer of the heart, metastasis to the heart) Pharmacological treatments (arrhythmogenic medications)	QT Prolongation, VAs	During Treatment to Late-Onset	Arsenic trioxide was highly effective for APL treatment but posed a significant risk of QT prolongation and VAs. 63% of patients experienced QT prolongation, including a case of asymptomatic torsades de pointes. These complications ranged from moderate to severe and could be life-threatening without proper management. Onset occurred during treatment, requiring close ECG monitoring and correction of modifiable risk factors such as electrolyte imbalances. Management followed ESC 2022 guidelines for arrhythmias.
29	Fazal et al. [57]/2023	Qualitative study (retrospective case series and review of literature)	To describe and analyze cases of VAs associated with the use of TKIs in cancer patients	Ph+ ALL	Age Pharmacological cancer treatment (TKIs) Type of TKI Duration of TKI therapy Heart rate Cardiovascular risk factors Pre-existing cardiovascular conditions (e.g., structural heart disease, arrhythmias, electrolyte abnormalities)	VAs include non-sustained ventricular tachycardia, Sustained ventricular tachycardia, Ventricular fibrillation, Premature ventricular contractions	Early-Onset to Late-Onset	TKIs, including ibrutinib, zanubrutinib, dasatinib, and afatinib, were clearly associated with VAs. Arrhythmias often resolved after discontinuing TKIs, suggesting a drug-induced cause. However, some patients required long-term antiarrhythmic treatment or implantable cardioverter-defibrillators. Both those with and without prior cardiovascular disease were at risk, highlighting widespread susceptibility.
30	Boluda et al. [4]/2023	Quantitative study (retrospective cohort study)	To evaluate the incidence, timing, and impact of cardiac events in patients with acute leukemia undergoing intensive and non-intensive chemotherapy	AML	Age Sex Pre-existing cardiovascular conditions Electrocorticography Comorbidities (extramedullary disease) Type of leukemia Blood count parameters (WBC, HGB, PLT) Cell biomarkers (e.g., peripheral blood blasts, bone marrow blasts) Renal parameters (urea nitrogen, creatinine, uric acid) Blood glucose Hepatic function (albumin, ALT AST, bilirubin) Coagulation profile (D-Dimer, prothrombin time, APTT, fibrinogen) Lactate dehydrogenase Molecular factors (e.g., mutations FLT3-ITD, FLT3-ITD ratio, FLT3-TKD, NPM1, IDH) Cytogenetics (nedical Research Council Cytogenetic risk) Cancer treatment exposures Pharmacological cancer treatment (anthracyclines, FLT3 inhibitors) Previous anthracycline treatment	HF, Arrhythmias, MI, Pericarditis, Hypertension	During Treatment to Early-Onset	Cardiac events were more common in older patients (≥65 years) and those with pre-existing cardiovascular conditions. Anthracyclines, FLT3 inhibitors, and intensive treatments increased cardiovascular risk. Infections (sepsis, pneumonia) and poor performance status further elevated complications. Cardiac issues negatively affected overall survival and treatment continuity.
31	Bertrand et al. [58]/2023	Qualitative study (systematic review study)	To provide evidence on the prevalence, incidence, and risk factors of cardiac electrical abnormalities in childhood ALL survivors who have completed their treatment	ALL	Age at diagnosis Time since diagnosis Sex Length of follow-up Cancer treatment exposures (SCT) Pharmacological cancer Treatment (anthracycline, vincristine, prednisone) Cumulative dose of anthracycline exposure and absolute cumulative dosage Cardioprotective treatment Electrocardiographic parameters (e.g., prolonged QT, ST segment, T waves, pathological Q waves, no sinus rhythm, ventricular tachycardia, ventricular ectopy, atrial ectopy)	Arrhythmias, Repolarization Disorders, Depolarization Disorders and Pathologic Q-waves, Conduction Disorders, Unclassified Abnormalities	Early-Onset to Late-Onset	Cardiac electrical abnormalities in childhood acute ALL survivors were generally infrequent but showed wide variability by type; heart rate abnormalities (0–68%) and repolarization disorders (0–30%) were more common, while depolarization disorders were rare. No consistent risk factors were identified, though anthracycline exposure and radiotherapy emerged as potential contributors.
32	Baum et al. [59]/2023	Quantitative study (observational retrospective and prospective cohort study)	To analyze the utilization of preventive health care (cancer screening, cardiovascular screening, vaccination) among blood cancer survivors	ALL, AML	Age Time since diagnosis Time from last treatment Sex Length of follow-up Follow-up institutions Allogeneic or no allogeneic transplantation Cardiovascular screening Systolic and diastolic blood pressure Lipid profile Urine glucose Overweight information Weight reduction measures Advice to stop smoking Vaccination management (vaccination after blood cancer, verification of vaccination status, influenza, streptococcus pneumonia, diphtheria/tetanus)	Arterial hypertension	Late-Onset	Blood cancer survivors, especially allo-HSCT recipients, showed higher rates of cardiovascular complications and greater use of preventive care (screenings for blood pressure, lipids, diabetes) than the general population. Coordinated care between oncologists and general practitioners led to the highest preventive care use, with GPs boosting cancer screening rates and oncologists improving vaccination coverage.
33	Auberle et al. [60]/2023	Quantitative study (retrospective cohort study)	To describe the incidence of late cardiac events after allo-SCT, identify risk factors for their development, and evaluate their impact on overall survival	ALL, AML	Age Sex BMI Race/ethnicity History of smoking Hypertension Comorbidities (e.g., diabetes) Pre-existing cardiovascular conditions (e.g., arrhythmia, CAD, valvular disease, CHF) Transplant procedure parameters (donor type, transplant characteristics) Existing cardiovascular disease conditions (e.g., any cardiac event, arrhythmia, pericardial effusion)	HF, Decline in LVEF, Atrial Arrhythmias, VAs, CAD, Pericardial Effusion, Pericarditis	Late-Onset	Cardiovascular complications affected 22% of allo-SCT survivors within 5 years. Pre-existing conditions like HF, hypertension, diabetes, and CAD were strong predictors of late cardiac events, while transplant-related factors (TBI, conditioning regimen, TKI use) were not significantly associated. These late cardiac events severely impacted overall survival, with atrial arrhythmias carrying the highest mortality risk (High Risk 10.6).
34	Alpman et al. [61]/2023	Quantitative study (retrospective population-based single-center cohort study)	To assess cardiovascular toxicity, including systemic hypertension, pericardial effusion, and thrombosis, in children undergoing anthracycline-containing treatment for ALL and to evaluate the effectiveness of standard cardiovascular surveillance methods	ALL	Sex Age at diagnosis Length of follow-up Comorbidities (e.g., diabetes mellitus, trisomy 21, celiac disease, asthma, periodic fever) Cancer treatment exposures (e.g., HSCT, Nordic Organization for Pediatric Hematology and Oncology ALL protocols, Escalated Phase ALL treatment protocol) Pharmacological cancer treatment (doxorubicin) Cumulative doxorubicin-equivalent doses Type of leukemia Echocardiographic parameters (at baseline, during treatment, 6 months or later, after end of treatment, LVEF) Existing cardiovascular disease conditions (e.g., endocarditis, intracardiac thrombosis, pericardial effusion)	Systemic hypertension, Intracardiac thrombosis, Pericardial effusion, QTc prolongation and arrhythmia risk, Mild cardiac dysfunction, LVEF, Severe HF	During Treatment to Late-Onset	Standard echocardiographic parameters (LVEF, LV Shortening Fraction) failed to detect early cardiac dysfunction, highlighting the need for more sensitive methods like GLS. Hypertension occurred in 20% of patients, mainly due to corticosteroid therapy, but resolved in all cases. Intracardiac thrombosis was observed in 8.5% of patients, treated successfully with Low Molecular Weight Heparin. One case of early severe HF occurred despite standard echocardiographic surveillance, indicating the importance of individualized monitoring.
35	Berisha et al. [62]/2023	Qualitative study (narrative review)	To examine the cardiovascular toxicity of TKIs used in treating Ph-positive leukemias and other cancers, with a particular focus on the effects of ponatinib, as well as other TKIs like sorafenib, sunitinib, lapatinib, gefitinib, and erlotinib	ALL, Ph-positive ALL	Hypertension Existing cardiovascular disease conditions (LVD) Pre-existing cardiovascular conditions (AOEs) Genetic factors (protein kinase B key signaling molecule, extracellular signal-regulated kinase signaling disruption) Cancer treatment exposures (radiotherapy, HSCT, cranial radiation) Pharmacological cancer Treatment (agents, TKs) History of cardiotoxic cancer treatments	MI, Cardiomyopathy and CHF, Hypertension, AOEs, LVD	During Treatment to Late-Onset	Ponatinib carried the highest cardiovascular risk among TKIs, with notable rates of MI and AOEs. Nilotinib and dasatinib presented moderate cardiovascular risk, primarily associated with hypertension and LVD. Hypertension was common with all TKIs but differed in severity. Cardiovascular events occur even in patients without prior risk factors, pointing to TKI-induced cardiotoxicity rather than pre-existing conditions.
36	Januzzi et al. [63]/2022	Quantitative study (retrospective cohort study)	To investigate the prevalence of baseline risk factors in patients with adjudicated AOEs both non-serious and serious, and to examine the associations between these risk factors, including cardiovascular conditions, and the occurrence of these events	ALL	Age Sex History of cardiotoxic cancer treatments (TKIs) Duration of previous treatment with approved TKIs Pharmacological cancer treatment (TKIs) Resistant or intolerant to dasatinib or nilotinib Best response of major molecular response or better to most recent regimen containing dasatinib or nilotinib Genetic factors (BCR::ABL1 T315I mutation) Length of follow-up Duration of treatment Dose intensity Primary reason for discontinuation of medication Existing cardiovascular disease conditions (e.g., arterial hypertension, ischemic and non-ischemic cardiac disease) Comorbidities (hypercholesterolemia, obesity, diabetes mellitus)	Cardiac arrest, MI, Ischemic and hemorrhagic strokes, Superior mesenteric artery occlusion, Worsening of congestive HF, Peripheral ischemia, Mild to moderate tricuspid regurgitation, Intermittent ventricular tachycardia	During Treatment to Early-Onset	Leukemia therapies, particularly TKIs like nilotinib, were linked to increased cardiovascular events, including QTc prolongation and fatal AOEs. Many affected patients had pre-existing CVD or risk factors. Those with serious AOEs were more likely to be on antihypertensives, aspirin, and platelet inhibitors, underscoring the importance of baseline cardiovascular management. Despite these risks, long-term survival was comparable between patients with and without AOEs, indicating that early intervention and risk factor control were critical for improving outcomes.
37	Xiao et al. [2]/2022	Quantitative study (single-center retrospective observational study)	To investigate baseline cardiac function and the risk of CVDs in patients with new-onset acute leukemia (AL), explore CVD risk factors present before treatment, and their association with prognostic survival	ALL. AML	Age Sex Hypertension Comorbidities (diabetes) History of smoking Type of leukemia Blood count parameters (WBC, RBC, HGB, PLT) Cardiac biomarkers (creatine kinase–myocardial band, hs-cTnI, BNP) Renal parameters (urea nitrogen, creatinine) Hepatic function (ALT AST) Coagulation profile (D-Dimer) Hydroxybutyrate dehydrogenase Lactate dehydrogenase Echocardiographic parameters (e.g., left ventricular internal diameter, EDV, ESV, EF)	Myocardial damage, Heart dysfunction, Elevated biomarkers of cardiac injury (highly sensitive troponin I, B-type natriuretic peptide, lactate dehydrogenase), Changes in echocardiography parameters (left ventricular internal diameter, ejection fraction)	Before Treatment	The leukemia blast ratio was positively correlated with cardiac damage, indicating that leukemia progression was linked to heart complications. Cardiac damage was evident in newly diagnosed acute leukemia patients even before chemotherapy, suggesting that leukemia itself contributed to cardiovascular issues. Hyperleukocytic leukemia was associated with more severe myocardial enzyme changes, likely due to leukemic cell accumulation and microvascular obstruction. The left ventricular internal diameter was the best predictor of heart damage, with age and EF identified as independent prognostic risk factors.
38	Terada et al. [64]/2022	Quantitative study (observational histopathological cohort study)	To clarify the histopathology and mechanisms of cancer therapy-related cardiac dysfunction by evaluating myocardial tissue samples for fibrosis, cardiomyocyte abnormalities, epigenetic modifications (Tumor Protein 53 and H3K27ac histone modification) and seeks to identify risk factors for cancer therapy-related cardiac dysfunction	ALL, AML	Age Sex Genetic factors (increased expression of Tumor Protein 53 and H3K27ac) Comorbidities (e.g., diabetes, tumor) Cancer treatment exposures (chemotherapy, radiotherapy, other therapies) Pharmacological cancer treatment Pre-existing cardiovascular conditions (e.g., high blood pressure, arrhythmias, electrolyte abnormalities) History of cardiotoxic cancer treatments (e.g., prior radiation therapy) Existing cardiovascular disease conditions (e.g., fibrosis, HF symptoms, cardiomyocyte changes) Time from chemotherapy to onset of cancer therapy-related cardiac dysfunction Cardiotoxicity risk score	Cardiac Dysfunction, HF Reduction in LVEF, Myocardial Fibrosis, Arrhythmias, Pericardial Effusion, Cardiomyocyte Damage	During Treatment to Late-Onset	Leukemia patients with therapy-related cardiac dysfunction showed fibrosis, cardiomyocyte damage, and elevated Tumor Protein 53/H3K27ac, indicating stress and epigenetic changes. Late-onset cases (>4.2 years) had worse outcomes (HR 7.61). Type 1 dysfunction (e.g., anthracyclines) caused irreversible, dose-dependent damage; Type 2 (e.g., trastuzumab) was potentially reversible.
39	Perpinia et al. [65]/2022	Qualitative study (narrative review)	To examine the clinical manifestations, preventive strategies, and pharmaceutical management of cardiotoxicity in patients with hematologic malignancies undergoing anticancer drug therapy or HSCT	ALL, AML	Type of leukemia Pharmacological cancer treatment (chemotherapeutic class and agents) Cancer treatment exposures (HSCT) Existing cardiovascular disease conditions (cardiomyopathy incidence, cardiotoxicity)	HF, Arrhythmias, AF, Cardiomyopathy, Hypertension, Pericarditis and Pericardial Effusion, Pulmonary Arterial Hypertension, Arterial Thrombosis and MI, QT Interval Prolongation	During Treatment to Late-Onset	Cardiotoxicity was a major concern in leukemia treatment, especially for patients with pre-existing cardiovascular conditions, who faced significantly higher risk. Cardioprotective therapies like beta-blockers, ACE inhibitors, and statins offered promising protection, while dexrazoxane was FDA-approved specifically to prevent anthracycline-induced cardiotoxicity. Hematopoietic stem cell transplant survivors had a 4-fold increased risk of CVD compared to the general population.
40	Muggeo et al. [66]/2022	Quantitative study (case–control study)	To evaluate left and right cardiac chamber performances and vascular endothelial function in childhood ALL survivors treated with anthracyclines	ALL	Age BMI Waist circumference Systolic and diastolic blood pressure Fasting glucose Insulin resistance and homeostatic model assessment of insulin resistance Lipid profile (LDL-C, HDL-C, triglycerides, total cholesterol) Endothelial dysfunction (Endothelin-1, Adiponectin, High-Molecular-Weight Adiponectin) Echocardiographic parameters (LVEF, left and right myocardial performance index, left E/A ratio, right E/A ratio, TAPSE) Vascular ultrasound parameters (e.g., flow-mediated dilation, mean intima-media thickness, anterior posterior abdominal aorta diameter)	Left and right ventricular dysfunction, Systolic and diastolic dysfunction, Endothelial dysfunction, Increased left and right myocardial performance index, Reduced TAPSE, Higher arterial stiffness	Late-Onset	Childhood ALL survivors treated with anthracyclines showed subclinical cardiovascular impairment, affecting both left and right ventricular function, including reduced TAPSE indicating right heart dysfunction. Higher cumulative anthracycline doses correlated with worsening cardiac function. Additionally, survivors exhibited vascular Endothelial dysfunction, evidenced by reduced flow-mediated dilation.
41	Luo et al. [67]/2022	Qualitative study (systematic review)	To document clinical features, diagnostic procedures, treatments, and outcomes of ALL cases that initially present with cardiac symptoms and provide management recommendations to help avoid misdiagnosis and improper treatment	ALL include T-ALL and B-ALL	Age Sex Type of leukemia Heart rate Systolic and diastolic blood pressure First manifestation of CVDs (e.g., cardiac tamponade, massive pericardial effusion, cardiac intracavitary mass, leukemic myocardial infiltration) Symptoms and signs (e.g., progressive dyspnea, cough, chest pain, fever, syncope, paradoxical pulse, elevated jugular venous pressure, muffled heart sounds, hypotension, tachycardia, peripheral edema) Electrocardiographic parameters (e.g., sinus tachycardia with low voltage, inverted T waves, ST depression, ST elevation) Imaging examination (e.g., Chest radiography, coronary angiography, chest CT, PET-CT) Cancer treatment exposures (Cardiac surgery, pericardiocentesis for tamponade) Pharmacological cancer treatment (chemotherapy agents)	Cardiac tamponade, Cardiac masses, Myocardial Hypertrophy, Leukemic infiltration of coronary arteries, Thrombotic occlusion, Pericardial Effusion, Arrhythmias and Conduction Abnormalities	Before Treatment to Late-Onset	Cardiac symptoms could be the first presentation of ALL, often leading to misdiagnosis by cardiologists. T-ALL commonly presented with pericardial effusion or tamponade, while B-ALL is linked to cardiac masses. Chemotherapy often resolved cardiac complications, but outcomes varied; some achieved complete remission, others faced fatal outcomes due to chemoresistance, delays, or severe cardiac dysfunction. Accurate diagnosis relied on cardiac imaging and biopsy.
42	Lipshultz et al. [68]/2022	Quantitative study (cross-sectional study)	To assess the burden of potentially modifiable cardiometabolic risk factors among childhood cancer survivors and compare them with an age, sex, and race/ethnicity-matched general population to estimate future cardiovascular risk	ALL	Sex Age Race/ethnicity Time since diagnosis Age at diagnosis Cancer treatment exposures (e.g., radiotherapy) Pharmacological cancer treatment (doxorubicin) Cumulative doxorubicin dose Radiotherapy dose BMI Waist circumference Systolic and diastolic blood pressure Lipid profile (LDL-C, HDL-C, triglycerides, total cholesterol) Blood glucose Fasting glucose HbA1c Comorbidities (pre-diabetes, diabetes, metabolic syndrome) History of smoking Physical activity	Hypertension, Pre-hypertension, Dyslipidemia, Obesity, Metabolic Syndrome, Ischemic heart disease, HF	Early-Onset to Late-Onset	Survivors had higher rates of pre-hypertension and hypertension than the general population, with similar or better metabolic profiles (obesity, diabetes, dyslipidemia) compared to controls. Despite healthier lifestyle habits (less smoking, more physical activity), survivors faced a higher risk of ischemic heart disease and HF. Existing cardiovascular risk models underestimated risks in cancer survivors, emphasizing the need for specialized survivor-specific models.
43	Gonzalez-Manzanares et al. [69]/2022	Quantitative study (retrospective single-center cross-sectional cohort study)	To assess the utility of automated GLS in LVSD in long-term childhood ALL	ALL	Age at diagnosis Age at examination Time since diagnosis Sex Weight Height BMI Body surface area Systolic and diastolic blood pressure Heart rate History of smoking Hypertension Lipid profile (LDL, HDL, triglycerides, total cholesterol) HbA1c Comorbidities (e.g., hyperlipidemia, diabetes mellitus, obesity, hypothyroidism, hypercholesterolemia) Sedentarism Cancer treatment exposures (radiotherapy, HSCT) Pharmacological cancer treatment (anthracycline) Cardiac biomarkers (e.g., Hs-cTnI, NT-proBNP) Anthracycline dose Echocardiographic parameters (e.g., LV end-diastolic volume, LV end-systolic volume, left atrium volume, LVEF, GLS, mitral E/A ratio)	LVSD, LVEF, Subclinical LVSD with GLS < 18.5%, HF, Increased Hs-cTnI	Late-Onset	GLS was more sensitive than LVEF in detecting cardiotoxicity in long-term childhood leukemia survivors. 26.6% of survivors had subclinical LV dysfunction, while only 12.2% showed reduced LVEF. Higher anthracycline doses (>250 mg/m^2^), diabetes mellitus, and radiotherapy were significant predictors of LVEF reduction. Smoking and HSCT were independent predictors of reduced GLS. Biomarkers like NT-ProBNP and Hs-cTnI were not effective predictors of LV function. Automated GLS may have improved long-term cardiac monitoring for childhood leukemia survivors.
44	Cornelissen et al. [13]/2022	Quantitative study (case–control study)	To explore whether cardiovascular risk factors can predict intracranial hemorrhage in patients with acute leukemia	ALL, AML, APL	Sex Age BMI Type of leukemia History of smoking Alcohol consumption Hypertension Blood count parameters (PLT) Pharmacological treatments (anti-coagulation, platelet aggregation inhibitors) Disease status Treatment phase Remission induction Consolidation therapy Cancer treatment exposures (allo-SCT) Comorbidities (e.g., diabetes, hypercholesterolemia, ischemic heart disease)	Hypertension, Ischemic heart disease	Before Treatment to Early-Onset	Hypertension and ischemic heart disease were the strongest predictors of intracranial hemorrhage in acute leukemia, increasing risk by 12.9 and 12.1 times, respectively. Intracranial hemorrhage had a high mortality rate (47%) compared to non-intracranial hemorrhage patients (9%). Leukemia treatment itself (chemotherapy, SCT) contributed to vascular damage and bleeding risk, which may have worsened pre-existing cardiovascular conditions. Other cardiovascular risk factors showed a positive association with intracranial hemorrhage but were not statistically significant due to the small sample size.
45	Chianca et al. [70]/2022	Qualitative study (narrative review)	To investigate the relationship between CHIP and the risk of CVDs in patients with AML, particularly in those treated with anthracycline chemotherapy	AML	Genetic factors (e.g., CHIP-related mutations as a risk factor for CVDs) Biomarkers Cut-off or range of value found for biomarkers Inflammatory markers (IL-1, IL-6, C-reactive protein, TNF-α, myeloperoxidase) Cardiac biomarkers (Galectin-3, Growth Differentiation Factor-15, sST2) Cancer biomarkers (Cancer Antigen-125, CHIP) Pharmacological cancer treatment (anthracyclines)	HF, Cardiomyopathy (Dilated Cardiomyopathy), Arrhythmias, MI, Hypertension, Endothelial Dysfunction, Atherosclerosis	During Treatment to Late-Onset	CHIP-related mutations elevated cardiovascular risk in AML patients on anthracyclines. Elevated IL-6, TNF-α, C-reactive Protein, and Myeloperoxidase levels indicated inflammation and oxidative stress, predicting cardiotoxicity. Soluble Suppression of Tumorigenicity 2 was associated with poorer cardiovascular and leukemia outcomes, while pre-existing conditions like hypertension and atherosclerosis increased post-treatment complication risk.
46	Calvillo-Argüelles et al. [7]/2022	Quantitative study (retrospective cohort study)	To examine the association between CHIP-related mutations and the risk of cardiovascular events in patients with AML, as well as the impact of these events on mortality	AML	Age Sex BMI History of smoking Comorbidities (e.g., diabetes, dyslipidemia, obesity) Hypertension Pharmacological treatments (aspirin, statin, ACE inhibitor/ARB, beta-blocker, any cardiac medication) Pre-existing cardiovascular conditions (e.g., CVD, CAD, HF, stroke, atrial fibrillation) Existing cardiovascular disease conditions Echocardiographic parameters (e.g., LVEF) AML characteristics Genetic factors (e.g., CHIP-related mutations) Pharmacological cancer treatment (anthracycline) Cancer treatment exposures (allo-HSCT, allo-HCT) Cumulative anthracycline dose Complete remission 1 duration	HF, MI, Acute Coronary Syndrome, Arrhythmias, Transient Ischemic Attack, VTE, Hypertension and Hypotension, Pericardial Disease, Cardiomyopathy	During Treatment to Late-Onset	CHIP-related mutations were linked to a higher risk of cardiovascular events (HF, MI, arrhythmia) in AML patients. Anthracycline-based chemotherapy was strongly associated with both early and late-onset cardiotoxicity. Patients with cardiovascular events had higher mortality, and CHIP-related cardiovascular events independently predicted worse survival. Pre-existing CVD and traditional risk factors further increased the risk of complications.
47	Bottinor and Chow [71]/2022	Qualitative study (narrative review with educational focus)	To discuss strategies for identifying childhood cancer survivors at increased risk for late cardiotoxicity, current monitoring approaches, and prevention strategies to reduce CVDs burden	AML	Age at treatment Sex Genetic predisposition Hypertension Comorbidities (e.g., diabetes, obesity, dyslipidemia, insulin resistance) Physical activity History of smoking Pharmacological cancer treatment (anthracycline) Cumulative dose of anthracyclines Equivalence ratios for cardiotoxicity among different anthracyclines Cancer treatment exposures (radiation, HCT, chest radiation) Echocardiographic parameters (e.g., LVEF, abnormal strain, low LV mass) Infection-associated cardiotoxicity Cumulative exposure to multiple cardiotoxic agents	Cardiomyopathy, HF, Ischemic Heart Disease, Valvular Heart Disease, Stroke, LVD, Hypertension	Early-Onset to Late-Onset	Cumulative anthracycline exposure, especially mitoxantrone (10× cardiotoxicity of doxorubicin), greatly increased HF risk, often after years without symptoms. Studies suggested dexrazoxane was safe and effective in reducing cardiac toxicity in pediatric leukemia patients. Echocardiography, myocardial strain imaging, and cardiac MRI improved early detection of subclinical dysfunction. Pediatric cancer survivors required unique long-term cardiovascular management, as adult HF treatments did not fully apply.
48	Arnán Sangerman et al. [72]/2022	Qualitative study (narrative review)	To provide practical recommendations for the use of midostaurin in the management of FLT3-mutated AML, optimize the use of midostaurin, minimize adverse effects, and provide a useful guide for routine clinical practice	FLT3-mutated AML	Molecular factors (FLT3 mutations, FLT3-ITD, FLT3-TKD) Infections (fungal or bacterial) Resistance to FLT3 inhibitors Adverse event (nausea, hyperglycemia, vomiting, QTc interval prolongation, Grade 3–4 hematological abnormalities, neutropenia, leukopenia, diarrhea) Pharmacological cancer treatment (midostaurin) Cardiac biomarkers (NT-proBNP) Transthoracic echocardiogram Echocardiographic parameters (LVEF, SLG, ventricular dysfunction due to cardiotoxicity, QT prolongation)	QTc interval prolongation, HF	During treatment to Early-Onset	Midostaurin may have prolonged QTc, especially with other QTc-prolonging drugs; dose adjustment was needed if QTc > 500 ms. Anthracycline regimens increased HF risk, particularly in patients with hypertension or diabetes. Regular ECGs, electrolyte monitoring, and baseline cardiac assessments (echo, NT-proBNP, troponin) were essential. ACE inhibitors or beta-blockers may have prevented cardiotoxicity. Resistance arose via FLT3 mutations or pathway shifts, requiring adaptive management.
49	Petrykey et al. [73]/2021	Quantitative study (lifetime cohort study)	To identify genetic markers associated with treatment-related cardiovascular complications, particularly cardiotoxicity, in survivors of childhood ALL anthracycline-based chemotherapy	ALL	Age at diagnosis Sex Protocols (Dana-Farber Cancer Institute Protocol, treatment regimen followed) Prognostic risk group Pharmacological cancer treatment (anthracyclines) Pharmacological treatments (dexrazoxane) Dexrazoxane cumulative dose Doxorubicin cumulative dose Echocardiographic parameters (e.g., LVEF, FS, LV end-diastolic diameter) Genetic factors (single nucleotide variations tested (rs72648998, rs3829747, rs2303838), position of human genome build 19, beta, genes (e.g., ATP-binding cassette C5), genotype (CC/CT/TT, CC/CT + TT), genotype (11/12/22, 11/12 + 22)) Blood count parameters (WBC) Immunophenotype (T-cell markers) Length of follow-up Minimal residual disease at diagnosis CNS involvement	Cardiotoxicity, LVD, HF, Structural Changes, LV End-Diastolic Diameter	Late-Onset	Common Titin gene variants were linked to better cardiac function (higher LVEF and FS) and had a cardioprotective effect. Rare variants in several genes were associated with cardiovascular outcomes; some were protective, while others (like Carbonyl Reductase 1) increased cardiotoxicity risk. Rare ATP-binding Cassette C5 variants were protective in high-risk patients, while rare Carbonyl Reductase 1 variants increased risk in standard-risk patients. Rare Nucleotide-Oligomerization Domain 2 and Zinc Finger Protein 267 variants were significantly associated with FS and LVEF, suggesting a role in long-term cardiovascular risk.
50	Oka et al. [74]/2021	Quantitative study (single-center observational cohort study)	To clarify the association between HSCT and GLS and to evaluate the serial changes in GLS and LVEF before and after HSCT	ALL, AML	Age Sex History of smoking Type of leukemia Type of HSCT (BMT, peripheral blood stem tell transplantation, unrelated peripheral blood stem cell transplantation, cord blood transplantation) Hypertension Heart rate Comorbidities (e.g., diabetes, dyslipidemia) Pharmacological treatments (ACE inhibitors, angiotensin receptor blocker, beta-blockers, calcium blockers, diuretics) Cardiac biomarkers (troponin I, BNP) Cancer treatment exposures (TBI) Pharmacological cancer treatment (anthracyclines) Total anthracycline dose Total anthracycline dose HCT comorbidity index Blood count parameters (HGB) Renal parameters (creatinine, estimated glomerular filtration rate, Cystatin C) Echocardiographic parameters (e.g., LVEF, GLS, diastolic function, tricuspid regurgitation peak gradient, left ventricular diastolic/systolic dimension, inferior vena cava diameter)	GLS, LVEF, Diastolic dysfunction	Early-Onset	GLS decreased notably at 1-month post-HSCT and partially recovered by 6 months, indicating it as an early marker of subclinical dysfunction. LVEF also dropped at 1 month but normalized by 3 months, except in the low EF group where it continued to decline. DD (e’ and E/e’) persisted at 3 and 6 months. Lower baseline GLS predicted reduced LVEF at 6 months. BNP and troponin I levels rose at 1-month post-HSCT, signaling early myocardial stress, but returned to baseline by 6 months.
51	Lubas et al. [75]/2021	Quantitative study (retrospective cohort study with prospective medical assessment)	To examine the contribution of emotional stress and distress to cardiac health in adult survivors of childhood cancer	ALL, AML	Age at examination Age at diagnosis Sex BMI Race/ethnicity Type of leukemia Hypertension History of cardiotoxic cancer treatments (e.g., anthracyclines) Cancer treatment exposures (radiation, chest radiation, cranial radiation) Anthracyclines in doxorubicin equivalents History of smoking Physical activity Alcohol consumption Psychological and mental health factors (clinically significant stress/distress, perceived stress, post-traumatic stress symptoms, depression, anxiety, any stressor distress) Comorbidities (e.g., dyslipidemia, diabetes, metabolic syndrome) Existing cardiovascular disease conditions (cardiac dysrhythmia, cardiomyopathy, myocardial Infarction)	Arrhythmias, Cardiomyopathy, MI, Hypertension, Dyslipidemia, Metabolic syndrome	Late-Onset	Stress and emotional distress were associated with higher rates of hypertension, dyslipidemia, metabolic syndrome, and diabetes in survivors. Longitudinally, baseline stress predicted new cases of dysrhythmia, hypertension, and dyslipidemia over 3.9 years. About one-third of survivors had clinically significant distress, higher than community controls. Stress and distress were independent cardiovascular risk factors, emphasizing the need for psychological interventions to improve heart health in childhood cancer survivors.
52	Lazăr et al. [76]/2021	Qualitative study (narrative review)	To investigate the challenges and complications associated with anthracycline induced cardiotoxicity in children treated for leukemia and to summarize the current understanding of the mechanisms of cardiotoxicity, identify risk factors, and discuss strategies for monitoring and managing cardiac dysfunction in pediatric cancer survivors	ALL	Age at treatment Treatment-free interval Pre-existing cardiovascular conditions Cancer treatment exposures (radiation) Pharmacological cancer treatment (anthracycline) Type of anthracycline Total cumulative dose of anthracyclines Genetic factors (genetic polymorphisms)	Cardiomyopathy, HF, Arrhythmias, DD, Myocarditis and Pericarditis	During Treatment to Late-Onset	Cardiotoxicity from anthracyclines occurred even at low doses due to genetic variability, particularly CBR3 and Solute Carrier 28 family member 3 polymorphisms. They caused irreversible cardiomyocyte loss, leading to chronic heart failure with <50% 5-year survival after symptom onset. Early, systematic cardiac evaluation was essential. Dexrazoxane, an iron-chelating agent, effectively reduced cardiotoxicity without affecting chemotherapy efficacy.
53	El Amrousy et al. [77]/2021	Quantitative study (prospective randomized clinical trial)	To assess the possible protective effect of omega-3 fatty acids on early doxorubicin-induced cardiac toxicity in children with ALL	ALL	Age Sex Cardiac biomarkers (e.g., troponin I, creatine kinase–myocardial band, NT-proBNP) Oxidative stress markers (glutathione, malondialdehyde, superoxide dismutase) Echocardiographic parameters (e.g., LVFS, LV E/A, LV S, 2D-GLS)	Doxorubicin-induced cardiotoxicity, LVD and Myocardial injury	Early-Onset	Omega-3 fatty acids reduced oxidative stress, preserved cardiac function, and lowered cardiac biomarkers in children with ALL receiving doxorubicin, compared to controls. They were a potential cardioprotective agent against doxorubicin-induced cardiotoxicity in pediatric ALL patients.
54	kamarajuet al. [78]/2021	Qualitative study (narrative review)	To highlight the role of CYP450 and P-glycoprotein in drug interactions within the field of cardio-oncology and to provide an overview of cardiotoxicity related to cancer treatments and multidisciplinary treatment approach to reduce drug-related toxicities	AML	Pre-existing cardiovascular conditions Hypertension Comorbidities (e.g., diabetes, thrombosis) Pharmacokinetic interactions (drug interactions mediated by CYP450 and P-glycoprotein, mechanism of interaction, level of pathway interaction) Pharmacological cancer treatment (anthracycline, TKIs, antimetabolites) Cancer treatment exposures (HSCT) Pharmacological classification Existing cardiovascular disease conditions (cardiotoxicity from treatments)	HF, Systolic and diastolic dysfunction; Arrhythmias include atrial and VAs, QT prolongation, Bradycardia, heart block; Pericarditis, myocarditis, Hypertension, Thrombosis	During Treatment to Late-Onset	Drug interactions mediated by CYP450 and P-glycoprotein transporters significantly impacted cardiovascular outcomes. Anthracyclines, TKIs, and alkylating agents were among the most cardiotoxic treatments. HSCT survivors had a 4.5-fold higher risk of CVD compared to the general population.
55	Hoeben et al. [79]/2021	Qualitative study (narrative review)	To review the role of TBI in HSCT for pediatric ALL, including its immunosuppressive and anti-leukemic effects, clinical outcomes, toxicity, and potential future directions	ALL	Age Radiation targets Cancer treatment exposures (TBI, total marrow irradiation, total marrow and lymphoid irradiation, HSCT) Pharmacological cancer treatment (chemotherapy) TBI dose and fractionation Total marrow irradiation dose and fractionation Total marrow and lymphoid irradiation dose and fractionation Minimal residual disease as a predictor of relapses Existing cardiovascular disease conditions (cardiovascular and metabolic risks associated with cancer treatment exposures) GvHD	Metabolic syndrome, Atherosclerosis, Hypertension, Increased risk of MI, stroke, and peripheral vascular disease, Subclinical systolic and diastolic dysfunction	Early-Onset to Late-Onset	TBI was linked to higher risks of metabolic and CVDs compared to chemotherapy-based conditioning. Higher doses of TBI (>10 Gy) correlated with increased cardiometabolic traits (higher fasting insulin, blood pressure, adverse lipid profiles). Late cardiovascular effects required lifelong monitoring, as cardiac disease risk increased over time in childhood cancer survivors.
56	Gangaraju et al. [80]/2021	Quantitative study, thesis (retrospective cohort study)	To evaluate the long-term risk of coronary heart disease in blood or marrow transplant survivors and identify associated risk factors	ALL, AML	Age at bone marrow transplant Sex Race/ethnicity History of smoking Hypertension Cancer treatment exposures (Pre-BMT chest radiation, pre-BMT chemotherapy, pre-BMT radiation, TBI) Pharmacological cancer treatment (chemotherapy agents) Comorbidities (e.g., diabetes, dyslipidemia, obesity. chronic kidney disease, chronic graft vs. host disease) Pre-existing cardiovascular conditions (arrhythmia, venous thromboembolism, stroke) Stem cell source Type of conditioning regimen Type of BMT	Coronary Heart Disease, Hypertension, Dyslipidemia, Congestive HF, Arrhythmias, Stroke, VTE	Early-Onset to Late-Onset	BMT survivors had a 7.2–11.7 times higher risk of coronary heart disease than siblings, with autologous BMT recipients at greater risk due to older age and pre-BMT chest radiation. Major risk factors included older age at BMT, male sex, pre-BMT chest radiation, and pre-existing cardiovascular risks. Coronary heart disease risk increased by 4% for every 100 cGy rise in radiation dose.
57	Duléry et al. [81]/2021	Quantitative study (single-center retrospective cohort study)	To assess the incidence and clinical features of early cardiac events associated with post-transplant cyclophosphamide in allo-HSCT	ALL, AML	Age Sex Type of leukemia History of smoking Disease status at transplantation Hypertension Comorbidities (e.g., hyperlipidemia, diabetes, dyslipidemia, obesity) Pre-transplant risk assessment (disease risk index, Karnofsky performance status, comorbidity index) Pre-existing cardiovascular conditions (e.g., cardiac event before HSCT, LVSD) Pre-transplant treatment exposure (exposure to cyclophosphamide before HSCT, exposure to anthracyclines before HSCT) Cumulative Cy dose before HSCT Cumulative anthracycline dose before HSCT Transplant history (previous autologous HSCT or allo-HSCT) Transplant procedure parameters (donor type, conditioning regimen, graft source, antithymocyte globulin) Post-transplant cyclophosphamide Early cardiac event Relapse incidence Graft-versus-host disease (Acute Grade II–IV GVHD, Chronic GVHD)	LVSD, Arrhythmias, Pericarditis, Acute Coronary Syndrome	Early-Onset	Post-transplant cyclophosphamide was linked to a higher incidence of early cardiac events (19% incidence in post-transplant cyclophosphamide group vs. 6% in non-post-transplant cyclophosphamide group), with early cardiac events patients showing significantly lower 2-year survival (31% vs. 64%). Pre-transplant cyclophosphamide exposure, sequential conditioning, older age, and prior cardiac events increased early cardiac events risk. Traditional cardiovascular risk factors were not strongly associated. Lowering post-transplant cyclophosphamide doses may have reduced cardiac toxicity, but more research is needed.
58	Diesch-Furlanetto et al. [82]/2021	Qualitative study (narrative review)	To discuss the late effects of HSCT in ALL survivors, emphasizing the importance of long-term follow-up and transition to adult care to mitigate health risks and improve quality of life	ALL	Affected organ/organ system (endocrine, bone, ocular, kidney and liver dysfunction) Psychological and mental health factors (depression, anxiety, post-traumatic stress disorder, cognitive impairments) Long-term sequelae (e.g., chronic fatigue, persistent headaches, neurodegenerative risks) Cardiovascular risk factors (e.g., increased risk of hypertension, metabolic syndrome post-TBI, accelerated atherosclerosis) Length of follow-up Long-term follow-up TBI-based	Atherosclerosis CVD, Hypertension, CAD, Arterial Diseases, HF, Arrhythmias, Cardiac Dysfunction	During Treatment to Late-Onset	HSCT survivors had a significantly higher risk of CVD compared to the general population. Risk of premature CV-related death was 2.3 times higher in HSCT recipients. Metabolic syndrome was more prevalent in ALL survivors who underwent HSCT (39%) compared to those treated with chemotherapy alone (8%). Arterial diseases (atherosclerosis, stroke, and heart attack) were a major cause of long-term mortality in HSCT survivors.
59	Chow et al. [83]/2021	Quantitative study (pilot randomized controlled trial)	To determine the feasibility of a remotely delivered mobile health intervention aimed at improving diet and physical activity in hematologic malignancy survivors to reduce cardiovascular risk factors	Acute Leukemia	Age Medical history Time since diagnosis Disease status BMI History of smoking Pre-existing cardiovascular Conditions Systolic and diastolic blood pressure Lipid profile Hypertension Blood glucose Insulin HbA1c Functional capacity assessment (cardiopulmonary reserve assessment, submaximal exercise testing, 6-minute walk test) Comorbidities (e.g., diabetes, dyslipidemia) Physical activity Technology access	Hypertension, Dyslipidemia	Late-Onset	Cancer survivors, particularly those treated for hematologic malignancies, were at an increased risk of CVD. Lifestyle factors such as hypertension, dyslipidemia, diabetes, smoking, poor diet, and physical inactivity further contributed to cardiovascular risk. A mobile health intervention using Fitbit trackers, diet tracking apps, and a Facebook peer support group was feasible and acceptable among survivors. While no statistically significant differences were found between intervention and control groups, the intervention favored improved diet quality, physical activity, and cardiovascular risk factors.
60	Chen et al. [84]/2021	Qualitative study (narrative review)	To identify the current understanding of cardiovascular toxicity associated with CAR-T cell and bispecific T cell engager therapies in the treatment of relapsed and refractory hematologic malignancies	B cell ALL	Symptoms and signs (e.g., fever, hypotension, hypoxia) Cytokine release syndrome Cardiac biomarkers (e.g., troponins and NT-proBNP) High tumor load Delays in cytokine release syndrome management Existing cardiovascular disease conditions (e.g., LVSD and decompensated cardiac failure)	Hypotension requiring vasopressors, Sinus tachycardia, Atrial arrythmia, VAs, LVSD, Decompensated cardiac failure, Cardiovascular death	Early-Onset	Cardiovascular toxicity was a significant but under-researched side effect of CAR-T cell and bispecific T cell engager therapies. Cytokine release syndrome was the strongest predictor of cardiovascular events. High-grade cytokine release syndrome (grades 3–4) increased the risk of cardiovascular events, including arrhythmias and cardiac failure. Early use of tocilizumab to treat cytokine release syndrome reduced the incidence of cardiovascular events.
61	Burns et al. [85]/2021	Qualitative study (narrative review)	To review the recognition, risk factors, and management of cardiotoxicity associated with Anti-Cluster of Differentiation 19 Chimeric Antigen Receptor T-Cell therapy	B cell ALL	Age High disease burden Pre-treatment blasts >25% on bone marrow biopsy Cytokine release syndrome and concomitant cytokine release syndrome Risk factors of cardiotoxicity (high CAR-T dose, high intensity lymphodepleting regimen, addition of fludarabine to cyclophosphamide during lymphodepletion, higher peak of C-reactive protein) Cardiac biomarkers (e.g., troponin) Pre-existing cardiovascular conditions (e.g., DD, endothelial activation, severe thrombocytopenia) Pharmacological treatments (e.g., aspirin, statin, insulin, beta-blocker, renin–angiotensin–aldosterone system inhibitors) Comorbidities (e.g., hyperlipidemia, higher baseline creatinine) Existing cardiovascular disease conditions (e.g., CAD, aortic stenosis, lower pre-CAR-T treatment baseline EF) Risk stratification tools (Lee Criteria, Penn Criteria, American Society for Transplantation and Cellular Therapy Consensus Criteria) Pharmacological cancer treatment (tocilizumab)	Tachycardia, hjypotension, pulmonary edema, depressed LVD, cardiac failure, myocardial injury, Arrhythmias, ST-segment changes, cardiac arrest	During Treatment to Early-Onset	Cardiotoxicity was closely linked to severe cytokine release syndrome. Troponin elevation was a predictive marker of cardiovascular risk. Older adults and patients with pre-existing cardiac risk factors (CAD, DD, etc.) were at higher risk. Expedited administration of tocilizumab (IL-6 inhibitor) may have reduced cardiac risk. Pediatric patients tended to recover cardiac function, whereas adults suffered long-term effects or fatal events.
62	Linares Ballesteros et al. [86]/2021	Quantitative study (prospective descriptive cohort study)	To describe the incidence of early-onset cardiotoxicity in children with acute leukemia treated with chemotherapy	ALL, AML	Age Sex Weight Height Type of leukemia Time points for evaluation Pharmacological cancer treatment (anthracycline agents) Accumulated dose of anthracycline Comorbidities (e.g., pulmonary hypertension, systemic arterial hypertension) Echocardiographic parameters (e.g., LVEF Teichholz method, LVEF Simpson method, FS, TAPSE, GLS) Cardiac biomarkers (BNP, troponin I, troponin T)	Cardiac dysfunction related to cancer therapy, LVEF, electrocardiographic abnormalities, including impulse generation disturbances and repolarization disorders; pericardial effusion, valvular insufficiency, systemic arterial hypertension, pulmonary hypertension	Early-Onset	Anthracycline doses >150 mg/m^2^ were the main risk factor for early cardiotoxicity, affecting 17.9% of patients, especially in high-risk ALL and AML. Echocardiographic measures (LVEF, GLS) predicted cardiotoxicity, while biomarkers (troponin, BNP) did not. Pulmonary and systemic hypertension increased risk. Early GLS changes helped detect risk before symptoms, and ECG abnormalities did not correlate with cardiac dysfunction.
63	Abrahão et al. [87]/2020	Quantitative study (population-based cohort study)	To estimate the cumulative incidence and investigate the main predictors of late effects among adolescent and young adult (AYA) survivors of AML	AML	Race/ethnicity Sex Age at diagnosis Year of diagnosis Type of leukemia Cancer treatment exposures (radiotherapy, HSC) Pharmacological cancer treatment (chemotherapy agents) Neighborhood socioeconomic status Health insurance Long-term complications of cancer treatments (e.g., cardiovascular, respiratory, renal, liver/pancreas, endocrine, neurologic)	Hypertension, ischemic heart disease, cardiomyopathy, HF	Late-Onset	Cardiovascular (18.6%), endocrine (26.1%), and respiratory (6.6%) diseases were common in AML survivors. HSCT doubled the risk of late effects, including heart disease. Non-favorable risk AML, minority ethnicity, and lower socioeconomic status were linked to higher rates of complications.
64	Saussele et al. [88]/2020	Qualitative study (narrative review, expert consensus report)	To summarize current evidence regarding the efficacy and cardiovascular safety of Ponatinib in Philadelphia chromosome-positive Acute Leukemia and to provide recommendations for CV risk management	Ph+ ALL	Hypertension Systolic and diastolic blood pressure Comorbidities (e.g., hyperlipidemia, diabetes mellitus, chronic kidney disease, obesity) History of smoking Pre-existing cardiovascular conditions (e.g., family history of coronary disease) Existing cardiovascular disease conditions (e.g., peripheral artery disease, AOEs, severe hypertension, acute myocardial infarction, AF, acute coronary syndrome, transient ischemic attack) Response parameters (e.g., major hematologic response, major cytogenetic response, complete cytogenetic response, major molecular response) AOEs Venous thromboembolic events Adverse event or serious adverse event Pharmacological treatments (statin therapy) Electrocardiographic parameters Ankle brachial index Stress ECG/alternative stress test Monitoring frequency Treatment adjustments (prospective dose reduction, use of TKIs) Lipid profile (LDL-C, HDL-C, triglycerides, total cholesterol) Fasting glucose HbA1c Biochemical parameters (potassium, aminotransferases) Renal parameters (creatinine, glomerular filtration rate, urine microalbumin, urine protein)	AOEs, VTE, MI, Stroke, peripheral artery disease, hypertension	During Treatment to Early-Onset	Ponatinib was highly effective in treating Ph+ ALL, particularly in patients with the T315I mutation. Cardiovascular risk was dose-dependent, and dose reduction (from 45 mg to 30 mg or 15 mg) lowered CV risk while maintaining efficacy. Patients with pre-existing CV risk factors (hypertension, diabetes, hyperlipidemia) had a higher incidence of CV complications. Close collaboration between hematologists and cardiologists was recommended to manage CV risk while using Ponatinib.
65	Orvain et al. [89]/2020	Quantitative study (multicenter prospective study-GRAALL-2005 controlled trial)	To assess the efficacy and safety of thrombotic prophylaxis in adult ALL patients receiving L-asparaginase therapy, with a focus on fibrinogen supplementation, antithrombin supplementation, fresh frozen plasma, and heparin	ALL	Age Sex BMI History of smoking Contraception Comorbidities Type of leukemia CNS involvement Blood count parameters (WBC, HGB, PLT) Coagulation profile (prothrombin time, antithrombin, fibrinogen, procoagulant state) Poor early PB blast clearance Higher circulating tissue factor Central venous line placement Pharmacological cancer treatment (L-asparaginase treatment, fibrinogen supplementation, heparin prophylaxis)	VTE, DVT, pulmonary embolism, cerebral venous thrombosis	During Treatment to Early-Onset	L-asparaginase significantly raised VTE risk, especially in patients with low antithrombin. Fibrinogen and Antithrombin supplementation did not reduce risk and may have increased thrombosis. Heparin prophylaxis, particularly unfractionated Heparin, was linked to higher VTE rates. Central venous lines, older age, high hemoglobin, and obesity were independent VTE risk factors. Cerebral venous thrombosis cases were severe, often requiring intensive care.
66	Ociepa et al. [90]/2020	Quantitative study (cross-sectional Study)	To assess the cardiovascular risk in childhood survivors of ALL using carotid intima-media thickness as a potential early indicator of subclinical CVD	ALL	Age Age at diagnosis Time since diagnosis Sex Length of follow-up BMI Systolic and diastolic blood pressure Lipid profile (LDL-C, HDL-C, triglycerides, total cholesterol) Existing cardiovascular disease conditions (e.g., arterial hypertension, endothelial dysfunction, vascular tone abnormalities) Carotid intima-media thickness parameters	Arterial hypertension, endothelial dysfunction, possible atherosclerosis development, cardiovascular risk in adulthood	Late-Onset	Carotid Intima-Media Thickness was not a sensitive early marker of cardiovascular risk in childhood ALL survivors, showing no significant difference from controls or between hypertensive and normotensive survivors. Although hypertension was more common in this group, Carotid Intima-Media Thickness did not correlate strongly with BP, unlike in other pediatric conditions. Endothelial dysfunction and lipid profile changes may have been more relevant for early cardiovascular risk detection.
67	Neuendorff et al. [91]/2020	Qualitative study (narrative review)	To review the prevalence of anthracycline-related LVD in patients with AML, particularly older adults, and discuss risk factors, assessment methods, management strategies, and alternative treatments	AML	Age Sex Existing cardiovascular disease conditions (e.g., arterial hypertension, chronic heart failure, CAD, peripheral artery disease) Comorbidities (chronic renal insufficiency, frailty) Pharmacological cancer treatment (anthracycline agents) Cumulative dose of anthracycline agents Cytogenetic risk factors (P450 oxidoreductase SNPs) Cancer treatment exposures (radiotherapy, prior or concomitant radiation therapy, concomitant chemotherapy with alkylating or antimicrotubular agents) Pre-existing cardiovascular conditions Infections during treatment	Acute cardiotoxicity, chronic cardiotoxicity, acute HF, HF, arrhythmias, Myocarditis, subclinical LVEF decline, myocardial fibrosis	During Treatment to Late-Onset	Anthracyclines caused dose-dependent chronic cardiotoxicity in older AML patients, often leading to anthracycline-related LVD, HF, LVEF decline, and fibrosis. Risk was higher with preexisting heart conditions. Anthracycline-related LVD usually developed within a year and had poor recovery if not managed early. Alternatives like hypomethylating agents, venetoclax, and FLT3 inhibitors could be considered for high-risk patients.
68	Leerink et al. [92]/2020	Qualitative study (state-of-the-art review)	To review the prevalence, risk factors, prevention strategies, and risk prediction models for cardiac disease in childhood cancer survivors, as well as the methods used for risk prediction, prevention, and surveillance.	Acute Leukemia	Age at diagnosis Sex Cancer treatment exposures (radiotherapy, HSCT, chest radiation) Pharmacological cancer treatment (anthracyclines, mitoxantrone, cyclophosphamide) Comorbidities (e.g., diabetes, dyslipidemia, obesity)	HF, CAD, valvular heart disease, pericardial disease, arrhythmias, myocardial fibrosis, hypertension, obesity, and metabolic syndrome	Late-Onset	Anthracyclines, chest radiotherapy, and mitoxantrone (10× more cardiotoxic) were key cardiac risk factors in childhood cancer survivors. Traditional CV risks (hypertension, obesity, dyslipidemia) worsened late-onset heart disease. HF and CAD were major non-cancer killers. Preventive strategies like dexrazoxane and liposomal anthracyclines were promising, though their long-term efficacy was still being studied.
69	Jamal and Khaled [93]/2020	Qualitative study (narrative review)	To analyze the cardiovascular complications of CAR-T cell therapy, explore its mechanisms, and suggest approaches for clinical management	ALL	Cytokine release syndrome Existing cardiovascular disease conditions (e.g., hypotension, vasodilation) Pre-existing cardiovascular conditions	Cytokine release syndrome-induced cardiotoxicity, hypotension, tachycardia, cardiogenic shock, LV dysfunction, HF, arrhythmias, myocardial dysfunction, endothelial dysfunction, capillary leak syndrome	During Treatment to Early-Onset	Cardiotoxicity from cytokine release syndrome impaired vascular and heart function, often causing reversible LV dysfunction like stress cardiomyopathy. Pre-existing CVD increased risk, leading to more severe complications. Management strategies included fluid resuscitation, vasopressors, tocilizumab (IL-6 inhibitor), and cardio-oncology consultation.
70	Herrmann [94]/2020	Qualitative study (narrative review)	To examine the cardiotoxic effects of cancer treatments, including chemotherapy, targeted therapies, immunotherapies, and radiation therapy; mechanisms of cardiotoxicity, risk factors, and preventive strategies	ALL	Types of cancer therapy-related cardiomyopathy Risk stratification by therapy type Cancer treatment exposures (radiation therapy) Pharmacological cancer treatment (conventional chemotherapy, targeted therapies, immunotherapies) Diagnostic approaches (e.g., echocardiography, cardiac MRI, multigated acquisition scan, CT coronary angiography) Cardiac biomarkers (e.g., troponins, BNP, NT-proBNP) Thyroid function Inflammatory markers (e.g., IL-6, TNF-α) Catecholamines ECG abnormalities Types of targeted cancer Therapies Management of treatment (e.g., use of β-blockers, ACE inhibitors, angiotensin receptor blockers, spironolactone, anti-inflammatory and immunosuppressive therapy) Types of prevention (e.g., screening for comorbidities, exercise, cardiovascular medications, dose reduction in radiation therapy) Types of anticoagulant regimens in cancer therapy Leading cardiovascular toxic effects cancer treatments/exposures Existing cardiovascular disease conditions (e.g., arrhythmia, AF, QTc, prolongation, supraventricular tachycardia) Pharmacokinetic interactions (drug interactions, FDA black box warnings)	Cardiomyopathy, HF, arrhythmias, AF, ventricular tachycardia, hypertension, QT prolongation, sudden cardiac death, pericarditis, pericardial effusion, CAD, ischemia, myocarditis, thromboembolism, vascular toxicity	During Treatment to Late-Onset	Cancer therapies significantly impacted cardiovascular health, with newer treatments (targeted therapy, immunotherapy) presenting unique toxicity profiles. AF was an emerging major cardiac issue in cancer patients due to novel therapies. The combination of anthracyclines and targeted therapies (trastuzumab) increased the risk of irreversible cardiomyopathy. Early detection and monitoring (echocardiography, biomarkers like cardiac troponins) helped mitigate severe cardiac complications.
71	Giudice et al. [95]/2020	Qualitative study (narrative review)	To analyze and summarize the cardiotoxic effects associated with targeted therapies used in hematology, particularly focusing on novel drugs like BTK inhibitors, PI3K inhibitors, IDH inhibitors, and monoclonal antibodies	AML	Age Hypertension Pre-existing cardiovascular conditions (ischemic heart disease) Comorbidities (e.g., diabetes, hypercholesterolemia) Pharmacological cancer treatment (anthracyclines, HER2-targeted therapies, other chemotherapies include 5-Fluorouracil, cisplatin) Cumulative dose of cardiotoxic drugs Genetic polymorphisms affecting drug metabolism (e.g., CBR3, HER2/ERBB2 variants) Monoclonal antibody names Target antigen or receptor of the monoclonal antibody Prolonged pharmacological exposure	Hypertension, QT prolongation, arrhythmias, HF, CHF, LVD, acute coronary syndromes, MI, AOEs, VTE, pericardial and pleural effusion	During Treatment to Late-Onset	Pre-existing cardiovascular conditions and cumulative drug exposure significantly heightened the risk of cardiotoxicity in targeted leukemia treatments, affecting both survival and quality of life. Ponatinib and Nilotinib were linked to a high rate of AOEs, while BTK and PI3K inhibitors were strongly associated with QT prolongation and arrhythmias. FLT3 inhibitors and hypomethylating agents carried a moderate risk, causing QT prolongation and hypertension. Preventive strategies, such as ACE inhibitors, β-blockers, and statins, may have helped reduce cardiotoxicity severity.
72	Gavriilaki et al. [96]/2020	Quantitative study (observational case–control study)	To assess vascular injury and pro-coagulant activity in allo-HCT survivors without existing allo-HCT complications or relapses, using circulating microvesicles as markers	ALL, AML	Age BMI History of smoking Type of leukemia Systolic and diastolic blood pressure Heart rate Lipid profile (LDL-C, HDL-C, triglycerides, total cholesterol) Blood glucose History of cardiotoxic cancer treatments Cellular biomarkers (platelet microvesicles, erythrocyte microvesicles, endothelial microvesicles) Disease status Cancer treatment exposures (allo-HCT, myeloablative conditioning, TBI-based conditioning) Transplant complications (e.g., Acute GVHD grade II–IV, extensive chronic GVHD) Transplant procedure parameters (donor type, transplant characteristics)	Endothelial dysfunction, increased thrombotic risk, hypertension, dyslipidemia, increased risk of CVD independent of traditional risk factors	Late-Onset	Endothelial dysfunction and thrombotic risk persisted long after allo-HCT, regardless of traditional cardiovascular risk factors. Myeloablative conditioning increased endothelial microvesicles, indicating ongoing vascular injury, while the history of thrombotic microangiopathy was associated with elevated erythrocyte microvesicles, reflecting a lasting thrombo-inflammatory effect. The correlation between platelet and endothelial microvesicles reinforced the link between endothelial injury and pro-coagulant activity. Since traditional risk factors (hypertension, dyslipidemia) did not fully explain the elevated cardiovascular risk, novel biomarkers like microvesicles were essential for improved risk stratification in allo-HCT survivors.
73	Cook and Litzow [97]/2020	Qualitative study (narrative review)	To summarize emerging toxicities associated with modern immunotherapeutic and targeted treatment strategies for ALL and to provide insights into supportive care measures that can optimize patient outcomes	ALL	History of smoking Hypertension Pharmacological cancer treatment (blinatumomab, TKIs, CAR-T cell therapy) CTCAE 5.0 cytokine release syndrome grading Management of cytokine release syndrome American Society for Transplantation and Cellular Therapies Cytokine Release Syndrome Grading Immune effector cell-associated neurotoxicity syndrome grading Multi-domain neurologic evaluation Types of TKIs Comprehensive cardiovascular risk assessment (e.g., Framingham, Systematic Coronary Risk Evaluation, Reynolds, American College of Cardiology/American Heart Association) Comorbidities (e.g., hyperlipidemia, diabetes mellitus, obesity) Management of asparaginase toxicities	Vascular toxicity, vascular occlusive events, platelet dysfunction, QTc prolongation, arrhythmias, pulmonary artery hypertension	During Treatment to Late-Onset	Ponatinib and nilotinib carried a significant risk of vascular complications; comprehensive cardiovascular screening was recommended before initiating therapy. Dasatinib was associated with QTc prolongation, pleural effusion, and platelet dysfunction, and routine ECG and bleeding risk assessment were recommended.
74	Bhatia [98]/2020	Qualitative study (narrative review)	To investigate the genetic factors contributing to anthracycline-related cardiomyopathy in cancer survivors, focusing on the role of genetic variants in modifying the risk	ALL, AML	Age at anthracycline exposure Type of leukemia Sex Cancer treatment exposures (chest radiation) Pre-existing cardiovascular conditions Pharmacological cancer treatment (concurrent use of cyclophosphamide, paclitaxel, and trastuzumab)	Cardiomyopathy, HF, LVEF	Early-Onset to Late-Onset	A dose-dependent relationship existed between anthracycline exposure and cardiomyopathy risk. Genetic susceptibility played a role, with variants in genes e.g., SLC28A3, affecting cardiotoxicity risk. Concurrent risk factors (age at exposure, female sex, chest radiation, cardiovascular risk factors) exacerbated the risk. Potential preventive strategies included genetic screening and personalized monitoring.
75	Mohamed et al. [99]/2020	Quantitative study (retrospective observational cohort study)	To evaluate the association between a leukemia diagnosis and clinical outcomes of AMI, focusing on clinical characteristics, management strategies, and outcomes in leukemia patients	ALL, AML	Age Sex Race/ethnicity Admission details Socioeconomic status Type of leukemia Alcohol consumption History of smoking Hospital characteristics Comorbidities (e.g., dyslipidemia, thrombocytopenia, diabetes, hypothyroidism, liver disease) Management strategies (use of assist device or intra-aortic balloon pump, shock, coronary angiography, percutaneous coronary intervention, coronary artery bypass grafting)	Cardiac tamponade, hemopericardium, coronary dissection, shock	Early-Onset to Late-Onset	Leukemia patients, particularly those with AML, experienced significantly worse cardiovascular outcomes after AMI, with a fourfold increase in mortality and a threefold increase in major Acute cardiovascular and cerebrovascular events risk. They were also less likely to undergo coronary angiography (48.5% vs. 64.5%) and Percutaneous Coronary Intervention (28.2% vs. 42.9%). These findings highlighted the need for a multidisciplinary approach involving both cardiologists and hematology-oncologists to optimize care and outcomes.

Abbreviations: ALL: acute lymphoblastic leukemia; B-ALL: B-cell Acute Lymphoblastic Leukemia; T-ALL: T-cell Acute Lymphoblastic Leukemia; AML: Acute Myeloid Leukemia; APL: Acute Promyelocytic Leukemia (a subtype of AML); Ph+ ALL: Philadelphia Chromosome-positive ALL; FLT3-mutated AML: FMS-like Tyrosine Kinase 3-Mutated AML; NPM1: Nucleophosmin 1; BCR::ABL1: Breakpoint Cluster Region::ABL proto-oncogene 1 (fusion gene); FLT3: FMS-like Tyrosine kinase 3; FLT3-ITD: FLT3-Internal Tandem Duplication; FLT3-TKD: FLT3-Tyrosine Kinase Domain; IDH: Isocitrate Dehydrogenase; CHIP: Clonal Hematopoiesis of Indeterminate Potential; T315I: Threonine-to-Isoleucine mutation at position 315 (in BCR::ABL1); SNP: Single Nucleotide Polymorphism; H3K27ac: Histone 3 K27 acetylation; allo-HCT/Allo-HSCT/allo-SCT: Allogeneic Hematopoietic (Stem) Cell Transplantation; HSCT/HCT/HST/SCT: Hematopoietic (Stem) Cell Transplantation; BMT: Bone Marrow Transplantation; TBI: Total Body Irradiation; GVHD: Graft-versus-Host Disease; CV: Cardiovascular; CAD: Coronary Artery Disease; AF: Atrial Fibrillation; HF/CHF: (Congestive) Heart Failure; CVD/CVDs: Cardiovascular Disease(s); DVT: Deep Vein Thrombosis; DD: Diastolic Dysfunction; LVD: Left Ventricular Dysfunction; LVDD: Left Ventricular Diastolic Dysfunction; LVSD: Left Ventricular Systolic Dysfunction; CRS: Cytokine Release Syndrome; MI/AMI: (Acute) Myocardial Infarction; AOE/AOEs: Arterial Occlusive Event(s); ATE/ATEs: Arterial Thromboembolism/Event(s); VTE: Venous Thromboembolism; MACE/MACCE: Major (Acute) Adverse (Cardiovascular/Cerebrovascular) Events; Non-MACE: Non-Major Adverse Cardiac Events; VAs: Ventricular Arrhythmias; VT: Ventricular Tachycardia; ECG: Electrocardiogram; CMRI/MRI: (Cardiac) Magnetic Resonance Imaging; CT/PET-CT: Computed Tomography/(Positron Emission Tomography) CT; LV: Left Ventricle; LVEF/EF: (Left Ventricular) Ejection Fraction; LVFS/FS: (Left Ventricular) Fractional Shortening; GLS/LV GLS/RV GLS: (Left/Right Ventricular) Global Longitudinal Strain; RV: Right Ventricle; TAPSE: Tricuspid Annular Plane Systolic Excursion; EDV: End-Diastolic Volume; ESV: End-Systolic Volume; E/A ratio: Early diastolic filling velocity/Atrial contraction velocity ratio; QTc: Corrected QT Interval; WBC: White Blood Cell count; RBC: Red Blood Cell count; HGB: Hemoglobin; PLT: Platelet count; ALT: Alanine Aminotransferase; AST: Aspartate Aminotransferase; APTT: Activated Partial Thromboplastin Time; HbA1C: Hemoglobin A1C; BNP: B-type Natriuretic Peptide; NT-proBNP: N-terminal pro-B-type Natriuretic Peptide; cTnT/hs-cTnT: (High-sensitivity) cardiac troponin T; Hs-cTnI: High-Sensitivity Cardiac Troponin I; ST2: Suppression of Tumorigenicity 2; TNF-α: Tumor Necrosis Factor-α; IL-1, IL-6, IL-10, IL-18: Interleukins Family; ACE inhibitors/ACE inhibitor: Angiotensin-Converting Enzyme inhibitors; TKIs: Tyrosine Kinase Inhibitors; BTK inhibitors: Bruton’s Tyrosine Kinase inhibitors; CAR-T: Chimeric Antigen Receptor T-cell therapy; AZD5991: Investigational MCL-1 inhibitor (AstraZeneca); CNS: Central Nervous System; BMI: Body Mass Index; Gy: Gray (unit of radiation dose); BM: Bone Marrow; CTCAE/CTCAE 5.0: Common Terminology Criteria for Adverse Events (version 5.0).

## Supplementary Materials

The following supporting information can be downloaded at: https://www.mdpi.com/article/10.3390/cancers17172777/s1, Supplementary S1: Search Strategy; Supplementary S2: Quality Assessment and Risk of Bias.

## Abbreviations

The following abbreviations are used in this manuscript:

ACS	Acute Coronary Syndrome
AF	Atrial Fibrillation
AL	Acute Leukemia
ALL	Acute Lymphoblastic Leukemia
Allo-HCT/allo-HSCT/allo-SCT	Allogeneic Hematopoietic (Stem) Cell Transplantation
AML	Acute Myeloid Leukemia
AMSTAR 2	A Measurement Tool to Assess Systematic Reviews (Version 2)
AOEs	Acute Onset Events
APL	Acute Promyelocytic Leukemia
ASCVD	Atherosclerotic Cardiovascular Disease
ATE	Arterial Thromboembolism
BMI	Body Mass Index
BNP	B-type Natriuretic Peptide
CAD	Coronary Artery Disease
CEL/HES	Chronic Eosinophilic Leukemia/Hypereosinophilic Syndrome
CHIP	Clonal Hematopoiesis of Indeterminate Potential
CML	Chronic Myeloid Leukemia
CMML	Chronic Myelomonocytic Leukemia
CNL	Chronic Neutrophilic Leukemia
CRS	Cytokine Release Syndrome
CTCAE/CTCAE 5.0	Common Terminology Criteria for Adverse Events (version 5.0)
cTnT/hs-cTnT	(High-sensitivity) Cardiac Troponin T
CVD	Cardiovascular Disease
DD	Diastolic Dysfunction
DVT	Deep Vein Thrombosis
ECG	Electrocardiogram
ET	Essential Thrombocythemia
GLS	Global Longitudinal Strain
GVHD	Graft-versus-Host Disease
HSCT	Hematopoietic Stem Cell Transplantation
Hs-cTnI	High-Sensitivity Cardiac Troponin
IDH	Isocitrate Dehydrogenase
JBI	Joanna Briggs Institute
JMML	Juvenile Myelomonocytic Leukemia
LVD	Left Ventricular Dysfunction
LVEF	Left Ventricular Ejection Fraction
MCD	Mast Cell Disease
MDS/MPN	Myelodysplastic/Myeloproliferative Neoplasms
MDS	Myelodysplastic Syndromes
MPAL	Mixed-Phenotype Acute Leukemia
MPNs	Myeloproliferative Neoplasms
NIH	National Institutes of Health (Quality Assessment Tool)
NT-proBNP	N-terminal pro-B-type Natriuretic Peptide
PAD	Peripheral Artery Disease
PE	Pulmonary Embolism
Ph+ ALL	Philadelphia Chromosome-Positive Acute Lymphoblastic Leukemia
PMF	Primary Myelofibrosis
PRISMA	Preferred Reporting Items for Systematic Reviews and Meta-Analyses
PV	Polycythemia Vera
RoB 2	Cochrane Risk-of-Bias Tool for Randomized Trials (Version 2)
ROBINS-I	Risk Of Bias In Non-randomized Studies-of Interventions
ROBIS	Risk Of Bias In Systematic Reviews
SANRA	Scale for the Assessment of Narrative Review Articles
t-AML	Therapy-Related Acute Myeloid Leukemia
TAPSE	Tricuspid Annular Plane Systolic Excursion
TBI	Total Body Irradiation
TIA	Transient Ischemic Attack
TKI	Tyrosine Kinase Inhibitors
TMI	Total Marrow Irradiation
VAs	Ventricular Arrhythmias
VTE	Venous Thromboembolism
