# Supplementary material for: Determining Risk Factors Associated with Cardiovascular Complications in Patients with Acute Leukemia: A Systematic Review
Source: Cancers (Basel). 2025 Aug 26;17(17):2777. doi: 10.3390/cancers17172777 (PMC12427311; doi:10.3390/cancers17172777)
Supplement: Supplementary file 1 [file cancers-17-02777-s001.zip › Supplementary S1 - Search Strategy.pdf]

Supplementary S1 - Search Strategy

Main Concepts	MeSH Terms	Entry Terms/ key words from related articles	Search Strategy	With Mesh Terms
Acute Leukemia	“Leukemia, Myeloid, Acute”<br>“Leukemia, Lymphoid”	“Acute Myeloid Leukemia”<br>“Acute Myelogenous Leukemia”<br>“Acute Myelocytic Leukemia”<br>“Acute Myeloblastic Leukemia”<br>“Acute Nonlymphocytic Leukemia”<br>“Acute Nonlymphoblastic Leukemia”<br>“Precursor Cell Lymphoblastic Leukemia Lymphoma”<br>“Lymphoblastic Leukemia”<br>“Acute Lymphocytic Leukemia”<br>“Acute Lymphoid Leukemia”<br>“Acute Lymphoblastic Leukemia”<br>“Mixed Phenotype Acute Leukemia”	("Leukemia, Myeloid, Acute" OR "Leukemia, Lymphoid" OR "Acute Myeloid Leukemia" OR "Acute Myelogenous Leukemia" OR "Acute Myelocytic Leukemia" OR "Acute Myeloblastic Leukemia" OR "Acute Nonlymphocytic Leukemia" OR "Acute Nonlymphoblastic Leukemia" OR "Precursor Cell Lymphoblastic Leukemia Lymphoma" OR "Lymphoblastic Leukemia" OR "Acute Lymphocytic Leukemia" OR "Acute Lymphoid Leukemia" OR "Acute Lymphoblastic Leukemia" OR “Mixed Phenotype Acute Leukemia”)	("Leukemia, Myeloid, Acute" [MeSH Terms] OR "Leukemia, Lymphoid" [MeSH Terms] OR "Acute Myeloid Leukemia" OR "Acute Myelogenous Leukemia" OR "Acute Myelocytic Leukemia" OR "Acute Myeloblastic Leukemia" OR "Acute Nonlymphocytic Leukemia" OR "Acute Nonlymphoblastic Leukemia" OR "Precursor Cell Lymphoblastic Leukemia Lymphoma" OR "Lymphoblastic Leukemia" OR "Acute Lymphocytic Leukemia" OR "Acute Lymphoid Leukemia" OR "Acute Lymphoblastic Leukemia" OR “Mixed Phenotype Acute Leukemia”)
Heart Disease Risk Factors	“Heart Disease Risk Factors”	“Cardiovascular Risk Factors”<br>“Cardiovascular Risk”	("Heart Disease Risk Factors" OR "Cardiovascular Risk Factors" OR "Cardiovascular Risk")	("Heart Disease Risk Factors" [MeSH Terms] OR "Cardiovascular Risk Factors" OR "Cardiovascular Risk")

Main Strategy

(("Leukemia, Myeloid, Acute" OR "Leukemia, Lymphoid" OR "Acute Myeloid Leukemia" OR "Acute Myelogenous Leukemia" OR "Acute Myelocytic Leukemia" OR "Acute Myeloblastic Leukemia" OR "Acute Nonlymphocytic Leukemia" OR "Acute Nonlymphoblastic Leukemia" OR "Precursor Cell Lymphoblastic Leukemia Lymphoma" OR "Lymphoblastic Leukemia" OR "Acute Lymphocytic Leukemia" OR "Acute Lymphoid Leukemia" OR "Acute Lymphoblastic Leukemia" OR “Mixed Phenotype Acute Leukemia”) AND ("Heart Disease Risk Factors" OR "Cardiovascular Risk Factors" OR "Cardiovascular Risk"))

The Search Results

Database	Strategy	Results	Limit Language and Time
**PubMed**	(("Leukemia, Myeloid, Acute" [MeSH Terms] OR "Leukemia, Myeloid, Acute"[Title/Abstract] OR "Leukemia, Lymphoid" [MeSH Terms] OR "Leukemia, Lymphoid"[Title/Abstract] OR "Acute Myeloid Leukemia"[Title/Abstract] OR "Acute Myelogenous Leukemia"[Title/Abstract] OR "Acute Myelocytic Leukemia"[Title/Abstract] OR "Acute Myeloblastic Leukemia"[Title/Abstract] OR "Acute Nonlymphocytic Leukemia"[Title/Abstract] OR "Acute Nonlymphoblastic Leukemia"[Title/Abstract] OR "Precursor Cell Lymphoblastic Leukemia Lymphoma"[Title/Abstract] OR "Lymphoblastic Leukemia"[Title/Abstract] OR "Acute Lymphocytic Leukemia"[Title/Abstract] OR "Acute Lymphoid Leukemia"[Title/Abstract] OR "Acute Lymphoblastic Leukemia"[Title/Abstract] OR "Mixed Phenotype Acute Leukemia"[Title/Abstract]) AND ("Heart Disease Risk Factors" [MeSH Terms] OR "Heart Disease Risk Factors"[Title/Abstract] OR "Cardiovascular Risk Factors"[Title/Abstract] OR "Cardiovascular Risk"[Title/Abstract]))	**66**	**45**
**IEEE Xplore**	(("Leukemia, Myeloid, Acute" OR "Leukemia, Lymphoid" OR "Acute Myeloid Leukemia" OR "Acute Myelogenous Leukemia" OR "Acute Myelocytic Leukemia" OR "Acute Myeloblastic Leukemia" OR "Acute Nonlymphocytic Leukemia" OR "Acute Nonlymphoblastic Leukemia" OR "Precursor Cell Lymphoblastic Leukemia Lymphoma" OR "Lymphoblastic Leukemia" OR "Acute Lymphocytic Leukemia" OR "Acute Lymphoid Leukemia" OR "Acute Lymphoblastic Leukemia" OR “Mixed Phenotype Acute Leukemia”) AND ("Heart Disease Risk Factors" OR "Cardiovascular Risk Factors" OR "Cardiovascular Risk"))	**0**	**0**
**Scopus**	TITLE-ABS-KEY((("Leukemia, Myeloid, Acute" OR "Leukemia, Lymphoid" OR "Acute Myeloid Leukemia" OR "Acute Myelogenous Leukemia" OR "Acute Myelocytic Leukemia" OR "Acute Myeloblastic Leukemia" OR "Acute	**374**	**260**

	Nonlymphocytic Leukemia" OR "Acute Nonlymphoblastic Leukemia" OR "Precursor Cell Lymphoblastic Leukemia Lymphoma" OR "Lymphoblastic Leukemia" OR "Acute Lymphocytic Leukemia" OR "Acute Lymphoid Leukemia" OR "Acute Lymphoblastic Leukemia" OR “Mixed Phenotype Acute Leukemia”) AND ("Heart Disease Risk Factors" OR "Cardiovascular Risk Factors" OR "Cardiovascular Risk")))		
**Cochrane Library**	("Leukemia, Myeloid, Acute" OR "Leukemia, Lymphoid" OR "Acute Myeloid Leukemia" OR "Acute Myelogenous Leukemia" OR "Acute Myelocytic Leukemia" OR "Acute Myeloblastic Leukemia" OR "Acute Nonlymphocytic Leukemia" OR "Acute Nonlymphoblastic Leukemia" OR "Precursor Cell Lymphoblastic Leukemia Lymphoma" OR "Lymphoblastic Leukemia" OR "Acute Lymphocytic Leukemia" OR "Acute Lymphoid Leukemia" OR "Acute Lymphoblastic Leukemia" OR “Mixed Phenotype Acute Leukemia”) AND ("Heart Disease Risk Factors" OR "Cardiovascular Risk Factors" OR "Cardiovascular Risk")) in Title Abstract Keyword	**11**	**8**
**WOS**	((TI=((("Leukemia, Myeloid, Acute" OR "Leukemia, Lymphoid" OR "Acute Myeloid Leukemia" OR "Acute Myelogenous Leukemia" OR "Acute Myelocytic Leukemia" OR "Acute Myeloblastic Leukemia" OR "Acute Nonlymphocytic Leukemia" OR "Acute Nonlymphoblastic Leukemia" OR "Precursor Cell Lymphoblastic Leukemia Lymphoma" OR "Lymphoblastic Leukemia" OR "Acute Lymphocytic Leukemia" OR "Acute Lymphoid Leukemia" OR "Acute Lymphoblastic Leukemia" OR "Mixed Phenotype Acute Leukemia") AND ("Heart Disease Risk Factors" OR "Cardiovascular Risk Factors" OR "Cardiovascular Risk")))) OR AB=((("Leukemia, Myeloid, Acute" OR "Leukemia, Lymphoid" OR "Acute Myeloid Leukemia" OR "Acute Myelogenous Leukemia" OR "Acute Myelocytic Leukemia" OR "Acute Myeloblastic Leukemia" OR "Acute Nonlymphocytic Leukemia" OR "Acute Nonlymphoblastic Leukemia" OR "Precursor Cell Lymphoblastic Leukemia Lymphoma" OR "Lymphoblastic Leukemia" OR "Acute Lymphocytic Leukemia" OR "Acute Lymphoid Leukemia" OR "Acute Lymphoblastic Leukemia" OR "Mixed Phenotype Acute Leukemia") AND ("Heart Disease Risk Factors" OR "Cardiovascular Risk Factors" OR "Cardiovascular Risk")))) OR KP=((("Leukemia, Myeloid, Acute" OR "Leukemia, Lymphoid" OR "Acute Myeloid Leukemia" OR "Acute Myelogenous Leukemia" OR "Acute Myelocytic Leukemia" OR "Acute Myeloblastic Leukemia" OR "Acute Nonlymphocytic Leukemia" OR "Acute Nonlymphoblastic Leukemia" OR "Precursor Cell Lymphoblastic Leukemia Lymphoma" OR "Lymphoblastic Leukemia" OR "Acute Lymphocytic Leukemia" OR "Acute Lymphoid Leukemia" OR "Acute Lymphoblastic Leukemia" OR "Mixed Phenotype Acute Leukemia") AND ("Heart Disease Risk Factors" OR "Cardiovascular Risk Factors" OR "Cardiovascular Risk"))))	**114**	**74**
**ProQuest**	title((("Leukemia, Myeloid, Acute" OR "Leukemia, Lymphoid" OR "Acute Myeloid Leukemia" OR "Acute Myelogenous Leukemia" OR "Acute	**9**	**8**

	Myelocytic Leukemia" OR "Acute Myeloblastic Leukemia" OR "Acute Nonlymphocytic Leukemia" OR "Acute Nonlymphoblastic Leukemia" OR "Precursor Cell Lymphoblastic Leukemia Lymphoma" OR "Lymphoblastic Leukemia" OR "Acute Lymphocytic Leukemia" OR "Acute Lymphoid Leukemia" OR "Acute Lymphoblastic Leukemia" OR “Mixed Phenotype Acute Leukemia”) AND ("Heart Disease Risk Factors" OR "Cardiovascular Risk Factors" OR "Cardiovascular Risk"))) OR abstract((("Leukemia, Myeloid, Acute" OR "Leukemia, Lymphoid" OR "Acute Myeloid Leukemia" OR "Acute Myelogenous Leukemia" OR "Acute Myelocytic Leukemia" OR "Acute Myeloblastic Leukemia" OR "Acute Nonlymphocytic Leukemia" OR "Acute Nonlymphoblastic Leukemia" OR "Precursor Cell Lymphoblastic Leukemia Lymphoma" OR "Lymphoblastic Leukemia" OR "Acute Lymphocytic Leukemia" OR "Acute Lymphoid Leukemia" OR "Acute Lymphoblastic Leukemia" OR “Mixed Phenotype Acute Leukemia”) AND ("Heart Disease Risk Factors" OR "Cardiovascular Risk Factors" OR "Cardiovascular Risk"))) OR mainsubject((("Leukemia, Myeloid, Acute" OR "Leukemia, Lymphoid" OR "Acute Myeloid Leukemia" OR "Acute Myelogenous Leukemia" OR "Acute Myelocytic Leukemia" OR "Acute Myeloblastic Leukemia" OR "Acute Nonlymphocytic Leukemia" OR "Acute Nonlymphoblastic Leukemia" OR "Precursor Cell Lymphoblastic Leukemia Lymphoma" OR "Lymphoblastic Leukemia" OR "Acute Lymphocytic Leukemia" OR "Acute Lymphoid Leukemia" OR "Acute Lymphoblastic Leukemia" OR “Mixed Phenotype Acute Leukemia”) AND ("Heart Disease Risk Factors" OR "Cardiovascular Risk Factors" OR "Cardiovascular Risk")))		
**Scholar**	("Acute Leukemia" AND "Cardiovascular Risk Factor")	**429**	**285**
