# Supplementary material for: Determining Risk Factors Associated with Cardiovascular Complications in Patients with Acute Leukemia: A Systematic Review
Source: Cancers (Basel). 2025 Aug 26;17(17):2777. doi: 10.3390/cancers17172777 (PMC12427311; doi:10.3390/cancers17172777)
Supplement: Supplementary file 1 [file cancers-17-02777-s001.zip › Supplementary S2 - Quality Assessment and ROB.pdf]

Supplementary S2 - Quality Assessment and Risk of Bias

JBI Checklist for Quality Assessment of RCTs

Author (year of publication)	Q1	Q2	Q3	Q4	Q5	Q6	Q7	Q8	Q9	Q10	Q11	Q12	Q13	Total Points	Score	Quality
Desai et al. (1) / 2024	N	N/A	Y	N	N	U	N	Y	Y	Y	N/A	Y	Y	6	0.46	Moderate
Kantarjian et al.(2) / 2023	N	N/A	N/A	N	N	N/A	U	N/A	Y	Y	N/A	Y	Y	4	0.30	Poor
El Amrousy et al. (3) / 2021	Y	Y	Y	U	U	Y	Y	Y	Y	Y	Y	Y	Y	11	0.84	Excellent
Chow et al. (4) / 2021	Y	U	Y	N	N	Y	Y	Y	Y	Y	Y	Y	Y	10	0.76	Good
Orvain et al. (5) / 2020	Y	U	Y	U	U	Y	U	Y	Y	Y	Y	Y	Y	9	0.69	Good

Q1: Was true randomization used for assignment of participants to treatment groups? Q2: Was allocation to treatment groups concealed? Q3: Were treatment groups similar at the baseline? Q4: Were participants blind to treatment assignment? Q5: Were those delivering the treatment blind to treatment assignment? Q6: Were treatment groups treated identically other than the intervention of interest? Q7: Were outcome assessors blind to treatment assignment? Q8: Were outcomes measured in the same way for treatment groups? Q9: Were outcomes measured in a reliable way? Q10: Was follow up complete and if not, were differences between groups in terms of their follow up adequately described and analysed? Q11: Were participants analysed in the groups to which they were randomized? Q12: Was appropriate statistical analysis used? Q13: Was the trial design appropriate and any deviations from the standard RCT design (individual randomization, parallel groups) accounted for in the conduct and analysis of the trial?

Abbreviations: C= Can't tell; Y= Yes; N= No; U =Unclear N/A = Not Applicable; Quality Items: Poor (≤ 0.40), Moderate (0.41 to 0.59), Good (0.60 to 0.79), or Excellent (≥ 0.80)

JBI Checklist for Quality Assessment of Cohort Studies

Author (year of publication)	Q1	Q2	Q3	Q4	Q5	Q6	Q7	Q8	Q9	Q10	Q11	Total Points	Score	Quality
Udin et al. (6) / 2024	N	Y	Y	Y	Y	U	Y	Y	U	U	Y	7	0.63	Moderate
Sameer et al. (7) / 2024	Y	Y	Y	Y	Y	U	Y	Y	Y	N	Y	9	0.81	High

Author (year of publication)	Q1	Q2	Q3	Q4	Q5	Q6	Q7	Q8	Q9	Q10	Q11	Total Points	Score	Quality
Onoue et al. (8) / 2024	N	Y	Y	Y	Y	Y	Y	Y	U	U	Y	8	0.72	Moderate
Salas et al. (9) / 2024	Y	Y	Y	Y	Y	Y	Y	Y	U	N/A	Y	9	0.81	High
Ma et al. (10) / 2024	Y	Y	Y	Y	Y	Y	Y	U	U	N/A	Y	8	0.72	Moderate
Hellman and Chaireti (11) / 2024	Y	Y	Y	Y	U	N	Y	Y	N	U	Y	7	0.63	Moderate
Hammoud et al. (12) / 2024	N	Y	Y	Y	Y	Y	Y	U	Y	Y	Y	9	0.81	High
Kępski et al. (13) / 2024	Y	Y	Y	Y	Y	U	Y	Y	U	N/A	Y	8	0.72	Moderate
Diaz et al. (14) / 2024	Y	N/A	Y	Y	Y	Y	Y	Y	U	U	Y	8	0.72	Moderate
Mitrovic et al. (15) / 2023	Y	Y	Y	Y	U	Y	Y	Y	U	N/A	Y	8	0.72	Moderate
Boluda et al. (16) /2023	Y	Y	Y	Y	Y	U	Y	Y	U	U	Y	8	0.72	Moderate
Baum et al. (17)/ 2023	Y	Y	Y	Y	Y	U	Y	Y	N	U	Y	8	0.72	Moderate
Zhou et al. (18) / 2023	Y	Y	Y	Y	Y	Y	Y	Y	U	N/A	Y	9	0.81	High
Auberle et al.(19) / 2023	U	N/A	Y	Y	Y	Y	Y	Y	Y	Y	Y	9	0.81	High
Alpman et al. (20) / 2023	U	N/A	Y	Y	N	Y	Y	Y	Y	U	Y	7	0.63	Moderate
Ketterl et al. (21) / 2023	Y	Y	Y	Y	Y	U	Y	Y	N	N	Y	8	0.72	Moderate
Januzzi et al. (22) / 2022	Y	N/A	Y	Y	Y	Y	Y	Y	U	U	Y	8	0.72	Moderate
Terada et al. (23) / 2022	Y	Y	Y	Y	U	Y	Y	Y	U	N/A	Y	8	0.72	Moderate
Xiao et al. (24)/ 2022	N	Y	Y	Y	Y	U	Y	Y	U	N/A	Y	7	0.63	Moderate
Calvillo-Argüelles et al. (25) / 2022	N	Y	Y	Y	Y	U	Y	Y	N	N	Y	7	0.63	Moderate
Petrykey et al.(26) / 2021	Y	Y	Y	Y	Y	Y	Y	Y	U	N/A	Y	9	0.81	High
Lubas et al. (27) / 2021	Y	Y	Y	Y	Y	U	Y	Y	N	U	Y	8	0.72	Moderate
Oka et al. (28) / 2021	Y	N/A	Y	Y	N	Y	Y	Y	Y	N	Y	8	0.72	Moderate
Gangaraju et al. (29)/ 2021	N	N	Y	Y	Y	Y	Y	Y	N	U	Y	7	0.63	Moderate

Author (year of publication)	Q1	Q2	Q3	Q4	Q5	Q6	Q7	Q8	Q9	Q10	Q11	Total Points	Score	Quality
Linares Ballesteros et al. (30) / 2021	Y	Y	Y	Y	U	Y	Y	Y	N	U	Y	8	0.72	Moderate
Duléry et al.(31) / 2021	N	Y	Y	Y	Y	U	Y	Y	U	N/A	Y	7	0.63	Moderate
Mohamed et al. (32) / 2020	Y	Y	Y	Y	Y	Y	Y	Y	Y	N/A	Y	10	0.90	High
Abrahão et al. (33)/ 2020	Y	Y	Y	Y	Y	Y	Y	Y	U	U	Y	9	0.81	High

Q1: Were the two groups similar and recruited from the same population? Q2: Were the exposures measured similarly to assign people to both exposed and unexposed groups? Q3: Was the exposure measured in a valid and reliable way? Q4: Were confounding factors identified? Q5: Were strategies to deal with confounding factors stated? Q6: Were the groups/participants free of the outcome at the start of the study (or at the moment of exposure)? Q7: Were the outcomes measured in a valid and reliable way? Q8: Was the follow up time reported and sufficient to be long enough for outcomes to occur? Q9: Was follow up complete, and if not, were the reasons to loss to follow up described and explored? Q10: Were strategies to address incomplete follow up utilized? Q11: Was appropriate statistical analysis used?
Abbreviations: Y= Yes; N= No; U =Unclear N/A = Not Applicable; Quality Items: Low (<0.6), Moderate (0.6 to 0.8), High (0.8>)

AMSTAR 2 Checklist for Quality Assessment of Systematic reviews

Author (year of publication)	Q1	Q2	Q3	Q4	Q5	Q6	Q7	Q8	Q9	Q10	Q11	Q12	Q13	Q14	Q15	Q16	Total Points	Score
Bertrand et al. (34) / 2023	Y	Y	Y	Y	PY	Y	Y	Y	Y	N	NMAC	NMAC	Y	Y	NMAC	Y	12	0.75
Luo et al. (35) / 2022	PY	N	Y	Y	N	N	N	Y	N	N	NMAC	NMAC	N	PY	NMAC	Y	5	0.31

Q1: Did the research questions and inclusion criteria for the review include the components of PICO? Q2: Did the report contain an explicit statement that the review methods were established prior to the conduct of the review and did the report justify any significant deviations from the protocol? Q3: Did the review authors explain their selection of the study designs for inclusion in the review? Q4: Did the review authors use a comprehensive literature search strategy? Q5: Did the review authors perform study selection in duplicate? Q6: Did the review authors perform data extraction in duplicate? Q7: Did the review authors provide a list of excluded studies and justify the exclusions? Q8: Did the review authors describe the included studies in adequate detail? Q9: Did the review authors use a satisfactory technique for assessing the risk of bias (RoB) in individual studies? Q10: Did the review authors report on the sources of funding for the studies included in the review? Q11: If meta-analysis was performed, did the review authors use appropriate methods for statistical combination of results? Q12: If meta-analysis was performed, did the review authors assess the potential impact of

RoB in individual studies on the results? Q13: Did the review authors account for RoB in individual studies when interpreting/discussing the results? Q14: Did the review authors provide a satisfactory explanation for, and discussion of, any heterogeneity observed in the results? Q15: If they performed quantitative synthesis, did the review authors carry out an adequate investigation of publication bias (small study bias) and discuss its likely impact? Q16: Did the review authors report any potential sources of conflict of interest, including any funding they received for conducting the review?
Abbreviations: Y= Yes; N= No; PY= Partial Yes; NMAC= No Meta analysis Conducted

JBI Critical Appraisal Checklist for Case Control Studies

Author (year of publication)	Q1	Q2	Q3	Q4	Q5	Q6	Q7	Q8	Q9	Q10	Total Points	Score	Quality
Poudel et al. (36) / 2024	Y	Y	Y	Y	Y	Y	Y	Y	Y	Y	10	1	High
Muggeo et al. (37) /2022	N	Y	U	Y	N/A	Y	Y	Y	Y	Y	7	0.7	Moderate
Cornelissen et al. (38) / 2022	Y	Y	Y	Y	Y	Y	Y	Y	Y	Y	10	1	High
Gavriilaki et al. (39)/ 2020	Y	Y	U	Y	Y	Y	Y	Y	Y	Y	9	0.9	High

Q1: Were the groups comparable other than the presence of disease in cases or the absence of disease in controls? Q2: Were cases and controls matched appropriately? Q3: Were the same criteria used for identification of cases and controls? Q4: Was exposure measured in a standard, valid and reliable way? Q5: Was exposure measured in the same way for cases and controls? Q6: Were confounding factors identified? Q7: Were strategies to deal with confounding factors stated? Q8: Were outcomes assessed in a standard, valid and reliable way for cases and controls? Q9: Was the exposure period of interest long enough to be meaningful? Q10: Was appropriate statistical analysis used?
Abbreviations: Y= Yes; N= No; U =Unclear N/A = Not Applicable; Quality Items: Low (<0.6), Moderate (0.6 to 0.8), High (0.8>)

JBI Critical Appraisal Checklist for Case Series

Author (year of publication)	Q1	Q2	Q3	Q4	Q5	Q6	Q7	Q8	Q9	Q10	Total Points	Score	Quality
Fazal et al. (40)/ 2023	Y	Y	Y	U	U	Y	Y	Y	Y	N/A	7	0.7	Moderate

Q1: Were there clear criteria for inclusion in the case series? Q2: Was the condition measured in a standard, reliable way for all participants included in the case series? Q3: Were valid methods used for identification of the condition for all participants included in the case series? Q4: Did the case series

have consecutive inclusion of participants? Q5: Did the case series have complete inclusion of participants? Q6: Was there clear reporting of the demographics of the participants in the study? Q7: Was there clear reporting of clinical information of the participants? Q8: Were the outcomes or follow up results of cases clearly reported? Q9: Was there clear reporting of the presenting site(s)/clinic(s) demographic information? Q10: Was statistical analysis appropriate?
Abbreviations: Y= Yes; N= No; U =Unclear N/A = Not Applicable; Quality Items: Low (<0.6), Moderate (0.6 to 0.8), High (0.8>)

JBI Critical Appraisal Checklist for Analytical Cross-Sectional Studies

Author (year of publication)	Q1	Q2	Q3	Q4	Q5	Q6	Q7	Q8	Total Points	Score	Quality
Fernández-Avilés et al. (41) / 2024	Y	Y	Y	Y	Y	Y	Y	Y	8	1	High
Kundavaram et al. (42) / 2024	Y	Y	Y	Y	Y	N	Y	Y	7	0.87	High
Rique et al. (43) / 2024	Y	Y	Y	Y	Y	Y	Y	Y	8	1	High
Heredia et al. (44) / 2023	Y	Y	Y	Y	Y	Y	Y	Y	8	1	High
Lipshultz et al.(45) / 2022	Y	Y	N/A	Y	Y	Y	Y	Y	7	0.87	High
Gonzalez-Manzanares et al. (46) / 2022	Y	Y	Y	Y	Y	Y	Y	Y	8	1	High
Ociepa et al. (47) / 2020	Y	Y	Y	Y	Y	Y	Y	Y	8	1	High

Q1: Were the criteria for inclusion in the sample clearly defined? Q2: Were the study subjects and the setting described in detail? Q3: Was the exposure measured in a valid and reliable way? Q4: Were objective, standard criteria used for measurement of the condition? Q5: Were confounding factors identified? Q6: Were strategies to deal with confounding factors stated? Q7: Were the outcomes measured in a valid and reliable way? Q8; Was appropriate statistical analysis used?
Abbreviations: Y= Yes; N= No; U =Unclear N/A = Not Applicable; Quality Items: Low (<0.6), Moderate (0.6 to 0.8), High (0.8>)

Q-Genie Checklist

Author (year of publication)	Q1	Q2	Q3	Q4	Q5	Q6	Q7	Q8	Q9	Q10	Q11	Total Points	Score	Quality
Wang et al. (48) / 2023	7	6	6	7	6	5	6	7	7	6	7	70	70/77	Good

Q1: Was a scientific rationale or hypothesis clearly presented? Q2: Were outcomes (e.g. disease status or trait) well defined and appropriately sampled? Q3: Were controls well defined and matched appropriately? Q4: Was the genotyping accurate and technically well executed? Q5: Were

genotyping methods standardized and blinded? Q6: Did authors report and address potential biases like selection, attrition, or time-lag? Q7: Was sample size justified and adequate? Was power analysis reported? Q8: Was the analysis plan pre-defined and completely reported? Q9: Were confounders controlled and multiple testing addressed? Q10: Were assumptions tested, e.g. haplotype inference, relatedness, ethnicity? Q11: Were the conclusions well-supported by data and methods?
Abbreviations: 1 = Poor, 7 = Excellent; Poor quality (0–35), Moderate quality (36–45), Good quality (45 -77)

SANRA Checklist for Quality Assessment of Narrative Review Articles

Author (year of publication)	Justification of the article's importance for the readership	Statement of concrete aims or formulation of questions	Description of the literature search	Referencing	Scientific reasoning	Appropriate presentation of data	Score	Quality
Hammoud et al. (49) /2024	2	2	1	2	2	2	11	High
Spannbauer and Bergler-Klein (50) / 2024	2	2	1	2	2	2	11	High
Roganovic et al. (51)/ 2024	2	2	1	2	2	2	11	High
Liu et al. (52) / 2024	2	2	1	2	2	1	10	High
Dogliotti et al. (53) / 2024	2	2	1	2	2	2	11	High
Barachini et al. (54) / 2024	2	2	1	2	2	2	11	High
Gawlik et al. (55) / 2023	2	2	1	2	2	2	11	High
Berisha et al.(56) / 2023	2	2	1	2	2	2	11	High
Bottinor and Chow (57) / 2022	2	2	1	2	2	2	11	High

Author (year of publication)	Justification of the article's importance for the readership	Statement of concrete aims or formulation of questions	Description of the literature search	Referencing	Scientific reasoning	Appropriate presentation of data	Score	Quality
Arnán Sangerman et al. (58)/ 2022	2	2	2	2	2	2	12	High
Chianca et al. (59) / 2022	2	2	1	2	2	2	11	High
Perpinia et al. (60)/ 2022	2	2	1	2	2	2	11	High
Hoeben et al. (61) / 2021	2	2	1	2	2	2	11	High
Diesch-Furlanetto et al. (62) / 2021	2	2	1	2	2	2	11	High
Chen et al. (63) / 2021	2	2	1	2	2	2	11	High
Burns et al. (64) / 2021	2	2	1	2	2	2	11	High
Lazăr et al. (65) / 2021	2	2	1	2	2	2	11	High
kamarajuet al. (66)/ 2021	2	2	1	2	2	1	10	High
Neuendorff et al. (67) / 2020	2	2	1	2	2	1	10	High
Leerink et al. (68) / 2020	2	2	2	2	2	2	12	High
Saussele et al. (69) / 2020	2	2	1	2	2	2	11	High
Jamal and Khaled (70) / 2020	2	2	1	2	2	2	11	High
Herrmann (71) / 2020	2	2	1	2	2	2	11	High
Giudice et al. (72) / 2020	2	2	1	2	2	2	11	High
Cook and Litzow (73)/ 2020	2	2	1	2	2	2	11	High
Bhatia (74) / 2020	2	2	1	2	2	2	11	High

Quality Items: Low (<7), Moderate (7 to 9), High (10 to 12)

NIH Quality Assessment Tool for Before-After (Pre-Post) Studies with No Control Group

Author (year of publication)	Q1	Q2	Q3	Q4	Q5	Q6	Q7	Q8	Q9	Q10	Q11	Q12	Total Points	Score	Quality
Puła et al. (75) / 2024	Y	Y	Y	CD	Y	Y	Y	N/R	CD	CD	Y	N	7	0.58	Low

Q1: Was the study question or objective clearly stated? Q2: Were eligibility/selection criteria for the study population prespecified and clearly described? Q3: Were the participants in the study representative of those who would be eligible for the test/service/intervention in the general or clinical population of interest? Q4: Were all eligible participants that met the prespecified entry criteria enrolled? Q5: Was the sample size sufficiently large to provide confidence in the findings? Q6: Was the test/service/intervention clearly described and delivered consistently across the study population? Q7: Were the outcome measures prespecified, clearly defined, valid, reliable, and assessed consistently across all study participants? Q8: Were the people assessing the outcomes blinded to the participants' exposures/interventions? Q9: Was the loss to follow-up after baseline 20% or less? Were those lost to follow-up accounted for in the analysis? Q10: Did the statistical methods examine changes in outcome measures from before to after the intervention? Were statistical tests done that provided p values for the pre-to-post changes? Q11: Were outcome measures of interest taken multiple times before the intervention and multiple times after the intervention (i.e., did they use an interrupted time-series design)? IQ12: f the intervention was conducted at a group level (e.g., a whole hospital, a community, etc.) did the statistical analysis take into account the use of individual-level data to determine effects at the group level?

Abbreviations: Y= Yes; N= No; CD= Cannot Determine; N/A = Not Applicable; N/R= Not Reported; Quality Items: Low (<0.6), Moderate (0.6 to 0.8), High (0.8>)

Risk of Bias Assessment for RCTs Using RoB 2.0 Tool

	Bias Domain					
**Author (year of publication)**	**Bias arising from the randomization process**	**Bias due to deviations from intended interventions**	**Bias due to missing outcome data**	**Bias in measurement of the outcome**	**Bias in selection of the reported result**	**Overall bias**
Desai et al. (1) / 2024	High	Low	Some concerns	Low	Low	Some concerns
Kantarjian et al.(2) / 2023	High	Low	Low	Some concerns	Some concerns	Some concerns
El Amrousy et al. (3) / 2021	Low	Some concerns	Low	Low	Low	Low
Chow et al. (4) / 2021	Low	Some concerns	Low	Low	Low	Low
Orvain et al. (5) / 2020	Low	Some concerns	Low	Low	Low	Low

Risk of Bias Assessment for Systematic reviews using ROBIS

	Bias Domain				
**Author (year of publication)**	**Eligibility Criteria**	**Identification and Selection of Studies**	**Data Collection and Study Appraisal**	**Synthesis and Findings**	**Overall bias**
Bertrand et al. (34) / 2023	Low	Some concerns	Some concerns	Some concerns	Some concerns
Luo et al. (35) / 2022	Some concerns	Some concerns	Some concerns	Some concerns	Some concerns

Risk of Bias Assessment for genetic association studies

	Bias Domain							
**Author (year of publication)**	**Phenotype definition**	**Genotyping misclassification**	**Selection of sample**	**Confounding by ethnic origin**	**Multiple testing**	**Relatedness**	**Treatment effects**	**Overall bias**
Wang et al. (48) / 2023	Low	Low	Moderate	Low	Low	Low	Moderate	Low

Risk of Bias Assessment for Qualitative Review Studies and Qualitative Studies using Triangulation Checklist

**Author (year of publication)**	**Data Triangulation**	**Methodological Triangulation**	**Theoretical Triangulation**	**Investigator Triangulation**	**Source Triangulation**	**Overall bias**
Spannbauer and Bergler-Klein (50) / 2024	Moderate	Low	Low	Low	Moderate	Moderate
Roganovic et al. (51)/ 2024	Low	Low	Moderate	Low	Low	Low
Liu et al. (52) / 2024	Low	Moderate	Low	N/A	Low	Low
Hammoud et al. (49) /2024	Moderate	Moderate	Low	Low	Low	Moderate
Dogliotti et al. (53) / 2024	Low	Moderate	Low	Low	Low	Low
Barachini et al. (54) / 2024	Low	Moderate	Low	N/A	Low	Low
Gawlik et al. (55) / 2023	Low	Moderate	Low	N/A	Moderate	Moderate
Berisha et al.(56) / 2023	Low	Moderate	Low	N/A	Low	Low
Perpinia et al. (60)/ 2022	Moderate	Low	Moderate	Moderate	Moderate	Moderate
Chianca et al. (59) / 2022	Low	Low	Moderate	Low	Low	Low
Bottinor and Chow (57) / 2022	Low	Low	Low	Low	Low	Low
Arnán Sangerman et al. (58)/ 2022	Low	Low	Low	Moderate	Low	Low

Author (year of publication)	Data Triangulation	Methodological Triangulation	Theoretical Triangulation	Investigator Triangulation	Source Triangulation	Overall bias
Lazăr et al. (65) / 2021	Low	Moderate	Low	Low	Low	Low
kamarajuet al. (66)/ 2021	Low	Low	Moderate	Low	Moderate	Moderate
Hoeben et al. (61) / 2021	Moderate	Low	Moderate	Low	Low	Moderate
Diesch-Furlanetto et al. (62) / 2021	Moderate	Low	Moderate	Low	Moderate	Moderate
Chen et al. (63) / 2021	Low	Low	Moderate	Low	Moderate	Moderate
Burns et al. (64) / 2021	Moderate	Moderate	Low	Moderate	Moderate	Moderate
Saussele et al. (69) / 2020	Moderate	Moderate	Low	High	Moderate	Moderate
Neuendorff et al. (67) / 2020	Moderate	Moderate	Low	Low	Moderate	Moderate
Leerink et al. (68) / 2020	Low	Moderate	Low	Low	Low	Low
Jamal and Khaled (70) / 2020	Moderate	Low	Moderate	Low	Moderate	Moderate
Herrmann (71) / 2020	Low	Moderate	Low	Moderate	Low	Moderate
Giudice et al. (72) / 2020	Moderate	Moderate	Low	Low	Moderate	Moderate
Cook and Litzow (73)/ 2020	Low	Moderate	Moderate	Low	Low	Moderate
Bhatia (74) / 2020	Moderate	Moderate	Low	Moderate	Moderate	Moderate

Risk of Bias Assessment for non-randomized interventions using ROBINS-I

Author (year of publication)	Bias due to confounding	Bias in selection of participants into the study	Bias in classification of interventions	Bias due to deviations from intended interventions	Bias due to missing data	Bias in measurement of outcomes	Bias in selection of the reported result	Overall bias
Udin et al. (6) / 2024	Moderate	Serious	Low	Moderate	Moderate	Low	Moderate	Moderate
Puła et al. (75) / 2024	Moderate	Moderate	Low	Low	Moderate	Low	Moderate	Moderate
Poudel et al. (36) / 2024	Low	Low	Low	Low	Moderate	Low	Low	Low
Salas et al. (9) / 2024	Moderate	Low	Low	Low	Moderate	Moderate	Low	Moderate
Fernández-Avilés et al. (41) / 2024	Low	Moderate	Low	Low	Low	Low	Low	Low

Author (year of publication)	Bias due to confounding	Bias in selection of participants into the study	Bias in classification of interventions	Bias due to deviations from intended interventions	Bias due to missing data	Bias in measurement of outcomes	Bias in selection of the reported result	Overall bias
Kundavaram et al. (42) / 2024	Moderate	Moderate	Low	Low	Low	Low	Moderate	Moderate
Ma et al. (10) / 2024	Low	Moderate	Low	Null	Moderate	Low	Moderate	Moderate
Rique et al. (43) / 2024	Moderate	Moderate	Low	Low	Low	Low	Moderate	Moderate
Sameer et al. (7) / 2024	Low	Moderate	Low	Low	Moderate	Low	Moderate	Moderate
Onoue et al. (8) / 2024	Moderate	Moderate	Low	Low	Moderate	Low	Low	Moderate
Hellman and Chaireti (11) / 2024	Moderate	Moderate	Low	Low	Moderate	Low	Low	Moderate
Hammoud et al. (12) / 2024	Low	Moderate	Low	Low	Moderate	Low	Low	Low
Diaz et al. (14) / 2024	Low	Moderate	Low	Low	Moderate	Low	Low	Low
Kępski et al. (13) / 2024	Moderate	Moderate	Low	Low	Moderate	Low	Moderate	Moderate
Mitrovic et al. (15) / 2023	Moderate	Low	Low	Null	Moderate	Low	Low	Moderate
Boluda et al . (16) /2023	Moderate	Low	Low	Moderate	Moderate	Low	Low	Moderate
Ketterl et al. (21) / 2023	Low	Moderate	Low	Low	Moderate	Low	Low	Low
Heredia et al. (44) / 2023	Low	Moderate	Low	Low	Low	Low	Low	Low
Baum et al. (17)/ 2023	Moderate	Moderate	Low	Low	Moderate	Moderate	Low	Moderate
Auberle et al.(19) / 2023	Moderate	Moderate	Low	Null	Low	Low	Low	Moderate
Zhou et al. (18) / 2023	Moderate	Low	Low	Low	Moderate	Low	Moderate	Moderate
Fazal et al. (40)/ 2023	Moderate	Serious	Low	Moderate	Low	Low	Moderate	Moderate
Alpman et al. (20) / 2023	Moderate	Moderate	Low	Low	Moderate	Low	Moderate	Moderate
Januzzi et al. (22) / 2022	Moderate	Low	Low	Moderate	Low	Low	Low	Low
Calvillo-Argüelles et al. (25) / 2022	Moderate	Moderate	Low	Low	Moderate	Low	Low	Moderate
Gonzalez-Manzanares et al. (46) / 2022	Low	Moderate	Low	Low	Low	Low	Low	Low
Lipshultz et al.(45) / 2022	Moderate	Serious	Low	Low	Moderate	Low	Low	Moderate
Muggeo et al. (37) /2022	Moderate	Moderate	Low	Low	Low	Low	Low	Low
Terada et al. (23) / 2022	Moderate	Moderate	Low	Low	Moderate	Low	Low	Moderate
Xiao et al. (24)/ 2022	Moderate	Moderate	Low	Low	Moderate	Low	Moderate	Moderate

Author (year of publication)	Bias due to confounding	Bias in selection of participants into the study	Bias in classification of interventions	Bias due to deviations from intended interventions	Bias due to missing data	Bias in measurement of outcomes	Bias in selection of the reported result	Overall bias
Cornelissen et al. (38) / 2022	Moderate	Moderate	Low	Null	Moderate	Low	Low	Moderate
Petrykey et al.(26) / 2021	Low	Moderate	Low	Low	Moderate	Low	Moderate	Moderate
Linares Ballesteros et al. (30) / 2021	Moderate	Low	Low	Low	Moderate	Low	Low	Low
Lubas et al. (27) / 2021	Moderate	Moderate	Low	Low	Moderate	Low	Moderate	Moderate
Oka et al. (28) / 2021	Moderate	Low	Low	Low	Moderate	Low	Low	Low
Gangaraju et al. (29)/ 2021	Moderate	Serious	Low	Low	Moderate	Moderate	Low	Moderate
Duléry et al.(31) / 2021	Moderate	Moderate	Low	Low	Moderate	Low	Low	Moderate
Mohamed et al. (32) / 2020	Moderate	Low	Low	Low	Low	Low	Low	Low
Abrahão et al. (33)/ 2020	Moderate	Low	Low	Low	Moderate	Moderate	Low	Moderate
Gavriilaki et al. (39)/ 2020	Low	Moderate	Low	Low	Low	Low	Moderate	Low
Ociepa et al. (47) / 2020	Moderate	Moderate	Low	Low	Low	Low	Low	Moderate

Abbreviations: Low = Low risk of bias; Moderate = Moderate risk of bias; Serious = Serious risk of bias; Critical = Critical risk of bias; Null = No Information.

References

1. Desai P, Lonial S, Cashen A, Kamdar M, Flinn I, O'Brien S, et al. A Phase 1 First-in-Human Study of the MCL-1 Inhibitor AZD5991 in Patients with Relapsed/Refractory Hematologic Malignancies. Clin Cancer Res. 2024;30(21):4844-55.
2. Kantarjian H, Short NJ, Jain N, Sasaki K, Huang X, Haddad FG, et al. Frontline combination of ponatinib and hyper-CVAD in Philadelphia chromosome-positive acute lymphoblastic leukemia: 80-months follow-up results. Am J Hematol. 2023;98(3):493-501.
3. El Amrousy D, El-Afify D, Khedr R, Ibrahim AM. Omega 3 fatty acids can reduce early doxorubicin-induced cardiotoxicity in children with acute lymphoblastic leukemia. Pediatr Blood Cancer. 2022;69(7):e29496.
4. Chow EJ, Doody DR, Di C, Armenian SH, Baker KS, Bricker JB, et al. Feasibility of a behavioral intervention using mobile health applications to reduce cardiovascular risk factors in cancer survivors: a pilot randomized controlled trial. J Cancer Surviv. 2021;15(4):554-63.

5. Orvain C, Balsat M, Tavernier E, Marolleau JP, Pabst T, Chevallier P, et al. Thromboembolism prophylaxis in adult patients with acute lymphoblastic leukemia treated in the GRAALL-2005 study. Blood. 2020;136(3):328-38.
6. Udin MH, Sunder SS, Nepali S, Kattel S, Abdelradi A, Doyle ST, et al. Differential cardiac impacts of hematological malignancies and solid tumors: a histopathological and biomarker study. Cardiooncology. 2024;10(1):90.
7. Sameer S, N P, Kuppusamy S, Adole PS, Kayal S. Cardiac Autonomic and Endothelial Function in Acute Lymphoblastic Leukaemia Patients Immediately After Chemotherapy and at the Three-Month Follow-up. Cureus. 2024;16(2):e55108.
8. Onoue T, Matthews AH, Vakilpour A, Kang Y, Lefebvre B, Smith AM, et al. Cardiotoxicity of venetoclax in patients with acute myeloid leukemia: comparison with anthracyclines. Cardio-Oncology. 2024;10(1):75.
9. Salas MQ, Cascos E, López-García A, Pérez E, Baile-González M, Martín Rodríguez C, et al. Cardiac events after allo-HCT in patients with acute myeloid leukemia. Blood Adv. 2024;8(21):5497-509.
10. Ma L, Wang Q, Li X, Shang Y, Zhang N, Wu J, et al. Development of a risk assessment model for cardiac injury in patients newly diagnosed with acute myeloid leukemia based on a multicenter, real-world analysis in China. BMC Cancer. 2024;24(1):132.
11. Hellman J, Chaireti R. Incidence and Risk Factors for Arterial Thrombosis in Patients with Acute Leukemia and Lymphoid Malignancies: A Retrospective Single-Center Study. Cancers (Basel). 2024;16(14).
12. Hammoud RA, Liu Q, Dixon SB, Onerup A, Mulrooney DA, Huang IC, et al. The burden of cardiovascular disease and risk for subsequent major adverse cardiovascular events in survivors of childhood cancer: a prospective, longitudinal analysis from the St Jude Lifetime Cohort Study. Lancet Oncol. 2024;25(6):811-22.
13. Kępski J, Szmit S, Lech-Marańda E. Time Relationship between the Occurrence of a Thromboembolic Event and the Diagnosis of Hematological Malignancies. Cancers (Basel). 2024;16(18).
14. Diaz ANR, Hurtado GP, Manzano AAA, Keyes MJ, Turissini C, Choudhary A, et al. Sex Differences in the Development of Anthracycline-Associated Heart Failure. J Card Fail. 2024;30(7):907-14.
15. Mitrovic M, Pantic N, Sabljic N, Bukumiric Z, Virijevic M, Pravdic Z, et al. Arterial Thrombosis in Patients with Acute Myeloid Leukemia: Incidence and Risk Factors. Cancers (Basel). 2023;15(11).
16. Boluda B, Solana-Altabella A, Cano I, Martínez-Cuadrón D, Acuña-Cruz E, Torres-Miñana L, et al. Incidence and Risk Factors for Development of Cardiac Toxicity in Adult Patients with Newly Diagnosed Acute Myeloid Leukemia. Cancers (Basel). 2023;15(8).
17. Baum J, Lax H, Lehmann N, Merkel-Jens A, Beelen DW, Jöckel KH, Dührsen U. Preventive health care in blood cancer survivors: results from the ABC study. J Cancer Res Clin Oncol. 2023;149(13):11531-40.
18. Zhou X, Weng Y, Jiang T, Ou W, Zhang N, Dong Q, Tang X. Influencing factors of anthracycline-induced subclinical cardiotoxicity in acute leukemia patients. BMC Cancer. 2023;23(1):976.
19. Auberle C, Lenihan D, Gao F, Cashen A. Late cardiac events after allogeneic stem cell transplant: incidence, risk factors, and impact on overall survival. Cardiooncology. 2023;9(1):1.
20. Alpman MS, Toth I, Langebäck A, Broberg AM, Herold N. Incidence and surveillance of acute cardiovascular toxicities in paediatric acute lymphoblastic leukaemia: A retrospective population-based single-centre cohort study. EJC Paediatric Oncology. 2023;2:100020.
21. Ketterl TG, Chow EJ, Koves IH, Goodman P, Leisenring WM, Ballard S, et al. Impact of Hematopoietic Cell Transplantation on Cardiovascular Risk Factors and Insulin Sensitivity. Transplant Cell Ther. 2024;30(2):243.e1-.e13.
22. Januzzi JL, Garasic JM, Kasner SE, McDonald V, Petrie MC, Seltzer J, et al. Retrospective analysis of arterial occlusive events in the PACE trial by an independent adjudication committee. J Hematol Oncol. 2022;15(1):1.

23. Terada CI, Onoue K, Fujii T, Itami H, Morita K, Uchiyama T, et al. Histopathological and epigenetic changes in myocardium associated with cancer therapy-related cardiac dysfunction. ESC Heart Fail. 2022;9(5):3031-43.
24. Xiao W, Ma L, Shang Y, Yang F, Tan Y, Chen G, et al. Cardiac-Related Lesions in Newly Diagnosed Patients With Acute Leukemia: A Chinese Population-Based Real-World Study. Front Med (Lausanne). 2022;9:844350.
25. Calvillo-Argüelles O, Schoffel A, Capo-Chichi JM, Abdel-Qadir H, Schuh A, Carrillo-Estrada M, et al. Cardiovascular Disease Among Patients With AML and CHIP-Related Mutations. JACC CardioOncol. 2022;4(1):38-49.
26. Petrykey K, Rezgui AM, Guern ML, Beaulieu P, St-Onge P, Drouin S, et al. Genetic factors in treatment-related cardiovascular complications in survivors of childhood acute lymphoblastic leukemia. Pharmacogenomics. 2021;22(14):885-901.
27. Lubas MM, Wang M, Jefferies JL, Ness KK, Ehrhardt MJ, Krull KR, et al. The Contribution of Stress and Distress to Cardiovascular Health in Adult Survivors of Childhood Cancer. Cancer Epidemiol Biomarkers Prev. 2021;30(2):286-94.
28. Oka T, Tada Y, Oboshi M, Kamada R, Yasui T, Shioyama W, et al. Serial Changes in Cardiac Strain and Contractility After Hematopoietic Stem Cell Transplantation in Patients with Hematologic Malignancies. Int Heart J. 2021;62(3):575-83.
29. Gangaraju R. Risk of Coronary Heart Disease in Blood or Marrow Transplant Survivors: The University of Alabama at Birmingham; 2021.
30. Linares Ballesteros A, Sanguino Lobo R, Villada Valencia JC, Arévalo Leal O, Plazas Hernández DC, Aponte Barrios N, Perdomo Ramírez I. Early-onset Cardiotoxicity assessment related to anthracycline in children with leukemia. A Prospective Study. Colomb Med (Cali). 2021;52(1):e2034542.
31. Duléry R, Mohty R, Labopin M, Sestili S, Malard F, Brissot E, et al. Early Cardiac Toxicity Associated With Post-Transplant Cyclophosphamide in Allogeneic Stem Cell Transplantation. JACC CardioOncol. 2021;3(2):250-9.
32. Mohamed MO, Lopez-Mattei JC, Parwani P, Iliescu CA, Bharadwaj A, Kim PY, et al. Management strategies and clinical outcomes of acute myocardial infarction in leukaemia patients: Nationwide insights from United States hospitalisations. Int J Clin Pract. 2020;74(5):e13476.
33. Abrahão R, Huynh JC, Benjamin DJ, Li QW, Winestone LE, Muffly L, Keegan THM. Chronic medical conditions and late effects after acute myeloid leukaemia in adolescents and young adults: a population-based study. International Journal of Epidemiology. 2021;50(2):663-74.
34. Bertrand É, Caru M, Harvey A, Dodin P, Jacquemet V, Curnier D. Cardiac electrical abnormalities in childhood acute lymphoblastic leukemia survivors: a systematic review. Cardiooncology. 2023;9(1):40.
35. Luo Z, Cheng J, Wang Y. Cardiac Infiltration as the First Manifestation of Acute Lymphoblastic Leukemia: A Systematic Review. Front Oncol. 2022;12:805981.
36. Poudel S, Shrestha H, Pan Y, Li Q, Li K, Im C, et al. Serum Proteins Predict Treatment-Related Cardiomyopathy Among Survivors of Childhood Cancer. JACC CardioOncol. 2025;7(1):56-67.
37. Muggeo P, Scicchitano P, Muggeo VMR, Novielli C, Giordano P, Ciccone MM, et al. Assessment of Cardiovascular Function in Childhood Leukemia Survivors: The Role of the Right Heart. Children (Basel). 2022;9(11).
38. Cornelissen LL, Kreuger AL, Caram-Deelder C, Huisman MV, Middelburg RA, Kerkhoffs JLH, et al. Association between cardiovascular risk factors and intracranial hemorrhage in patients with acute leukemia. Eur J Haematol. 2022;108(4):310-8.
39. Gavriilaki E, Sakellari I, Anyfanti P, Batsis I, Vardi A, Bousiou Z, et al. Assessment of Endothelial Injury and Pro-Coagulant Activity Using Circulating Microvesicles in Survivors of Allogeneic Hematopoietic Cell Transplantation. Int J Mol Sci. 2020;21(24).
40. Fazal M, Wei C, Chuy KL, Hussain K, Gomez SE, Ba SS, et al. Tyrosine kinase inhibitor-associated ventricular arrhythmias: a case series and review of literature. J Interv Card Electrophysiol. 2023;66(5):1165-75.
41. Fernández-Avilés C, González-Manzanares R, Ojeda S, Molina JR, Heredia G, Resúa A, et al. Diastolic function assessment with left atrial strain in long-term survivors of childhood acute lymphoblastic leukemia. Rev Esp Cardiol (Engl Ed). 2024;77(1):60-8.

42. Kundavaram R, Kumar A, Konnepati S, Yadav YS, Chaudhary NK, Malik S, Gogia P. Acute Ventricular Dysfunction After Doxorubicin-Based Induction Therapy for Pediatric Acute Lymphoblastic Leukemia. Cureus. 2024;16(12):e75720.
43. Rique A, Cautela J, Thuny F, Michel G, Ovaert C, El Louali F. Left Ventricular Longitudinal Strain Abnormalities in Childhood Exposure to Anthracycline Chemotherapy. Children (Basel). 2024;11(3).
44. Heredia G, Gonzalez-Manzanares R, Ojeda S, Molina JR, Fernandez-Aviles C, Hidalgo F, et al. Right Ventricular Function in Long-Term Survivors of Childhood Acute Lymphoblastic Leukemia: From the CTOXALL Study. Cancers (Basel). 2023;15(21).
45. Lipshultz ER, Chow EJ, Doody DR, Armenian SH, Asselin BL, Baker KS, et al. Cardiometabolic Risk in Childhood Cancer Survivors: A Report from the Children's Oncology Group. Cancer Epidemiol Biomarkers Prev. 2022;31(3):536-42.
46. Gonzalez-Manzanares R, Castillo JC, Molina JR, Ruiz-Ortiz M, Mesa D, Ojeda S, et al. Automated Global Longitudinal Strain Assessment in Long-Term Survivors of Childhood Acute Lymphoblastic Leukemia. Cancers (Basel). 2022;14(6).
47. Ociepa T, Posio W, Sawicki M, Urasiński T. CIMT does not identify early vascular changes in childhood acute lymphoblastic leukemia survivors. Adv Clin Exp Med. 2020;29(2):243-9.
48. Wang X, Singh P, Zhou L, Sharafeldin N, Landier W, Hageman L, et al. Genome-Wide Association Study Identifies ROBO2 as a Novel Susceptibility Gene for Anthracycline-Related Cardiomyopathy in Childhood Cancer Survivors. J Clin Oncol. 2023;41(9):1758-69.
49. Hammoud RA, Mulrooney DA, Rhea IB, Yu C, Johnson JN, Chow EJ, et al. Modifiable Cardiometabolic Risk Factors in Survivors of Childhood Cancer: JACC: CardioOncology State-of-the-Art Review. JACC CardioOncol. 2024;6(1):16-32.
50. Spannbauer A, Bergler-Klein J. Cardio-Oncology: A New Discipline in Medicine and Its Relevance to Hematology. Hamostaseologie. 2024;44(04):255-67.
51. Roganovic J, Haupt R, Bárdi E, Hjorth L, Michel G, Pavasovic V, et al. Late Adverse Effects after Treatment for Childhood Acute Leukemia. Acta Med Acad. 2024;53(1):59-80.
52. Liu X, Ge S, Zhang A. Pediatric Cardio-Oncology: Screening, Risk Stratification, and Prevention of Cardiotoxicity Associated with Anthracyclines. Children (Basel). 2024;11(7).
53. Dogliotti I, Levis M, Martin A, Bartoncini S, Felicetti F, Cavallin C, et al. Maintain Efficacy and Spare Toxicity: Traditional and New Radiation-Based Conditioning Regimens in Hematopoietic Stem Cell Transplantation. Cancers (Basel). 2024;16(5).
54. Barachini S, Buda G, Petrini I. Cardiovascular Toxicity of Antineoplastic Treatments in Hematological Diseases: Focus on Molecular Mechanisms to Improve Therapeutic Management. J Clin Med. 2024;13(6).
55. Gawlik M, Zimodro JM, Gąsecka A, Filipiak KJ, Szmit S. Cardiac Arrhythmias in Oncological Patients-Epidemiology, Risk Factors, and Management within the Context of the New ESC 2022 Guidelines. Curr Oncol Rep. 2023;25(10):1107-15.
56. Berisha A, Placci A, Piccaluga PP. Cardiotoxicity of Tyrosine Kinase Inhibitors in Philadelphia-Positive Leukemia Patients. Hemato. 2023;4(1):68-75.
57. Bottinor W, Chow EJ. Mitigating, monitoring, and managing long-term chemotherapy- and radiation-induced cardiac toxicity. Hematology Am Soc Hematol Educ Program. 2022;2022(1):251-8.
58. Arnán Sangerman M, Fernández Moreno A, García Quintana A, García-Vidal C, Olave Rubio MT, Del Mar Tormo Díaz M, et al. Practical tips for managing FLT3 mutated acute myeloid leukemia with midostaurin. Expert Rev Hematol. 2022;15(3):203-14.
59. Chianca M, Panichella G, Fabiani I, Giannoni A, L'Abbate S, Aimo A, et al. Bidirectional Relationship Between Cancer and Heart Failure: Insights on Circulating Biomarkers. Front Cardiovasc Med. 2022;9:936654.
60. Perpinia AS, Kadoglou N, Vardaka M, Gkortzolidis G, Karavidas A, Marinakis T, et al. Pharmaceutical Prevention and Management of Cardiotoxicity in Hematological Malignancies. Pharmaceuticals (Basel). 2022;15(8).

61. Hoeben BAW, Wong JYC, Fog LS, Losert C, Filippi AR, Bentzen SM, et al. Total Body Irradiation in Haematopoietic Stem Cell Transplantation for Paediatric Acute Lymphoblastic Leukaemia: Review of the Literature and Future Directions. Front Pediatr. 2021;9:774348.
62. Diesch-Furlanetto T, Gabriel M, Zajac-Spychala O, Cattoni A, Hoeben BAW, Balduzzi A. Late Effects After Haematopoietic Stem Cell Transplantation in ALL, Long-Term Follow-Up and Transition: A Step Into Adult Life. Front Pediatr. 2021;9:773895.
63. Chen DH, Tyebally S, Mallouppas M, Ghosh AK. CAR T Cell and BiTE Therapy—New Therapies, New Risks? Current Cardiovascular Risk Reports. 2020;15(1):1.
64. Burns EA, Gentille C, Trachtenberg B, Pingali SR, Anand K. Cardiotoxicity Associated with Anti-CD19 Chimeric Antigen Receptor T-Cell (CAR-T) Therapy: Recognition, Risk Factors, and Management. Diseases. 2021;9(1).
65. Lazăr DR, Farcaş AD, Blag C, Neaga A, Zdrenghea MT, Căinap C, et al. Cardiotoxicity: A Major Setback in Childhood Leukemia Treatment. Dis Markers. 2021;2021:8828410.
66. Kamaraju S, Mohan M, Zaharova S, Wallace B, McGraw J, Lokken J, et al. Interactions between cardiology and oncology drugs in precision cardio-oncology. Clin Sci (Lond). 2021;135(11):1333-51.
67. Neuendorff NR, Loh KP, Mims AS, Christofyllakis K, Soo WK, Bölükbasi B, et al. Anthracycline-related cardiotoxicity in older patients with acute myeloid leukemia: a Young SIOG review paper. Blood Adv. 2020;4(4):762-75.
68. Leerink JM, de Baat EC, Feijen EAM, Bellersen L, van Dalen EC, Grotenhuis HB, et al. Cardiac Disease in Childhood Cancer Survivors: Risk Prediction, Prevention, and Surveillance: JACC CardioOncology State-of-the-Art Review. JACC CardioOncol. 2020;2(3):363-78.
69. Saussele S, Haverkamp W, Lang F, Koschmieder S, Kiani A, Jentsch-Ullrich K, et al. Ponatinib in the Treatment of Chronic Myeloid Leukemia and Philadelphia Chromosome-Positive Acute Leukemia: Recommendations of a German Expert Consensus Panel with Focus on Cardiovascular Management. Acta Haematol. 2020;143(3):217-31.
70. Jamal FA, Khaled SK. The Cardiovascular Complications of Chimeric Antigen Receptor T Cell Therapy. Curr Hematol Malig Rep. 2020;15(2):130-2.
71. Herrmann J. Adverse cardiac effects of cancer therapies: cardiotoxicity and arrhythmia. Nat Rev Cardiol. 2020;17(8):474-502.
72. Giudice V, Vecchione C, Selleri C. Cardiotoxicity of Novel Targeted Hematological Therapies. Life (Basel). 2020;10(12).
73. Cook J, Litzow M. Advances in Supportive Care for Acute Lymphoblastic Leukemia. Curr Hematol Malig Rep. 2020;15(4):276-93.
74. Bhatia S. Genetics of Anthracycline Cardiomyopathy in Cancer Survivors: JACC: CardioOncology State-of-the-Art Review. JACC CardioOncol. 2020;2(4):539-52.
75. Puła B, Kępski J, Misiewicz-Krzemińska I, Szmit S. Left and right ventricular global longitudinal strain assessment together with biomarker evaluation may have a predictive and prognostic role in patients qualified for hematopoietic stem cell transplantation due to hematopoietic and lymphoid malignancies - a pilot study description. Cardiooncology. 2024;10(1):9.
